# Shape of attachment structures in parasitic isopodan crustaceans: the influence of attachment site and ontogeny

**DOI:** 10.7717/peerj.9181

**Published:** 2020-06-18

**Authors:** Serita van der Wal, Joachim T. Haug

**Affiliations:** 1 Zoomorphology Group, Department of Biology II, Ludwig-Maximilians-Universität München, Planegg-Martinsried, Germany; 2 GeoBio-Center, Ludwig-Maximilians-Universität München, München, Germany

**Keywords:** Cymothoida, Cymothoidae, Isopoda, Parasitism, Manca

## Abstract

Many fields of modern systematic biology are adult-centred. This is unfortunately also the case for Cymothoidae, an ingroup of parasitic forms of Isopoda, with fishes as hosts. Different ingroups of Cymothoidae have specialised appendages that enable their fish associated lifestyles, attaching to different sites on the body of the host. The extent to which these structures vary among species and in relation different sites of attachment, and between different ontogenetic stages, is explored here. This study presents the detailed descriptions, illustrations, comparisons, and analysis of a variety of attachment structures of 13 adult and immature specimens representing three major groups *Ceratothoa*, *Elthusa* and *Anilocra*, along with full focus, detailed photographs of all the examined life stages. The three groups exhibit different strategies attaching to mouth, gill and externally, respectively. A statistical representation of the morphology of the dactyli, used for attaching to the host, was performed. This included a critical comparison of 10 additional species documented in literature. This is the first comprehensive description and photographs of specialised appendage morphology of immatures of *Ceratothoa*, as well as the first detailed micrographs of embryonic stages of Cymothoidae, and the first lateral and ventral views of immature stages of the examined species. Immature specimens possess morphological characters that can be used to distinguish between different species, but cannot be accurately identified based on diagnostic characters of adults. Quantitative analysis indicates that ontogeny plays a major role in the shape of the attachment structures (e.g. dactyli).

## Introduction

Parasitism has evolved in many metazoan lineages, naturally also within the super-species-rich group Euarthropoda, including beetles, spiders, millipedes, shrimps and all their relatives (Kim, 1985; Müller, Eggert & Dressel, 1990; Eggleton & Belshaw, 1992; Boulton & Polis, 2002). Many parasites are rather small and not easy to encounter. Yet, some are quite large and astonishingly easy to spot. Examples for the latter case can be found in the crustacean group Cymothoidae Leach, 1818. Cymothoidae is an ingroup of Isopoda, the group that include woodlice, slaters and alike.

Representatives of Cymothoidae are ectoparasitic on fishes (Brusca, 1981; Kensley & Schotte, 1989; Colorni, Trilles & Golani, 1997; Hata et al., 2017), feeding on blood and tissue of the host (Bowman & Mariscal, 1968; Romestand & Trilles, 1977; Provenzano, 1983; Segal, 1987; Horton & Okamura, 2001). According to the World Register of Marine Species (Boyko et al., 2019 onwards), there are approximately 346 known and accepted species of Cymothoidae, excluding those that are in the need of revision. As most malacostracan crustaceans, their bodies are quite well sclerotised. Unlike many ingroups of parasitic forms of Euarthropoda, most representatives of Cymothoidae still retain a very ‘normal’ appearance well known for non-parasitic relatives from the group Isopoda (Schultz, 1969; Kensley, 1978).

Even though all of these appear rather unspecialised at first view, the posterior seven thorax appendages (posterior thoracopods, pereopods, pereiopods, peraeopods) are highly specialised for attaching to a host organism, in many cases, fishes. As ancestrally for malacostracans, these posterior thorax appendages consist of 7 elements, yet as in many forms of Isopoda the most proximal one, the coxa is largely immobile. The distal element (dactylus) is notably curved, sickle-like, large and pointed, specialised for attachment to a fish host. This is in contrast to the smaller, slender and more straight distal elements of non-parasitic forms of Isopoda (Nagler, Hyžný & Haug, 2017).

Within Cymothoidae, there are four major strategies of where to attach on their fish host: (1) the external surface, as representatives of *Anilocra* Leach, 1818 and *Nerocila* Leach, 1818 (Nagler et al., 2016; Welicky et al., 2017); (2) inside the mouth (‘buccal cavity’), as representatives of *Ceratothoa* Dana, 1852 and *Cymothoa* Fabricius, 1793 (González et al., 2019; Vigneshwaran, Ravichandran & Prema, 2019); inside the gill chamber (‘branchial cavity’), as representatives *Elthusa* Schioedte & Meinert, 1884 and *Mothocya* Costa in Hope, 1851 (De Souza et al., 2019; Van der Wal, Smit & Hadfield, 2019); or (4) burrowing inside the muscle tissue of the host, as representatives of *Ichthyoxenos* Herklots, 1870 and *Riggia* Szidat, 1948 (Tsai & Dai, 1999; Azevedo et al., 2006).

The site, exact position and orientation of attachment to a host is either species specific or is used to characterise groups of species (mostly genera). Depending on the site of attachment, the body symmetry of an adult individual of Cymothoidae may be distorted (Brusca, 1975). Individuals that attach inside a cavity tend to be asymmetrical as a result of the space restriction and positioning. Adult specimens of species attaching to the gills typically have a curved body either to the left or right (Brusca, 1981), while adults of species attaching in the mouth are elongated and more compressed laterally, that is more cylindrical in shape (Hadfield, 2012). The growth of externally attaching forms are not that heavily restricted by space and most remain symmetrical (Kensley, 1978) and dorso-ventrally compressed, in order to withstand currents and water flow. In some cases, deformation may occur (Nagler et al., 2016 and references therein).

Recently, quantitative methods have shown that the shape of the distal hook, the dactylus, is affected in evolutionary terms, by the site of attachment. In other words, the shape of the hook appears to be correlated to a specific parasitic strategy (Baillie et al., 2019).

The modern biology is in many fields of research, an adult-centred one (Minelli, Boxshall & Fusco, 2013; Haug & Haug, 2019). This also holds true for Cymothoidae. Although we know many aspects of the adult ecology, we still lack information on the immatures. Yet, it is exactly during this stage that the organisms manage to find and infect a host. In the case of Cymothoidae, sexes can also be related to ontogeny, a male can transform into a female, in this case making the male to a specific ontogenetic stage.

Here we describe some new immatures together with corresponding adults. We qualitatively compare morphological changes during ontogeny. Together with data from the literature, we attempt a quantitative comparison of the hook-shaped attachment structures to compare the influence of ontogeny vs. the influence of attachment site.

## Materials and Methods

### Material

Selected material was loaned from the Centrum für Naturkunde, Zoological Museum (CeNak), Hamburg and the Bavarian State Collection of Zoology (ZSM), Munich. Specimens were chosen (1) to represent forms from groups that are known to attach to different sites on their hosts, and (2) to be represented by several life stages of a single species. In order to increase the sample size of immatures, which are often not collected and deposited along with adult forms, isolated immature specimens were also considered.

### Documentation methods

Specimens were individually documented and photographed, with methods depending on the size of each specimen. Larger specimens (>5.0 mm) were photographed using composite-super-macro photography, with a polarising filter (Hama PL circular M58 (IV) IV 4 58 mm). This was done by alternating between a Canon EF-S 18–55 mm Macro lens and Canon MP-E 65 mm f/2.8 1–5x Macro lens, with a two piece Yongnuo YN24EX E TTL Macro Flash. Exact methods follow those of Haug et al. (2011). Smaller specimens were photographed using a Keyence VHX-6000 Digital Microscope. Specimens were documented in dorsal, lateral, ventral and antero-ventral orientation. Total length and width of each specimen were recorded.

After documentation of entire specimens, some structures (sensory appendages, mouthparts and pereopods) were dissected. In most cases, only the right side of the specimens were dissected, unless the specific body part was already missing on the right side, or had been damaged due to fragility. The dissected parts were cleaned and mounted to be viewed and photographed with the aid of an inverse fluorescence microscope BZ-9000 (BIOREVO, Keyence) using fluorescence (with DAPI filter) and Brightfield setting (methods follow Nagler & Haug (2016)). This method of photography mirrors the photographed parts, as the objectives are below the viewing table. The combination of these settings were used to achieve full visibility of small structures, such as setae, along with the overall structure. After documentation, the dissected parts were collected and stored in separate vials with the collection number of the specimen.

To achieve fully focused images, image stacks were created using the software program Combine ZP and panorama images were created with the aid of Adobe Photoshop Elements 11. Image editing and arrangement of figure plates were made with the software program Affinity Photo. The images of left-sided body parts were mirrored, to ensure consistency throughout the plates. These alterations are mentioned within the *Remarks* section of the specific specimen that it applies to.

### Descriptions

Descriptions of all specimens were done by recording character information using the free software package Descriptive Language for Taxonomy (DELTA^©^). These descriptions contain detailed information on mouthparts and pereopod sub-structures, which are those considered to be most specialised for a parasitic lifestyle, most of which have not been described or photographed in detail before. For the original, full species descriptions, see the Remarks section of each respective species. The exported RTF files converted the descriptions from DELTA, into text format. Descriptions were done for the following structures: Antennulae, antennae, mandibles, maxillulae, maxillae and maxillipeds. In addition, pereopod descriptions consisted of information regarding the length to width ratios of the distal 6 elements (basipod, ischium, merus, carpus, propodus, dactylus) of pereopods 1, 4, 6 and 7. This pereopod selection was bases on: (1) Pereopods 1 and 7 are most often described and illustrated as they sometimes containing species or group specific characters. (2) Pereopod 6 was incorporated since the immature stage 1 and 2 still lack 7, but we still need a signal for the differentiation along the body. (3) Even though all pereopod appendages 1–7 of Cymothoidae are specialised for their parasitic lifestyle, other groups such as the Aegidae have only the first 3 pairs specialised for attachment to a host. For a future larger scaled comparison, pereopod 4 will be important. Descriptions of embryonic stage individuals were mainly based on the overall body size and shape, as well as the visible segmentation of developing structures.

### Measurements and statistics

For descriptions of examined specimens, measurements of all dissected appendages were recorded in terms of maximum total length and maximum total width (in mm) of each article or element, from which ratios were calculated. Ratio values were rounded up to one decimal. A statistical comparative analysis was generated to present the variation in shape of dactyli of pereopods 1 and 6. In order to increase the dataset for the analysis, illustrations of appendages from literature were included. The illustrations of appendages from literature were selected based on: (1) The site of attachment to the host; (2) The availability of illustrations for both pereopod appendages 1 and 6 of an immature and an adult specimen. A total of 15 species (5 each for the 3 attachment sites), were selected.

Dactyli from literature illustrations were redrawn using Adobe Illustrator (see Figs. S1–S4). The statistical representation of the variation in pereopod morphology through ontogeny, was done with the aid of the software SHAPE (© National Agricultural Research Organization of Japan). An elliptic Fourier analysis was performed, following Braig et al. (2019), to acquire values for a principle component analysis (PCA). The concave outline of dactyli proximal ends (as illustrated by almost all literature sources) of the selected illustrations proved to cause errors during the SHAPE analysis and had to be altered in such a way that it does not affect the principle components of interest. The solution was to add a rounded (convex) outline to the proximal end of the dactylus article. For the same reason, setae on pereopod articles were also not illustrated. For the publications from which these illustrations were redrawn, see Table S1. The PCA values (see Fig. S5) and plots were visualised with the aid of R-statistics 3.6.1 in R-studio.

### Terminology

Specialised terminology developed for ingroups, often ignores traditions in other groups, prohibiting communication beyond specific taxonomic border. This can be well seen in sub-fields of Entomology, but also in Carcinology. Terminology for Isopoda is no exception. Here we use ‘Isopodology’ specific terms for structures. These can be compared to, but often differ from, the more general malacostracan and crustacean terms. For a well explanatory and comparative figure on the variation in terms used between Isopodologists and crustacean researchers, see Nagler, Eiler & Haug (2019). On the long run it needs to be an aim to provide a framework allowing comparison beyond narrow taxonomic border.

## Results

*Ceratothoa* Dana, 1852

Refer to Hadfield, Bruce & Smit (2016) for full synonymy.

***Ceratothoa* sp.**

Material examined

Four specimens of different ontogenetic stages were examined. K23191 gravid female (65.0 mm total length, 24.0 mm wide); K23191b male (25.5 mm total length, 11.5 mm wide); K23191c immature stage 2 (5.7 mm total length, 2 mm wide); K23191d embryo (2.2 mm total length, 1.7 mm wide). Originally collected in Port Elizabeth, South Africa by J.L Drége on 25.05.1903; deposited at CeNak.

Gravid female

**Figure 1:**
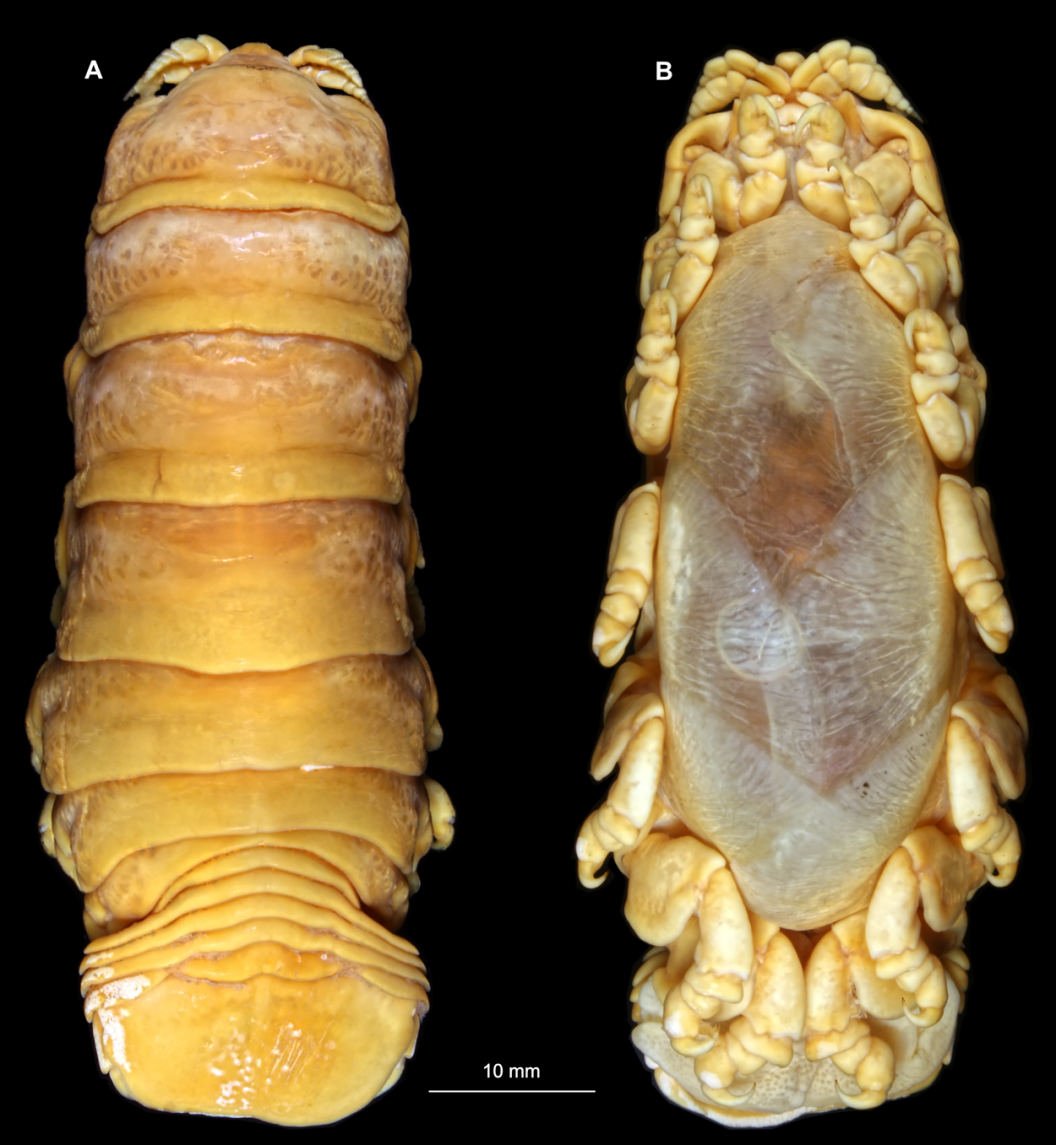
*Ceratothoa* sp. gravid female (K23191). (A) Dorsal view; (B) Ventral view.

**Figure 2:**
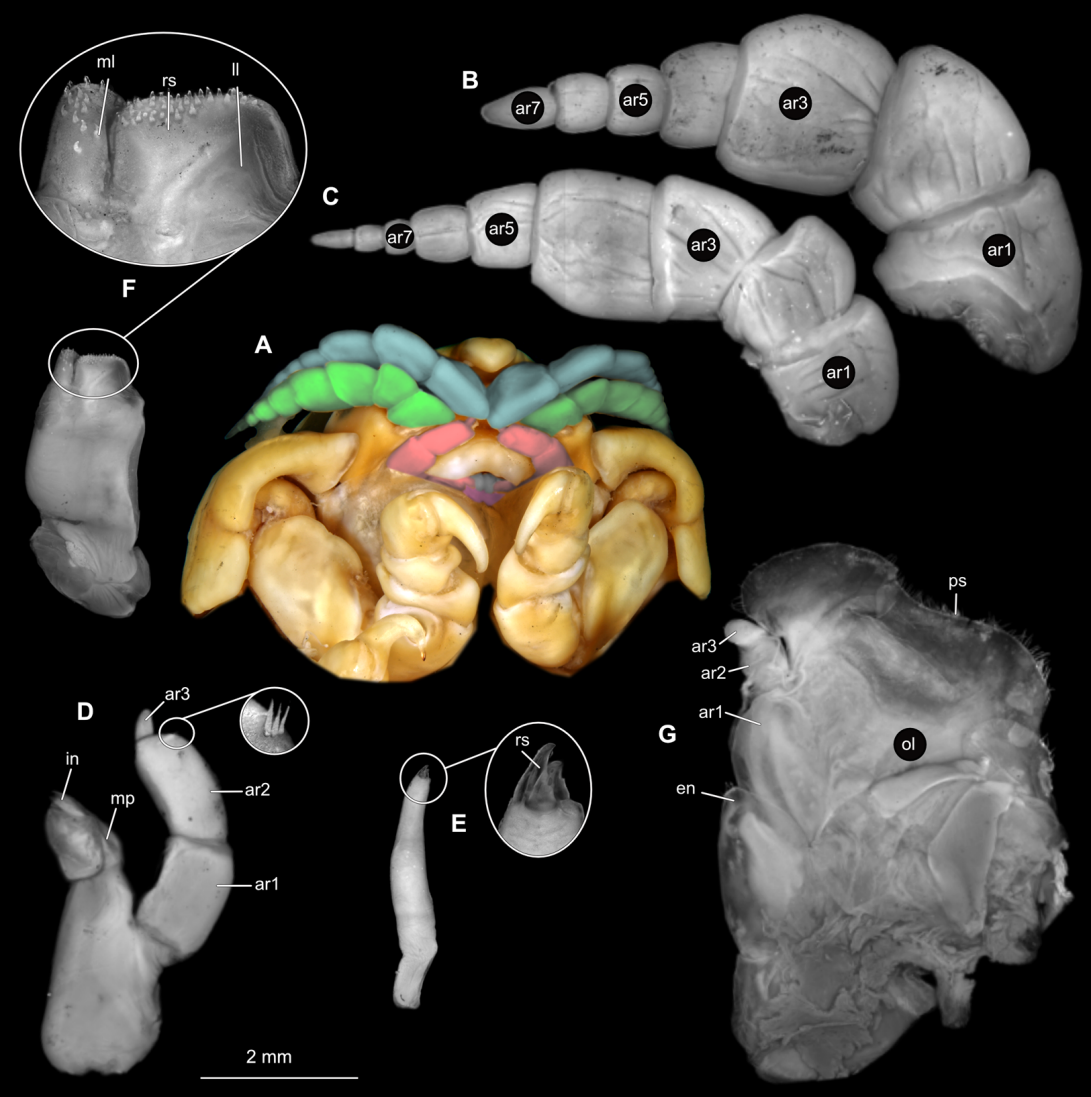
*Ceratothoa* sp. gravid female (K23191). (A) Ventral view of functional head with mouthpart positioning. (B) Antennula. (C) Antenna. (D) Mandible. (E) Maxillula. (F) Maxilla. (G) Maxilliped. ar1, article 1; ar2, article 2; ar3, article 3; ar5, article 5; ar7, article 7; en, endite; mp, molar process; in, incisor; rs, robust setae; ml, medial lobe; ll, lateral lobe; ol, oostegital lobe; ps, plimose setae.

**Figure 3:**
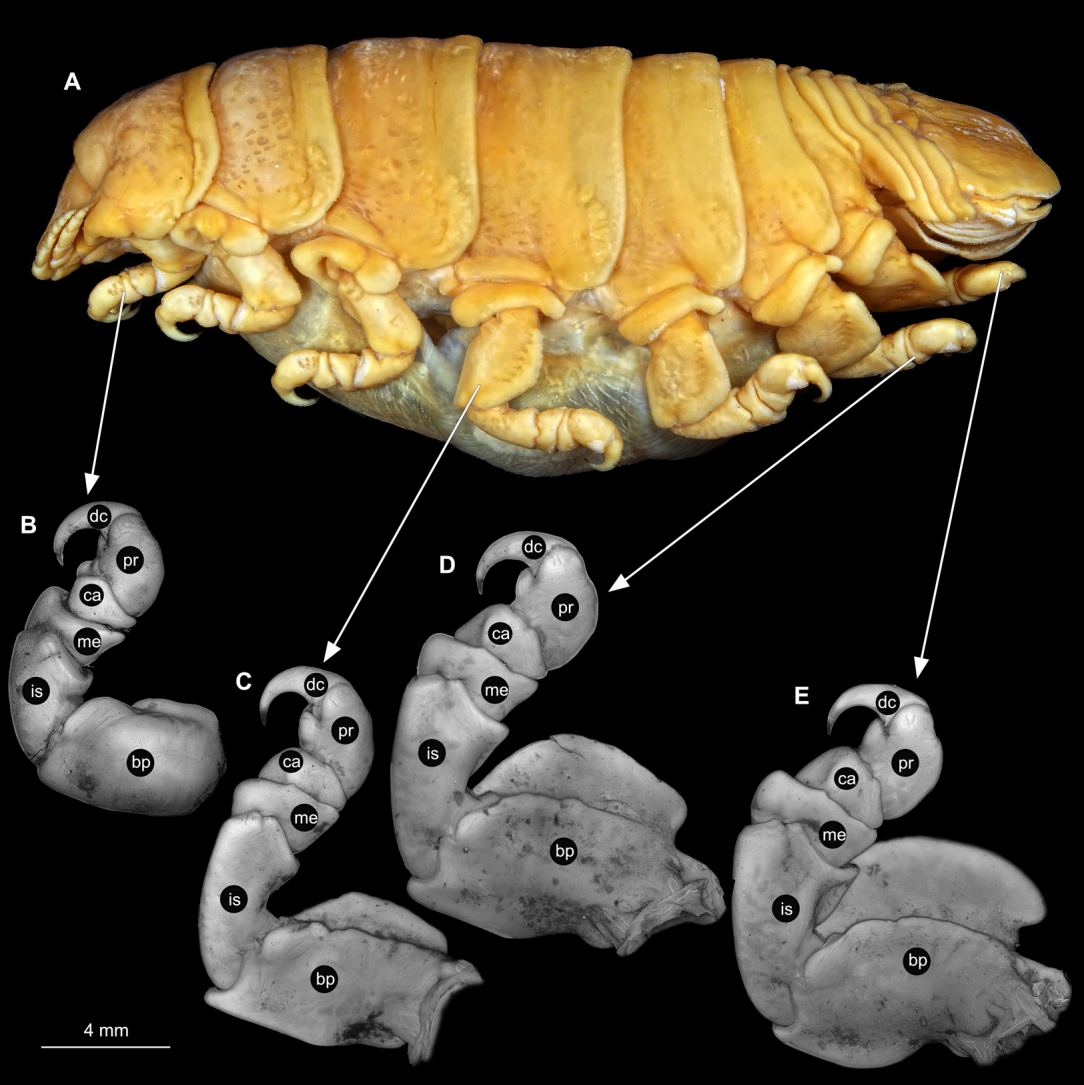
*Ceratothoa* sp. gravid female (K23191). (A) Lateral view. (B) Pereopod 1. (C) Pereopod 4. (D) Pereopod 6. (E) Pereopod 7. bp, basipod; is, ischium; me, merus; ca, carpus; pr, propodus; dc, dactylus.

Body longer than wide, 2.7x (Figs. 1A and 1B). Antennula 9.43 mm long, consists of 7 articles; articles 1 & 2 distinct, without setae (Fig. 2B); articles longer than wide, article 1, 0.9x; article 2, 0.9x; article 3, 0.9x; article 4, 0.6x. Antenna 8.55 mm long, consists of 9 articles; shorter than antennula, thinner than antennula; without setae (Fig. 2C); articles longer than wide, article 1, 0.9x; article 2, 0.7x; article 3, 0.9x; article 4, 0.8x. Mandible (Fig. 2D) with proximal coxa, longer than wide in proximal-distal axis, 2.4x; medially drawn out into gnathal edge, differentiated as molar surface and acute incisor; distal palp with 3 articles, palp articles longer than wide, article 1, 1.6x; article 2, 1.8x, with 3 setae; article 3, 1.6x. Maxillula elongate without subdivision (Fig. 2E); longer than wide, 5.3x; at the tip, original median edge, with 4 robust setae. Maxilla with 2 distinct lobes (Fig. 2F); longer than wide, 2.3x; medial lobe 0.27 mm wide, with 13 setae; lateral lobe 0.72 mm wide, with 34 setae. Maxilliped with 3 articles (Fig. 2G); with medial endite (originating from not dissected basipod), longer than wide in proximal-distal axis, 2.2x, with 4 setae; with lobe-like oostegite, lined with multiple plumose setae; articles longer than wide, article 1 (ischium) margins difficult to discern; article 2 (merus), 0.8x; article 3 (carpus), 1.5x, without setae. Posterior thorax appendages, 7 pairs, each with 7 articles, coxae not dissected (Figs. 3A–3E). Distal 6 articles forming functional leg. Pereopod 1 (Fig. 3B) basipod longer than wide, 1.5x; ischium longer than wide, 1.4x; merus longer than wide, 0.6x; carpus longer than wide, 0.6x; propodus longer than wide, 1.4x; dactylus longer than wide, 2.6x; entire appendage without setae. Pereopod 4 (Fig. 3C) basipod longer than wide, 1.6x; ischium longer than wide, 1.7x; merus longer than wide, 0.5x; carpus longer than wide, 0.5x; propodus longer than wide, 1.4x; dactylus longer than wide, 2.3x; entire appendage without setae. Pereopod 6 (Fig. 3D) basipod longer than wide, 1.4x; ischium longer than wide, 1.8x; merus longer than wide, 0.5x; carpus longer than wide, 0.6x; propodus longer than wide, 1.0x; dactylus longer than wide, 2.7x; entire appendage without setae. Pereopod 7 (Fig. 3E) basipod longer than wide, 1.3x; ischium longer than wide, 1.5x; merus longer than wide, 0.4x; carpus longer than wide, 0.7x; propodus longer than wide, 2.5x; dactylus longer than wide, 2.4x; entire appendage without setae.

Male

**Figure 4:**
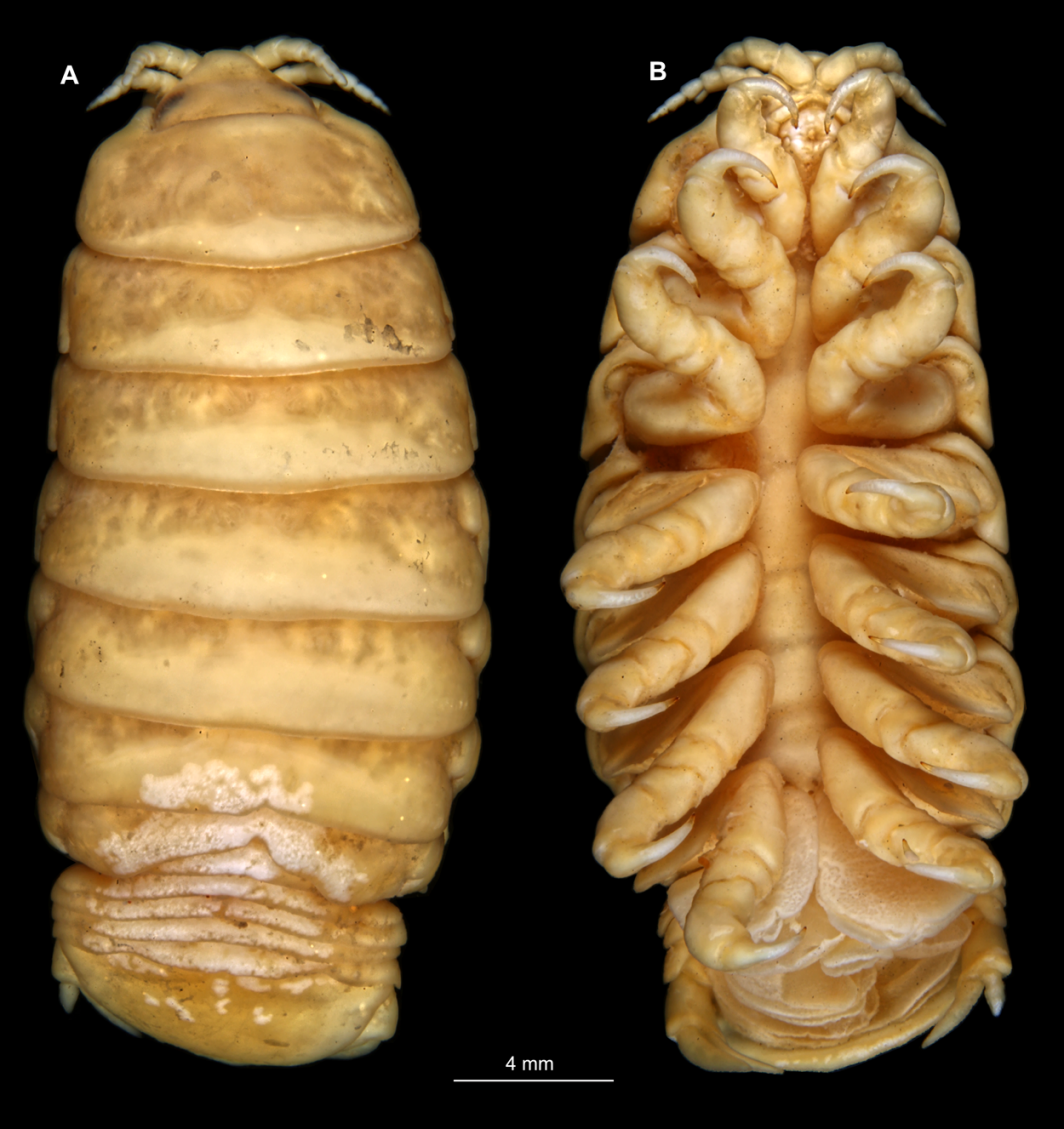
*Ceratothoa* sp. male (K23191b). (A) Dorsal view. (B) Ventral view.

**Figure 5:**
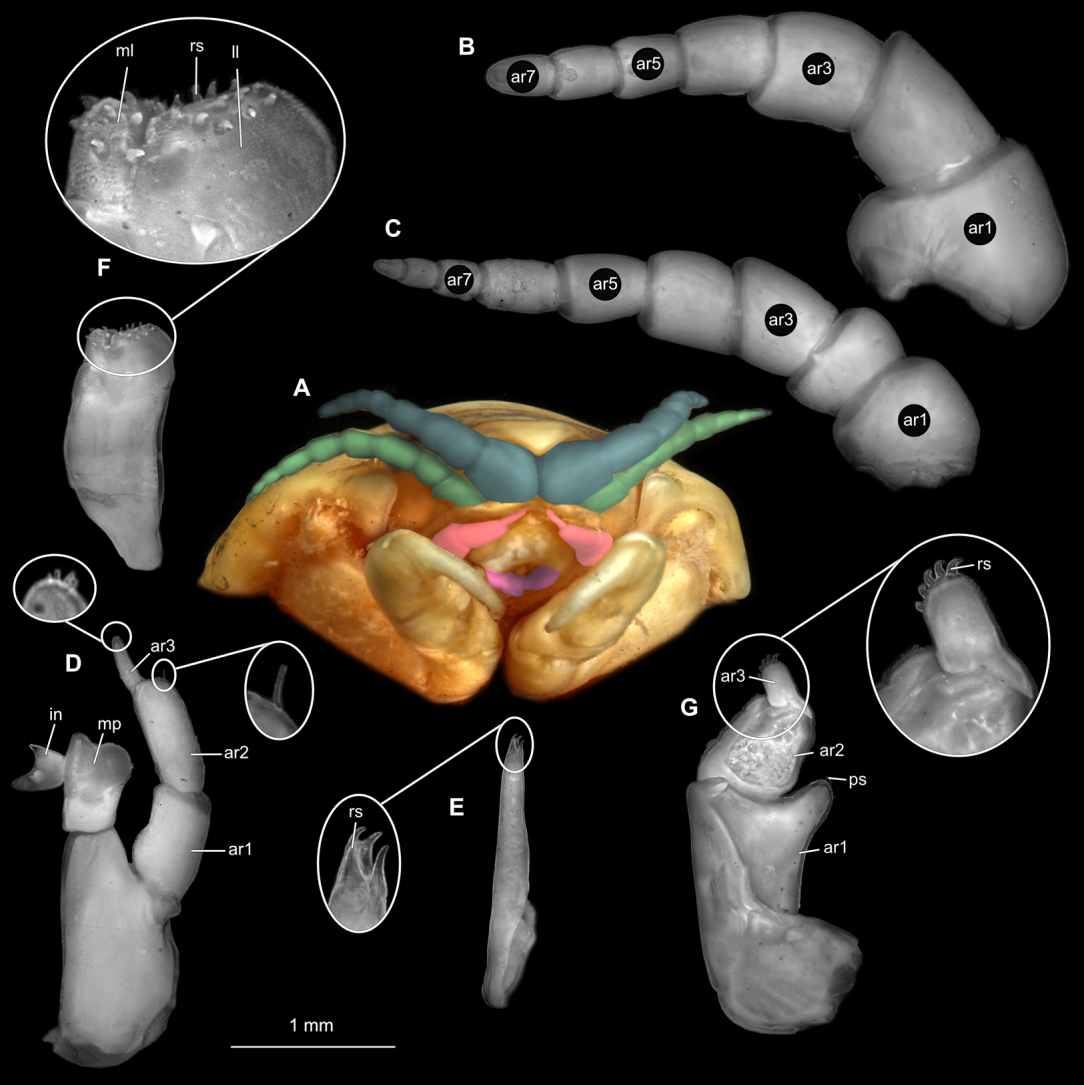
*Ceratothoa* sp. male (K23191b). (A) Ventral view of functional head with mouthpart positioning. (B) Antennula. (C) Antenna. (D) Mandible. (E) Maxillula. (F) Maxilla. (G) Maxilliped. ar1, article 1; ar2, article 2; ar3, article 3; ar5, article 5; ar7, article 7; mp, molar process; in, incisor; rs, robust setae; ml, medial lobe; ll, lateral lobe; ps, plimose setae.

**Figure 6:**
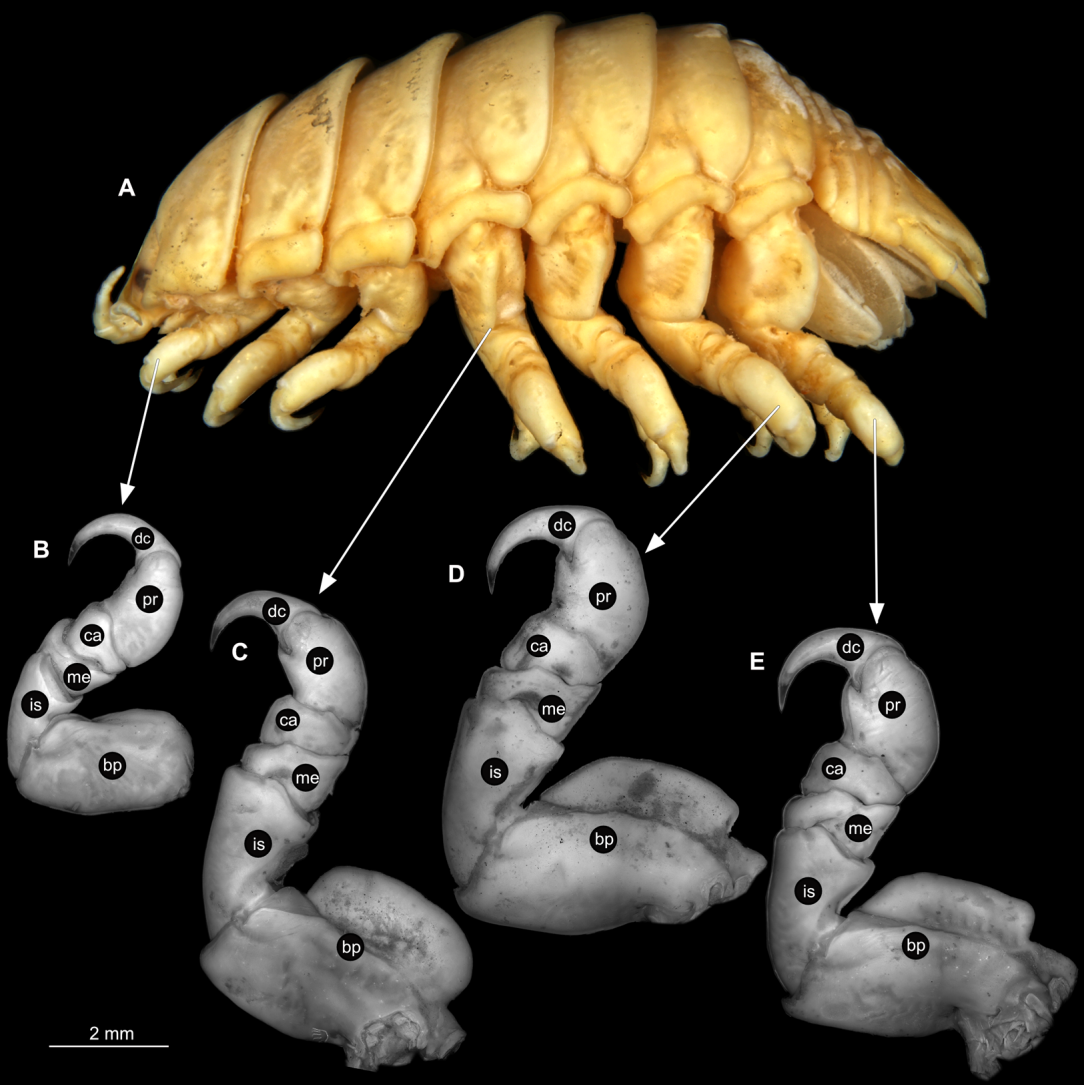
*Ceratothoa* sp. male (K23191b). (A) Lateral view. (B) Pereopod 1. (C) Pereopod 4. (D) Pereopod 6. (E) Pereopod 7. bp, basipod; is, ischium; me, merus; ca, carpus; pr, propodus; dc, dactylus.

Body longer than wide, 2.2x (Figs. 4A and 4B). Antennula 4.20 mm long, consists of 7 articles; articles 1 & 2 distinct (Fig. 5B); articles longer than wide, article 1, 0.9x; article 2, 1.2x; article 3 as long as wide; article 4 as long as wide. Antenna 3.96 mm long, consists of 9 articles; shorter than antennula, thinner than antennula, without setae (Fig. 5C); articles longer than wide, article 1, 0.9x; article 2, 0.6x; article 3, 1.2x; article 4 as long as wide. Mandible with proximal coxa; longer than wide in proximal-distal axis, 2.9x (Fig. 5D); medially drawn out into gnathal edge, differentiated as molar surface and acute incisor; distal palp with 3 articles, palp articles longer than wide, article 1, 1.4x; article 2, 2.5x, with 1 seta; article 3, 3.2x. Maxillula elongate without subdivision; longer than wide, 1.7x (Fig. 5E); at the tip, original median edge, with 4 robust setae. Maxilla with 2 distinct lobes; longer than wide, 2.5x (Fig. 5F); medial lobe 0.14 mm wide, with 7 setae; lateral lobe 0.40 mm wide, with 14 setae. Maxilliped with 3 articles (Fig. 5G); without endite, without oostegite lobe; articles longer than wide, article 1 (ischium), 1.7x; article 2 (merus), 1.1x; article 3 (carpus), 1.7x, with 5 robust setae. Posterior thorax appendages, 7 pairs, each with 7 articles, coxae not dissected (Figs. 6A–6E). Distal 6 articles forming functional leg. Pereopod 1 (Fig. 6B) basipod longer than wide, 1.7x; ischium longer than wide, 1.9x; merus longer than wide, 0.6x; carpus longer than wide, 0.7x; propodus longer than wide, 1.6x; dactylus longer than wide, 2.8x; entire appendage without setae. Pereopod 4 (Fig. 6C) basipod longer than wide, 1.3x; ischium longer than wide, 1.6x; merus longer than wide, 0.7x; carpus longer than wide, 0.5x; propodus longer than wide, 1.6x; dactylus longer than wide, 2.3x; entire appendage without setae. Pereopod 6 (Fig. 6D) basipod longer than wide, 1.7x; ischium longer than wide, 1.7x; merus longer than wide, 0.6x; carpus longer than wide, 0.7x; propodus longer than wide, 1.4x; dactylus longer than wide, 2.5x; entire appendage without setae. Pereopod 7 (Fig. 6E) basipod longer than wide, 1.5x; ischium longer than wide, 1.6x; merus longer than wide, 0.6x; carpus longer than wide, 0.6x; propodus longer than wide, 1.5x; dactylus longer than wide, 2.3x; entire appendage without setae.

Immature stage 2

**Figure 7:**
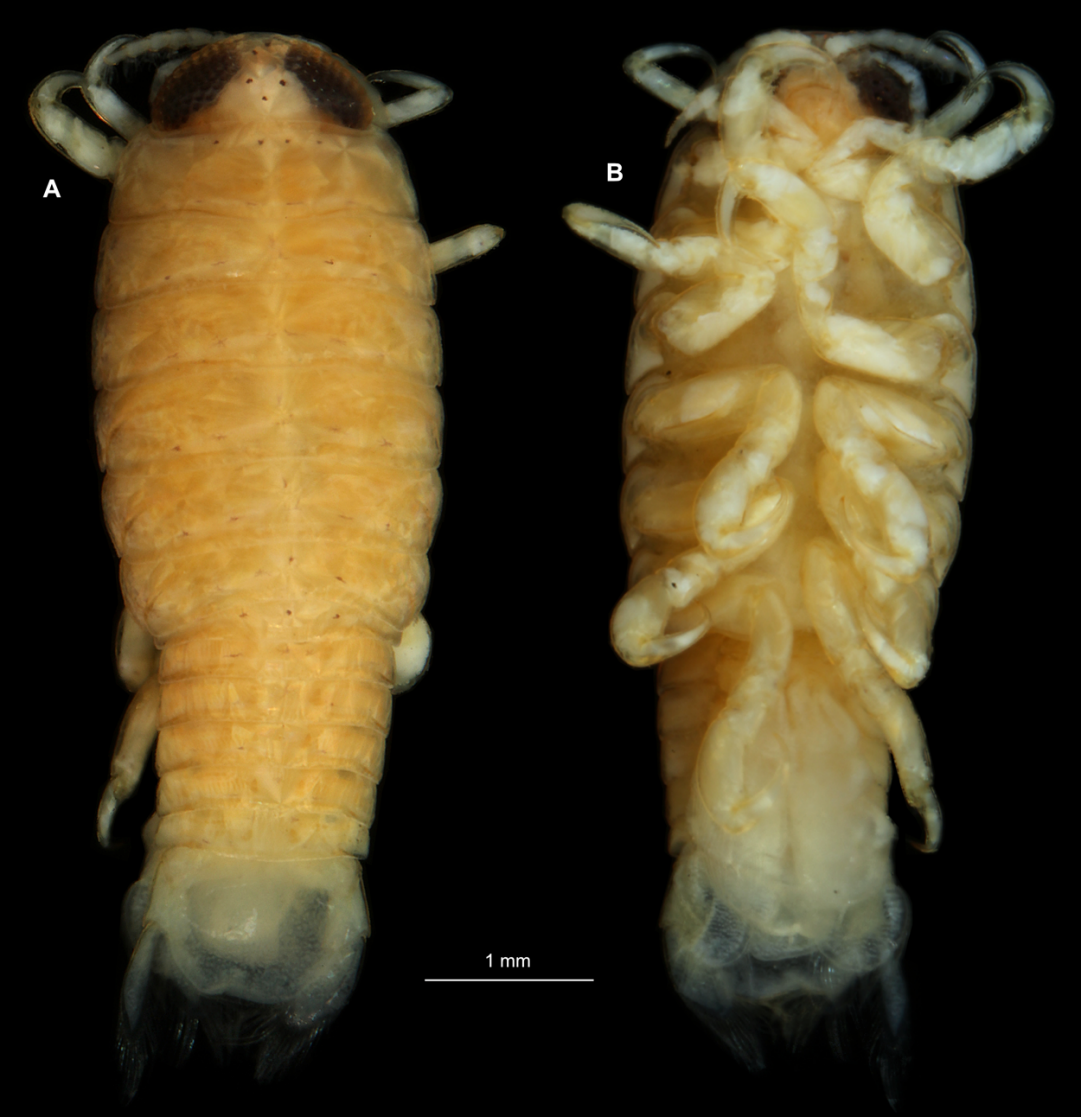
*Ceratothoa* sp. immature stage 2 (K23191c). (A) Dorsal view. (B) Ventral view.

**Figure 8:**
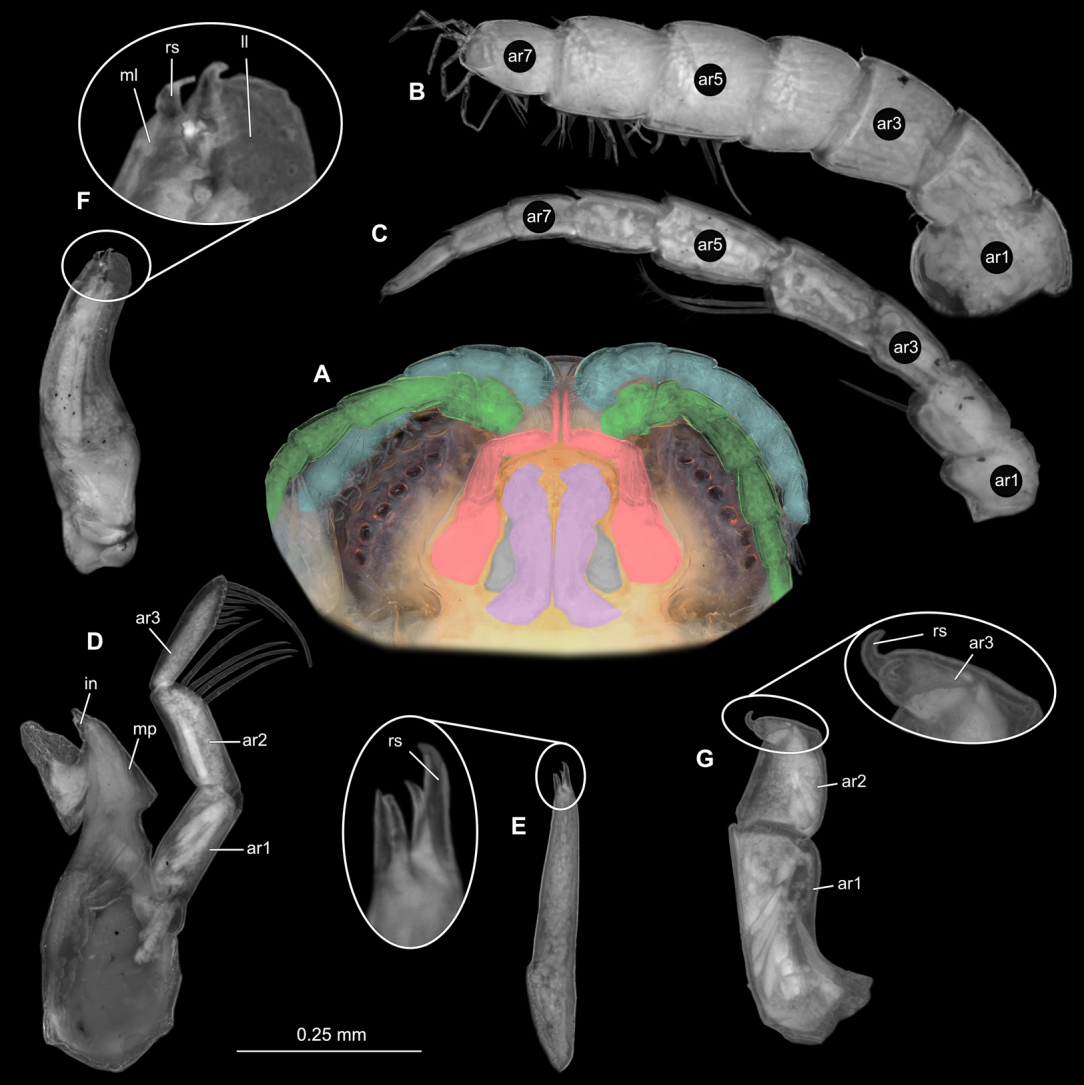
*Ceratothoa* sp. immature stage 2 (K23191c). (A) Ventral view of functional head with mouthpart positioning. (B) Antennula. (C) Antenna. (D) Mandible. (E) Maxillula. (F) Maxilla. (G) Maxilliped. ar1, article 1; ar2, article 2; ar3, article 3; ar5, article 5; ar7, article 7; mp, molar process; in, incisor; rs, robust setae; ml, medial lobe; ll, lateral lobe.

**Figure 9:**
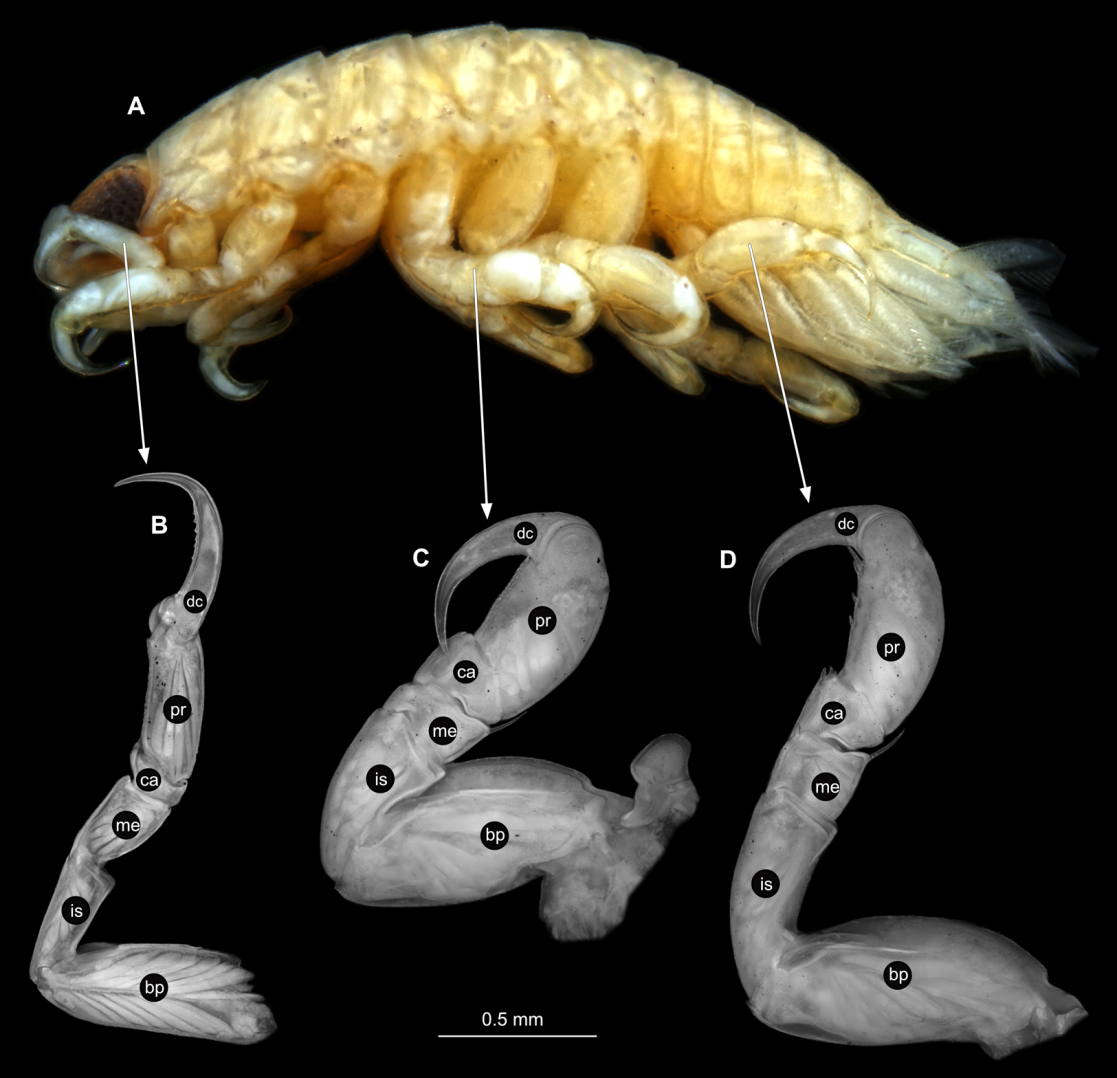
*Ceratothoa* sp. immature stage 2 (K23191c). (A) Lateral view. (B) Pereopod 1. (C) Pereopod 4. (D) Pereopod 6. bp, basipod; is, ischium; me, merus; ca, carpus; pr, propodus; dc, dactylus.

Body longer than wide, 2.9x (Figs. 7A and 7B). Antennula 0.92 mm long, consists of 7 articles; articles 1 & 2 distinct; with single, simple setae on articles 1–4 and tufts of setae on articles 5–7 (Fig. 8B); articles longer than wide, article 1, 0.6x; article 2, 0.9x; article 3, 0.8x; article 4, 0.8x. Antenna 1.07 mm long, consists of 9 articles; longer than antennula, thinner than antennula; with plumose setae on articles 2, 4–9 (Fig. 8C); articles longer than wide, article 1, 0.8x; article 2, 1.1x; article 3, 1.5x; article 4, 1.6x. Mandible with proximal coxa, longer than wide in proximal-distal axis, 2.4x (Fig. 8D); medially drawn out into gnathal edge, differentiated as molar surface and acute incisor; distal palp with 3 articles, palp articles longer than wide, article 1, 2.4x; article 2, 2.4x, with 4 setae; article 3, 3.9x, with 7 setae. Maxillula elongate without subdivision (Fig. 8E); longer than wide, 6.4x; at the tip, original median edge, with 4 robust setae. Maxilla with 2 partly conjoined lobes; longer than wide, 3.6x (Fig. 8F); medial lobe 0.03 mm wide, with 1 seta; lateral lobe 0.04 mm wide, with 1 seta. Maxilliped with 3 articles (Fig. 8G); without endite, without oostegite lobe; all articles longer than wide, article 1 (ischium), 2.4x; article 2 (merus), 1.2x; article 3 (carpus), 2.6x, with 2 robust setae. Posterior thorax appendages, 6 pairs, each with 7 articles, coxae not dissected. Distal 6 articles forming functional leg (Figs. 9A–9D). Pereopod 1 (Fig. 9B) basipod longer than wide, 2.7x; ischium longer than wide, 2.4x; merus longer than wide, 1.5x; carpus longer than wide, 0.6x; propodus longer than wide, 2.7x; dactylus longer than wide, 4.2x; entire appendage without setae. Pereopod 4 (Fig. 9C) basipod longer than wide, 2.0x; ischium longer than wide, 1.7x; merus longer than wide, 0.5x; carpus longer than wide, 0.7x; propodus longer than wide, 1.7x; dactylus longer than wide, 3.5x; with single spine-like seta on merus. Pereopod 6 (Fig. 9D) basipod longer than wide, 2.4x; ischium longer than wide, 2.7x; merus longer than wide, 0.3x; carpus longer than wide, 0.7x; propodus longer than wide, 2.1x; dactylus longer than wide, 3.4x; with 6 setae with spine-like setae on merus, carpus and propodus.

Embryo

**Figure 10:**
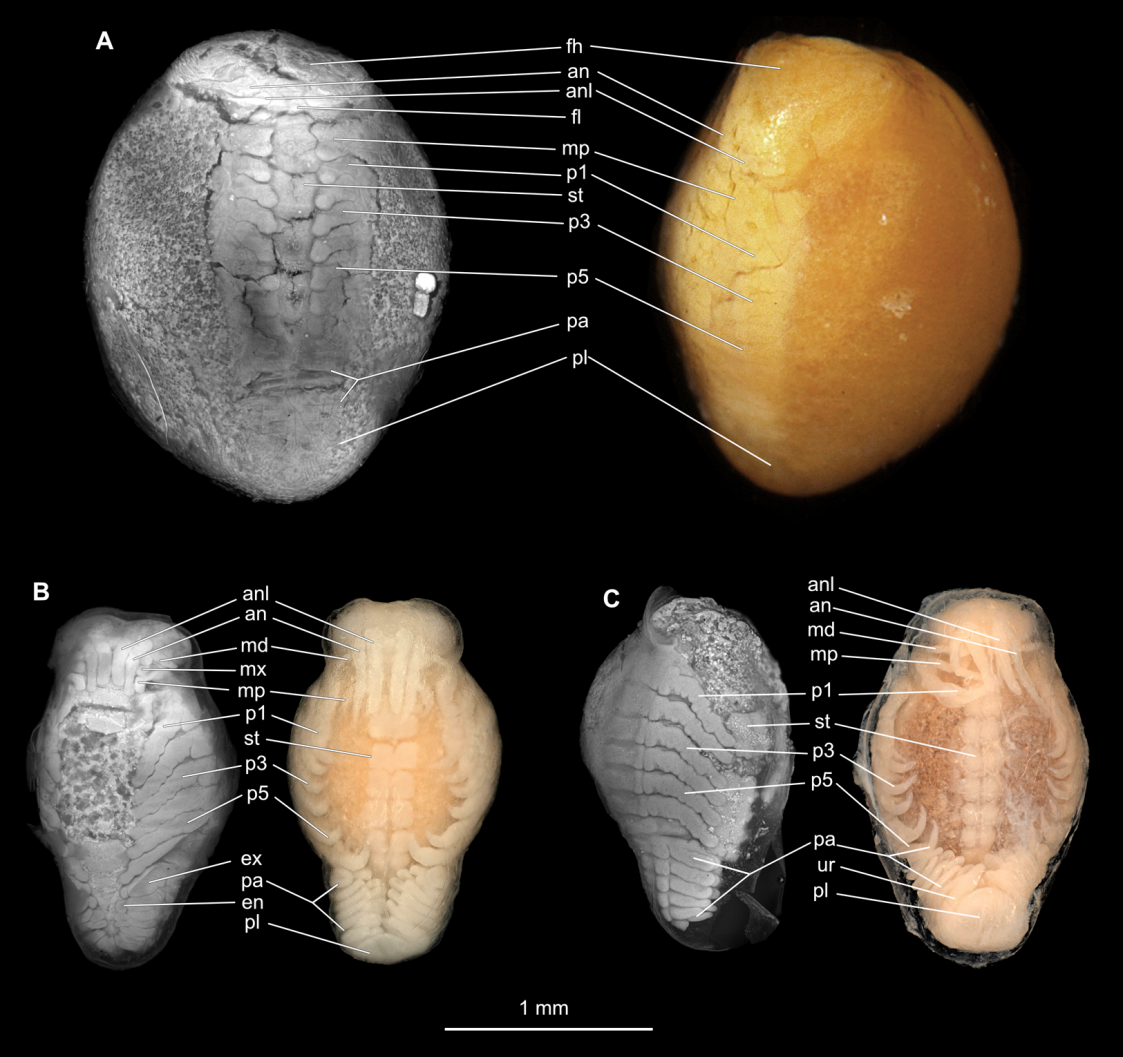
Embryonic stages: fluorescent and composite-super-macro photographs. (A) *Ceratothoa* sp. (K23191). (B) *Elthusa vulgaris* (K26553c). (C) *Anilocra physodes* (K19211d). fh, functional head; an, antenna; anl, antennula; fl, frontal lamina; md, mandible; mx, maxilla; mp, maxilliped; st, sternite; p1, pereopod 1; p3, pereopod 3; p5, pereopod 5; pa, pleon appendage; ex, pleon attachment exopod; en, pleon appendage endopod; pl, pleotelson; ur, uropod.

Body round, longer than wide, 1.3x, incapsulated in membrane (Fig. 10A). Antennula 0.24 mm long; longer than wide, 3.7x. Antenna, 0.30 mm long; longer than wide 3.4x. Maxilliped, palp 0.20 mm long; longer than wide, 1.3x. Pereopod 1 longer than wide, 2.6x. Pereopod 4 longer than wide, 2.0x. Pereopod 6, longer than wide, 1.8x. Eyes not visible.

***Ceratothoa gaudichaudii* (Milne Edwards, 1840) *species inquirendum***

Material examined

*Ceratothoa gaudichaudii* (Milne Edwards, 1840) immature stage 1 (4.4 mm total length, 1.5 mm wide); loc: fishmarket in Huaraz, Peru; fish host: Gadidae species; date: 29.08.1989; deposited at the Zoological Museum, Hamburg, CeNak (K34618).

Immature stage 1

**Figure 11:**
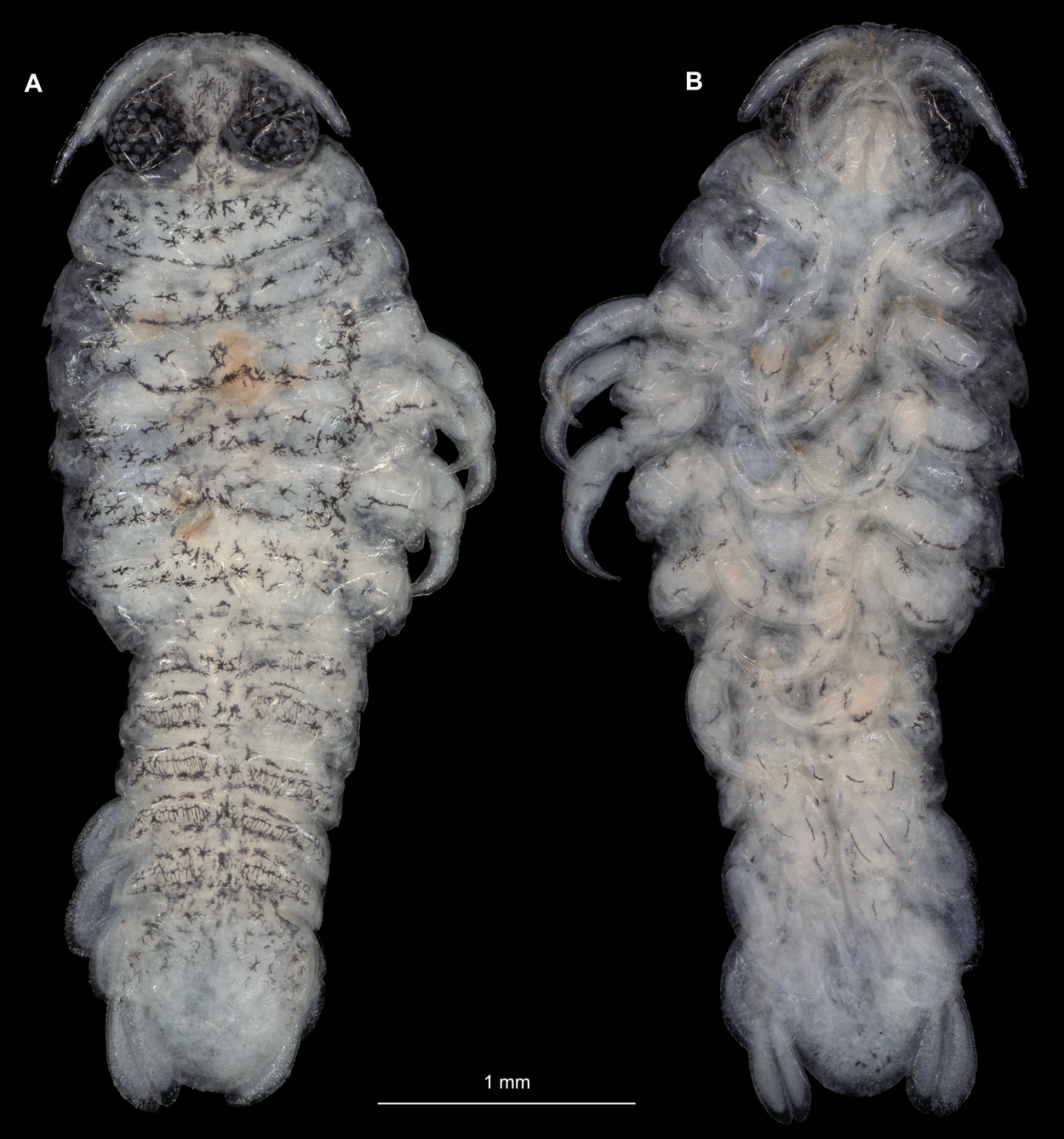
*Ceratothoa gaudichaudii* (Milne Edwards, 1840) immature stage 1 (K39618). (A) Dorsal view. (B) Ventral view.

**Figure 12:**
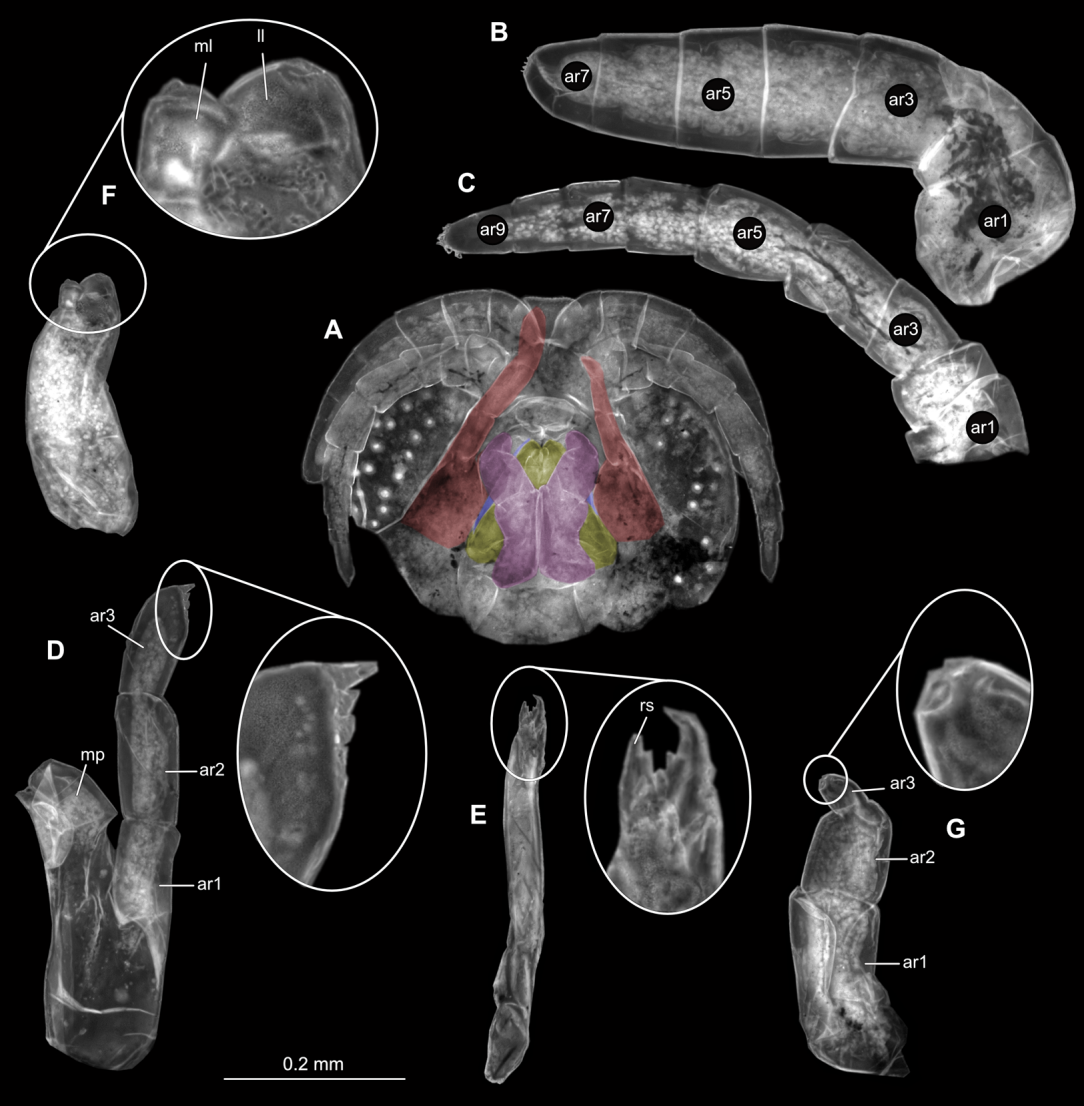
*Ceratothoa gaudichaudii* (Milne Edwards, 1840) immature stage 1 (K39618). (A) Ventral view of functional head with mouthpart positioning; (B) Antennula. (C) Antenna. (D) Mandible. (E) Maxillula. (F) Maxilla. (G) Maxilliped. ar1, article 1; ar2, article 2; ar3, article 3; ar5, article 5; ar7, article 7; mp, molar process; rs, robust setae; ml, medial lobe; ll, lateral lobe.

**Figure 13:**
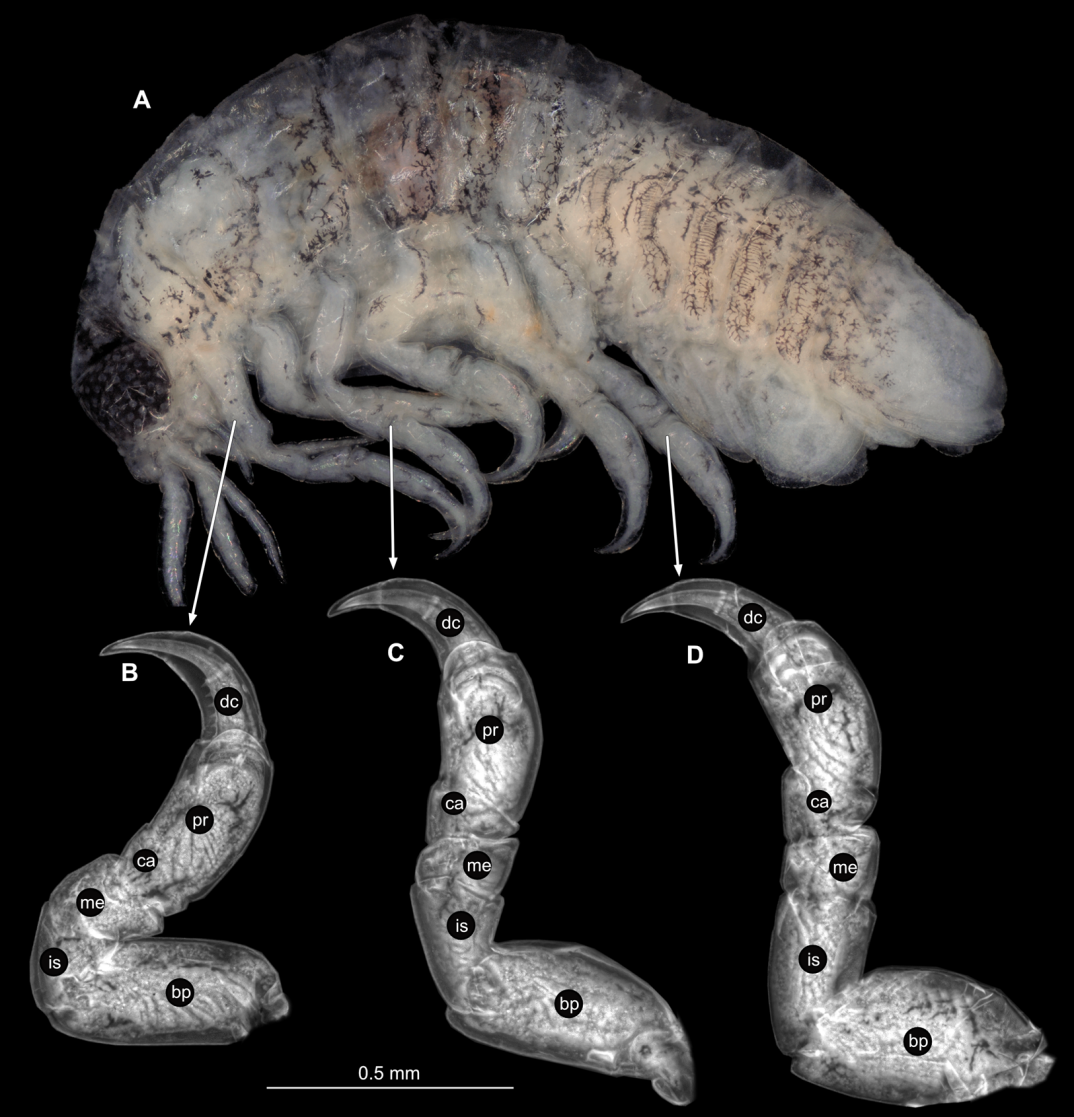
*Ceratothoa gaudichaudii* (Milne Edwards, 1840) immature stage 1 (K39618). (A) Lateral view. (B) Pereopod 1. (C) Pereopod 4. (D) Pereopod 6. bp, basipod; is, ischium; me, merus; ca, carpus; pr, propodus; dc, dactylus.

Body longer than wide, 2.9x (Figs. 11A and 11B). Antennula 0.68 mm long, consists of 7 articles; articles 1 & 2 distinct; with 4 simple setae on terminating article (Fig. 12B); articles longer than wide; article 1, 07x; article 2, 0.9x; article 3, 0.8x; article 4, 0.7x. Antenna 0.73 mm long, consists of 9 articles; longer than antennula, thinner than antennula (Fig. 12C); with 9 simple setae on terminating article; articles longer than wide; article 1, 0.6x; article 2, 0.5x; article 3, 1.0x; article 4, 1.1x. Mandible with proximal coxa, longer than wide in proximal-distal axis, 2.7x (Fig. 12D); medially drawn out into gnathal edge, differentiated as molar surface and acute incisor; distal palp with 3 articles, palp articles longer than wide, article 1, 1.7x; article 2, 2.2x; article 3, 2.0x, with 5 setae. Maxillula elongate without subdivision; longer than wide, 10.1x; at the tip, original median edge, with 4 robust setae (Fig. 12E). Maxilla with 2 distinct lobes; longer than wide 2.4x (Fig. 12F); medial lobe 0.03 mm wide, without setae; lateral lobe 0.04 mm wide, without setae. Maxilliped with 3 articles (Fig. 12G); without endite, without oostegite lobe (epipod); articles longer than wide, article 1 (ischium), 2.1x; article 2 (merus), 1.1x; article 3 (carpus), 2.1x. Posterior thorax appendages 6 pairs, each with 7 articles, coxae not dissected. Distal 6 articles forming functional leg (Figs. 13A–13D). Pereopod 1 (Fig. 13B) basipod longer than wide, 2.2x; ischium longer than wide, 0.8x; merus longer than wide, 0.9x; carpus longer than wide, 0.5x; propodus longer than wide, 1.7x; dactylus longer than wide, 3.5x; entire appendage without setae. Pereopod 4 (Fig. 13C) basipod longer than wide, 2.0x; ischium longer than wide, 1.5x; merus longer than wide, 0.5x; carpus longer than wide, 0.6x; propodus longer than wide, 1.6x; dactylus longer than wide, 2.6x; entire appendage without setae. Pereopod 6 (Fig. 13D) basipod longer than wide, 1.8x; ischium longer than wide, 1.5x; merus longer than wide, 0.7x; carpus longer than wide, 0.7x; propodus longer than wide, 1.7x; dactylus longer than wide, 2.9x; entire appendage without setae.

Remarks

*Ceratothoa* is a group of mouth cavity inhabiting crustaceans. All mouth cavity inhabiting representatives of Cymothoidae are positioned with their anterior end (cephalon, cephalothorax or functional head) towards the mouth opening of the fish host. An elongated, cylindrical body shape is characteristic for such parasites, as their bodies develop to occupy the round shape of the mouth of the fish host.

The specimens examined and photographed here, seem to be representatives of a species of *Ceratothoa*, currently being formally described as a new species (K.A. Hadfield, 2019, personal communication with the first author of that submitted publication) using material from South Africa.

Specialised structure variation

Besides the obvious overall size variation between male and female specimens, other ontogenetic variations were noted. The adult female has a visibly larger length to width ratio of the antennulae, and antennae, maxilla and maxilliped. Mandibles show an increase in the number of proximal setae on palp article 2. Mandible palp article 3 and maxilliped article 3 both lost the terminating setae seen in earlier stages. The number of robust setae on both the medial and lateral lobes of maxillae doubled. The maxilliped of the gravid female specimen has an oostegite lobe and endite, which are both absent in the maxilliped of the male. With regards to the pereopods, the overall shapes of all investigated ones remained similar through development, except for the size of the basal carinae and the medial bulbous protrusion on all merus articles, which are all respectively larger on pereopods of females. Pereopod 6 and 7 of females have the largest carinae, with the smallest carina on pereopod 4. Pereopod 6 and 7 of males have smaller carinae than that of pereopod 4.

The immature stage 2 of *Ceratothoa* sp. provided some remarkable morphological deviations compared to that of the female and male specimens. Some of its characters differ notably from the character states used as diagnostic for a female specimen of this species. These characters include: antennula slightly shorter than antenna; antennulae peduncle articles 1 not in contact; antennulae and antennae with numerous setae; mandible palp article 3 longer than article 2, with long, spine-like setae; maxilla smaller length to width ratio, maxilla lobes with only 1 robust seta each; maxilliped article 1 without lateral protrusion; pereopod 1 dactylus with marginal spines; pereopods 6–7 merus lateral margin with spine-like seta; pereopod 7 with spine-like setae on carpus and propodus lateral margins. These differences show the extent to which immature forms can differ from mature forms, especially regarding specialised structures, such as the mouthparts and pereopods.

The identification of the herein examined *C. gaudichaudii* (*species inquirenda*) immature stage 1 specimen, is based on: (1) The collection location of the specimen (Peru). According to Brusca (1981), there are only two species of *Ceratothoa* found in the eastern Pacific, *C. gaudichaudii* and *Ceratothoa gilberti*. Of these two species, *C. gilberti* is almost only found in the Gulf of California area, while *C. gaudichaudii* is distributed along the coast from California to the southern most parts of South America including the Galapagos Islands (Richardson, 1901; Richardson, 1910; Van Name, 1924). (2) The adult forms of the latter two species were compared to the adult forms from which the herein identified *C. gaudichaudii* was retrieved (in same collection, not illustrated) using the descriptions and identification key provided by Schioedte & Meinert (1884), Brusca (1981) and Richardson (1910). (3) The illustrations and short description of the immature forms of *C. gaudichaudii*, provided by Schioedte & Meinert (1884), Van Name (1924) and Szidat (1966), were compared to the herein examined immature. *Ceratothoa gaudichaudii* can be distinguished by having carinae on pereopods 3–6; large tergite on thorax segment 2 (first free tergite) with antero-lateral projections; pleon segment 5 posterior margin tri-sinuate; uropods extending just past pleotelson posterior margin (Brusca, 1981; Hadfield, 2012). The validity of this species has been drawn into question, as many of its morphological characteristics, including these from its ‘diagnosis’, are plesiomorpic for *Ceratotoa*. Many specimens of this species are not distinguishable from *C. imbricata* and is therefore currently interpreted as *species inquirenda* (Martin, Bruce & Nowak, 2015). A full revision is needed to resolve its status. Martin, Bruce & Nowak (2015) synonymised the specimens examined by Szidat (1966), with *C. imbricata*. The adult forms of the herein examined immature stage 2, are similar to *C. imbricata*, after comparison to *C. imbricata* from Hadfield, Bruce & Smit (2014) and additional specimens from Australia (Martin, Bruce & Nowak, 2015). Since *C. imbricata* has never been recorded from the coasts of the eastern Pacific, we refrained from synonymising the herein examined immature specimen with *C. imbricata*.

The immature stage 1 specimen of *C. gaudichaudii* (K34618) was collected from the brood pouch of a gravid female from the same collection container (K34618). According to the collection label, this gravid female specimen was collected at a fish market, from the external surface of a fish host (Gadidae). This attachment and host detail is questionable, since all species of *Ceratothoa* are mouth attaching fish parasties. The reason for the presence of these individuals from the external surface on the specified fish, is most likely due to the animal abandoning its dying or already dead host, in search of another one. Even though *C. gaudichaudii* has a low host specificity (Trilles, 1972; Brusca, 1981; Hadfield, 2012), they have not been recorded from species of the fish group Gadidae, making the host identification questionable. The movement of these animals, in search of another fish host, might explain the recording of Gadidae as the fish from which it was collected.

*Ceratothoa* currently consist of 26 known and accepted species, with 5 additional species that are in need of revision (Martin, Bruce & Nowak, 2015; Boyko et al., 2019 *onwards*). Of these 31 species, only 6 species have a partially described or illustrated immature stage (*Ceratothoa italica* Schioedte & Meinert, 1883; *Ceratothoa oestroides* (Risso, 1816); *Ceratothoa oxyrrhynchaena* Koelbel,1878; *Ceratothoa parallela* (Otto, 1828); and *Ceratothoa trigonocephala* (Leach, 1818). These descriptions and single dorsal illustrations were made by Schioedte & Meinert (1884), after which, the characterisation of immature forms became scarce. The only other instances of *Ceratothoa* immatures stages from literature are limited to the dorsal and/or ventral photographs of *C. gaudichaudii* (see Szidat, 1966), *C. parallela* (see Papapanagiotou & Trilles, 2001), *C. oestroides* (see Mladineo, 2003).

Immature inter-specific variation

The examined *C. gaudichaudii* immature stage 1 specimen was compared to the dorsal illustrations of ‘pre-manca’ (immature stage 1) and ‘manca’ (immature stage 2) individuals of *C. gaudichaudii*, by Schioedte & Meinert (1884), Van Name (1924) and Szidat (1966). The immature stage 1 individual from Schioedte & Meinert (1884) shows little variation to the one examined here. Differences are the larger size of the latter, as well as a longer pleotelson and shorter relative uropods. The immature stage 2 specimen from Van Name (1924) was an unconfirmed identification, labelled as ‘probably’ *C. gaudichaudii* due to its collection location. Even so, the latter specimen best resembles that of the immature stage 1 examined here, with the most similar characters. The only variation, is that the Van Name (1924) specimen has uropod setae and pleotelson setae, leading to the assumption that this is an immature in stage 3. Setae are absent in the herein examined specimen. The immature stage 1 and immature stage 2 individuals illustrated by Szidat (1966) varies from the immature examined here, by having a semi-circular head without a dorsally visible rostrum; smaller, more oval eyes; and shorter pleotelson and uropodal rami.

The examined immature stage 1 was further compared to the remaining 5 species of *Ceratothoa* of which there are descriptions for immature life stages. These comparisons are based on structures that can be observed and compared from the illustrations and photographs that were provided. The immature stage 2 of *C. italica* Schioedte & Meinert, 1883, differs from the examined immature in the following regards: *C. italica* has oval eyes, instead of round eyes (with straight antero-lateral margin); it is widest at thorax segment 3, whereas the examined immature is widest at thorax segment 5; pereopod 1 dactylus with lateral serrations; a shorter pleon relative to total body length; pleotelson wider than long; broader uropod rami. The immature of *C. gaudichaudii* shows the following differences to *C. oestroides* (Risso, 1816), illustrated by Schioedte & Meinert (1884): the latter species has sharper rostrum anterior margin; oval eyes, instead of round; thorax segment 2 anterior margin with medial indent; serrations on pereopod 1 dactylus; uropods extending further beyond the pleotelson posterior margin. This specimen corresponds morphologically to the immature stage 2 individual photographed by Mladineo (2003) with the exception of the latter having blunter rostrum anterior margin; larger eyes; and shorter uropods, extending only slightly past the pleotelson posterior margin. The immature stage 1 of *Ceratothoa oxyrrhynchaena* Koelbel, 1878, partly described and illustrated by Schioedte & Meinert (1884) has a narrower, sharper rostrum; eyes smaller; much longer antennae, extending to the posterior end of thorax segment 8; free thorax segments sub-equal in length, versus thorax segment 2 longest in *C. gaudichaudii*; uropods with setae, endopod longer than exopod; triangular pleotelson with pointed posterior margins. The immature stage 2 of *C. parallela* (Otto, 1828) (from Schioedte & Meinert (1884)) differs from the examined immature by: rostrum anterior margin rounded; antenna with numerous setae; pereopod 1 dactylus with serrations; longer uropods. The immature stage 2 specimen photographed by Papapanagiotou & Trilles (2001) is similar to that of Schioedte & Meinert (1884), but with a shorter thorax segment 2 and pleon appendages equal in width, versus the progressively narrower pleon segments from the latter specimen; and shorter uropods. The illustrated immature of *C. trigonocephala* (Leach, 1818) has a pointed rostrum anterior margin; shorter pleotelson (shorter than wide); setae on the pleotelson posterior margin and uropods; uropods extending further beyond the pleotelson posterior margin; and pereopod 1 dactylus with serrations.

### *Elthusa* Schioedte & Meinert, 1884

Refer to Van der Wal, Smit & Hadfield (2019) for synonymy.

Type species

*Elthusa emarginata* (Bleeker, 1857), originally described as *Livoneca emarginata* Bleeker, 1857; by monotypy (Schioedte & Meinert, 1884). The original number of type specimens that were available to Bleeker (1857) is unknown. A single female syntype, examined by Bleeker (1857), is deposited at the Naturalis Biodiversity Center (previously the Rijksmuseum von Natuurlijke Historie), Leiden (RMNH.CRUS.I.66). Another type specimen from the latter museum has been lost. The specimen examined by Schioedte & Meinert (1884) is held at the Natural History Museum in Paris (MNHN241) (Trilles, 1976).

### *Elthusa vulgaris* (Stimpson, 1857)

Type material

Originally described as *Lironeca vulgaris* Stimpson, 1857 (Smithsonian National Museum of Natural History, USNM number: 20354, Accession number: 014668), syntypes (2) *Lironeca panamnnensis* Schioedte & Meinert, 1884, (Museum of Comparative Zoology, Harvard University, Cambridge, Massachusetts, MCZ 1077) and syntypes (3) *Lironeca vulgaris* (British Museum (Natural History), London, BMNH 1878: 8), loc: San Francisco market (Ellis, 1981), as junior synonyms of *Elthusa vulgaris* (see Brusca, 1978a; Brusca, 1981).

Material examined

Three specimens from different ontogenetic stages were examined. K26553a gravid female (25.0 mm total length, 17.0 mm wide); K26553b male (15.5 mm total length, 9.0 mm wide); K26553c embryo (1.9 mm total length, 1.1 mm wide); loc: Champerico, Guatemala (Paessler); date: 23.10.1893; additionally on the label: ‘pars.s.Katal’.

Gravid female

**Figure 14:**
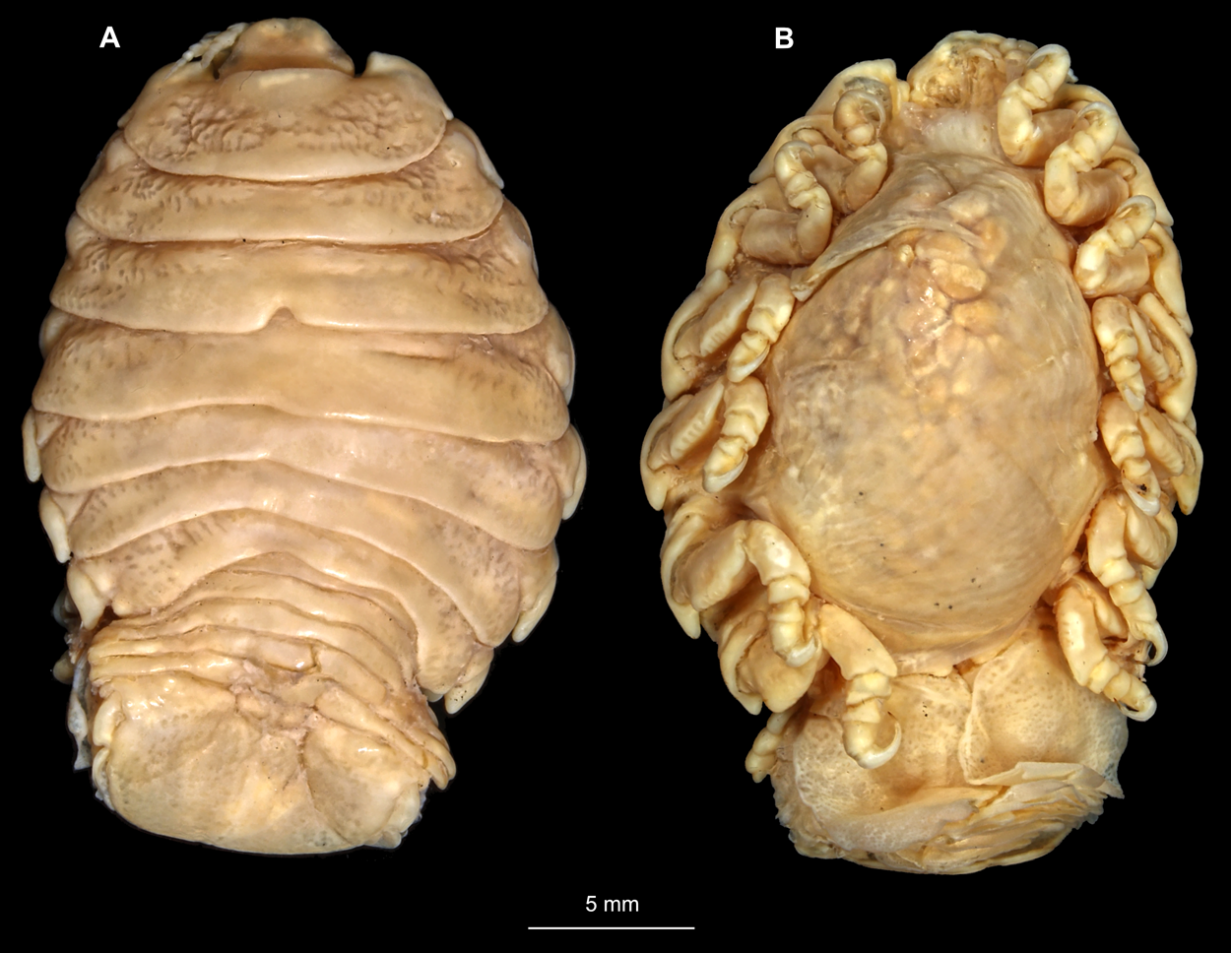
*Elhusa vulgaris* (Stimpson, 1857) gravid female (K26553a). (A) Dorsal view. (B) Ventral view.

**Figure 15:**
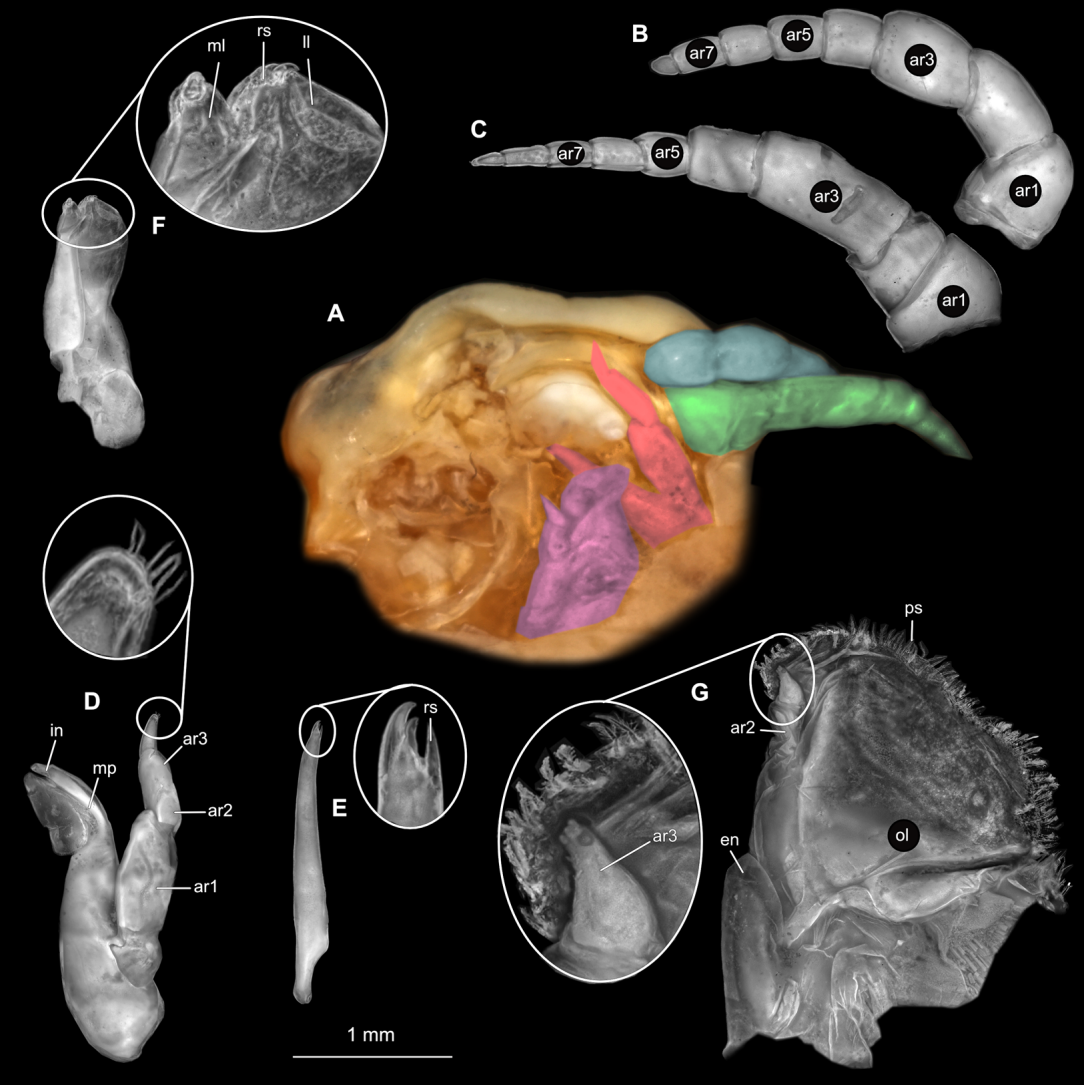
*Elhusa vulgaris* (Stimpson, 1857) gravid female (K26553a). (A) Ventral view of functional head with mouthpart positioning. (B) Antennula. (C) Antenna. (D) Mandible. (E) Maxillula. (F) Maxilla. (G) Maxilliped. ar1, article 1; ar2, article 2; ar3, article 3; ar5, article 5; ar7, article 7; mp, molar process; in, incisor; rs, robust setae; ml, medial lobe; ll, lateral lobe; ol, oostegital lobe; ps, plimose setae; en, endite.

**Figure 16:**
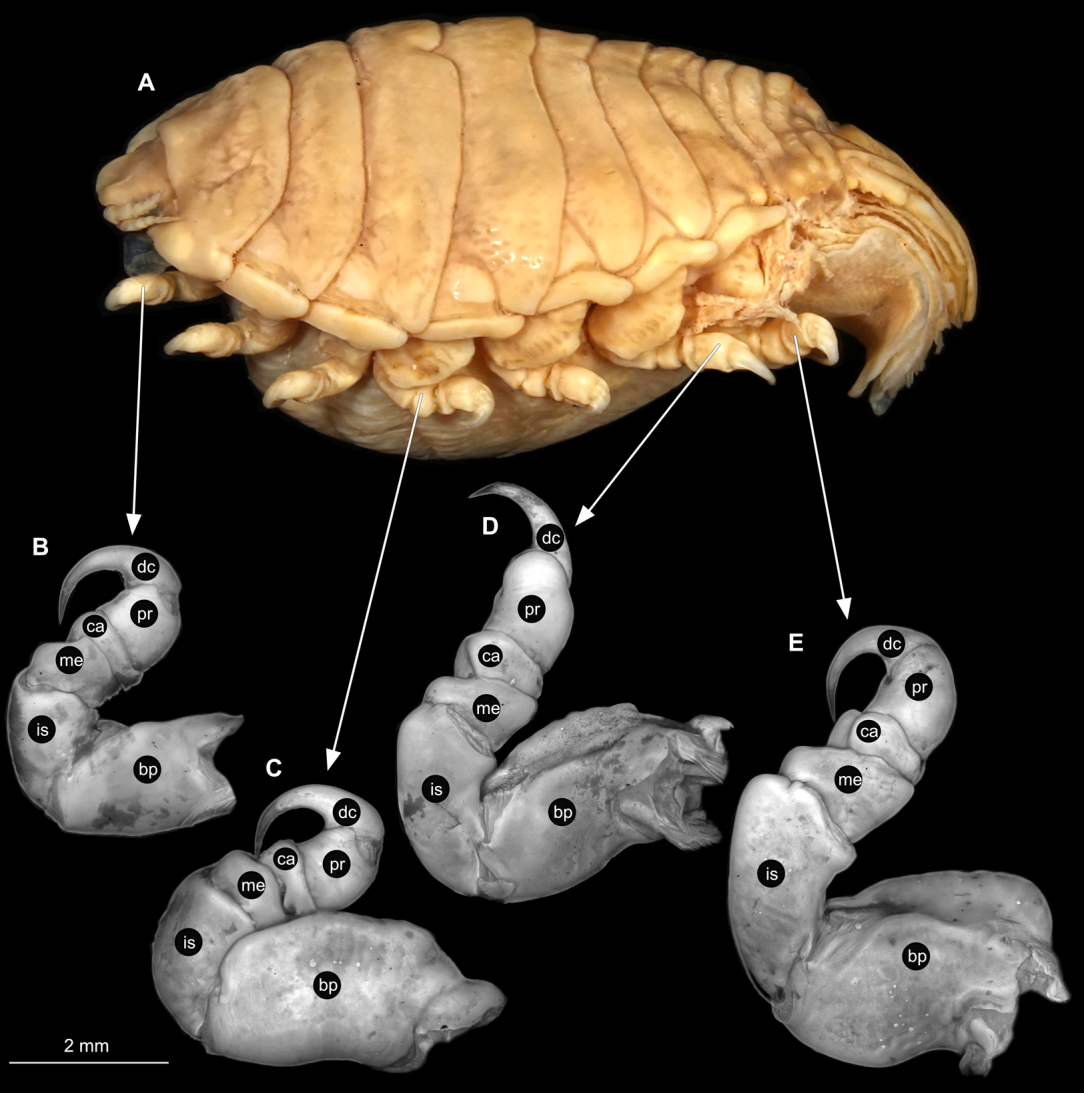
*Elhusa vulgaris* (Stimpson, 1857) gravid female (K26553a). (A) Lateral view. (B) Pereopod 1. (C) Pereopod 4. (D) Pereopod 6. (E) Pereopod 7. bp, basipod; is, ischium; me, merus; ca, carpus; pr, propodus; dc, dactylus.

Body longer than wide, 1.5x (Figs. 14A and 14B). Antennula 3.20 mm long, consists of 8 articles; articles 1 & 2 distinct, without setae (Fig. 15B); articles longer than wide, article 1, 1.2x; article 2, 1.6x; article 3, 1.2x; article 4, 1.0x. Antenna 3.53 mm long, consists of 9 articles; longer than antennula, same width as antennula; with 1 seta (Fig. 15C); articles longer than wide, article 1, 0.7x; article 2, 0.7x; article 3, 2.0x; article 4, 1.1x. Mandible with proximal coxa, longer than wide in proximal-distal axis, 3.3x (Fig. 15D); medially drawn out into gnathal edge, differentiated as molar surface and acute incisor; distal palp with 3 articles, palp articles longer than wide, article 1, 2.5x; article 2, 2.2x, with 6 setae; article 3, 2.5x. Maxillula elongate without subdivision; maxillula longer than wide, 7.8x; at the tip, original median edge, with 4 robust setae (Fig. 15E). Maxilla with 2 distinct lobes; longer than wide, 3.0x (Fig. 15F); medial lobe 0.14 mm wide, with 1 seta; lateral lobe 0.36 mm wide, with 3 setae. Maxilliped with 3 article (Fig. 15G); with medial endite, longer than wide in proximal-distal axis, 3.2x; with lobe-like oostegite, lined with multiple plumose setae; articles longer than wide; article 1 (ischium) margins difficult to discern; article 2 (merus), 1.7x; article 3 (carpus), 1.8x, with 3 robust setae. Posterior thorax appendages 7 pairs, each with 7 articles, coxae not dissected. Distal 6 articles forming functional leg (Figs. 16A–16E). Pereopod 1 (Fig. 16B) basipod longer than wide, 1.7x; ischium longer than wide, 1.6x; merus as long as wide, carpus longer than wide, 0.5x; propodus longer than wide, 1.2x; dactylus longer than wide, 2.8x; entire appendage without setae. Pereopod 4 (Fig. 16C) basipod longer than wide, 1.9x; ischium longer than wide, 2.2x; merus longer than wide, 0.6x; carpus longer than wide, 0.5x; propodus longer than wide, 0.9x; dactylus longer than wide, 2.8x; entire appendage without setae. Pereopod 6 (Fig. 16D) basipod longer than wide, 1.7x; ischium longer than wide, 2.0x; merus longer than wide, 0.9x; carpus longer than wide, 0.6x; propodus longer than wide, 1.3x; dactylus longer than wide, 2.5x; entire appendage without setae. Pereopod 7 (Fig. 16E) basipod longer than wide, 1.3x; ischium longer than wide, 1.8x; merus longer than wide, 0.7x; carpus longer than wide, 0.5x; propodus longer than wide, 1.4x; dactylus longer than wide, 2.8x; entire appendage without setae.

Male

**Figure 17:**
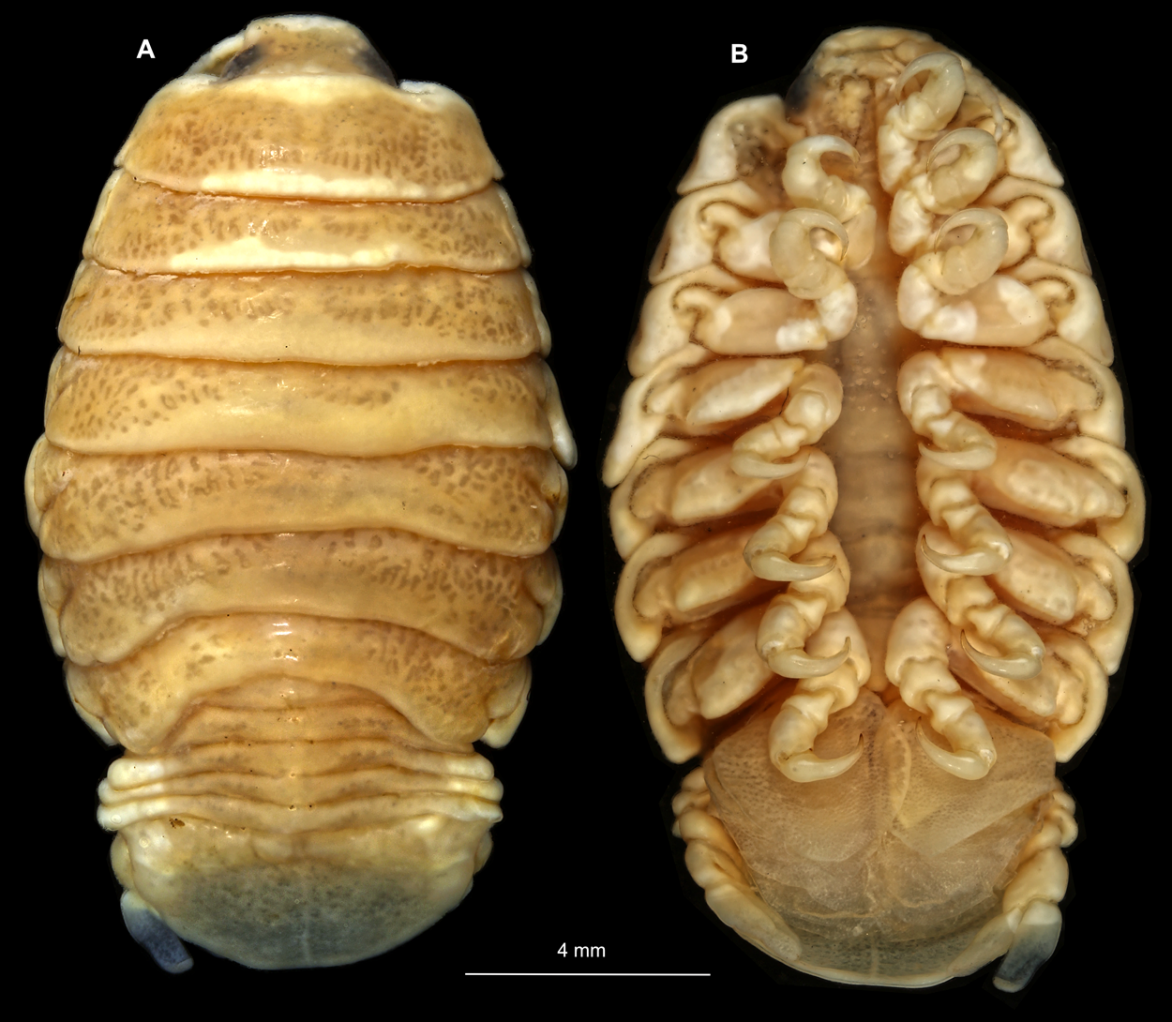
*Elhusa vulgaris* (Stimpson, 1857) male (K26553b). (A) Dorsal view. (B) Ventral view.

**Figure 18:**
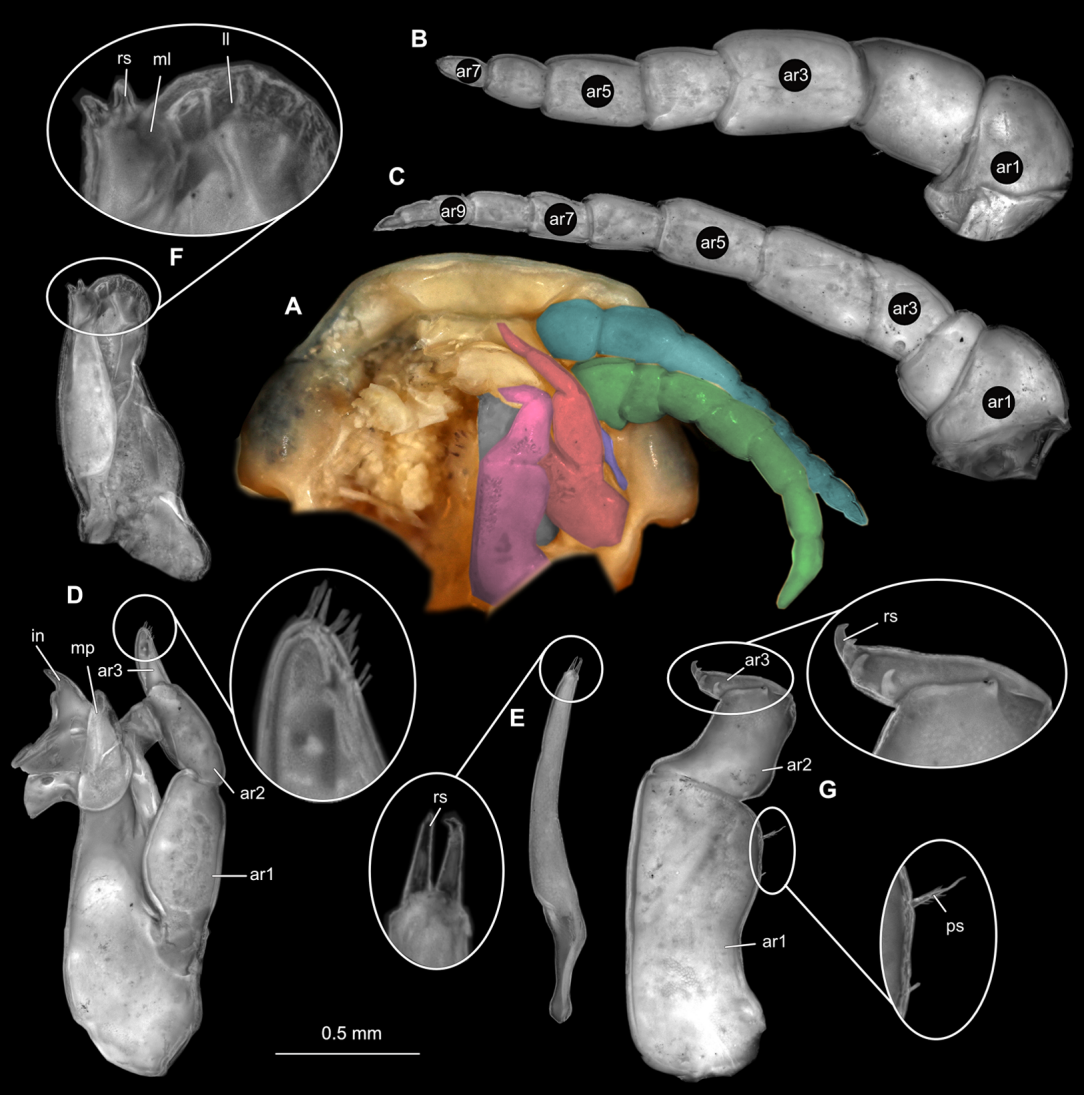
*Elhusa vulgaris* (Stimpson, 1857) male (K26553b). (A) Ventral view of functional head with mouthpart positioning. (B) Antennula. (C) Antenna. (D) Mandible. (E) Maxillula. (F) Maxilla. (G) Maxilliped. ar1, article 1; ar2, article 2; ar3, article 3; ar5, article 5; ar7, article 7; ar9, article 9; mp, molar process; in, incisor; rs, robust setae; ml, medial lobe; ll, lateral lobe; ol, oostegital lobe; ps, plimose setae.

**Figure 19:**
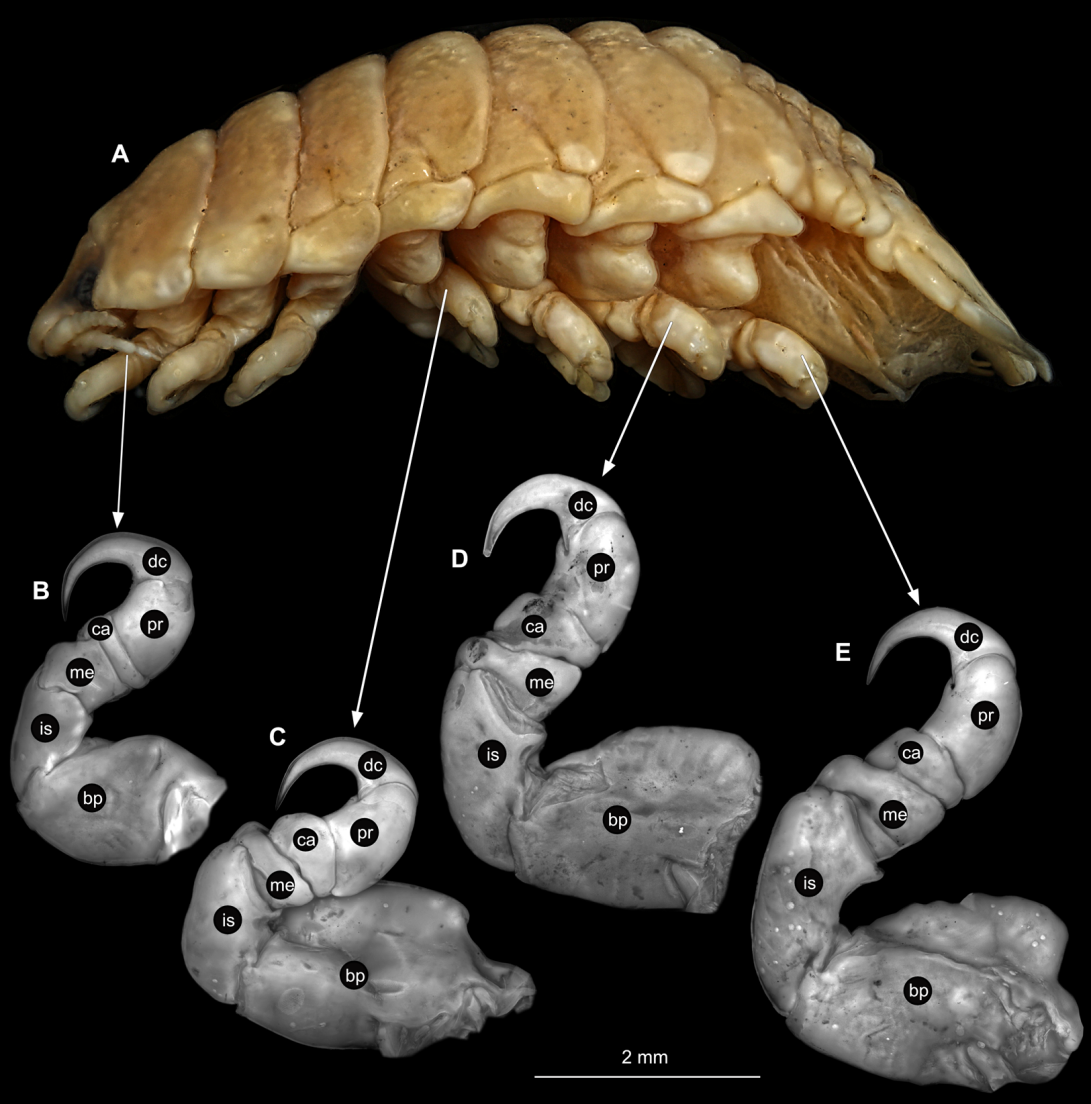
*Elhusa vulgaris* (Stimpson, 1857) male (K26553b). (A) Lateral view. (B) Pereopod 1. (C) Pereopod 4. (D) Pereopod 6. (E) Pereopod 7. bp, basipod; is, ischium; me, merus; ca, carpus; pr, propodus; dc, dactylus.

Body longer than wide, 1.7x (Figs. 17A and 17B). Antennula 2.31 mm long, consists of 7 articles, articles 1 & 2 distinct with 1 plumose seta on article 2, 2 setae on article 6 and 4 setae on terminating article (Fig. 18B); articles longer than wide, article 1, 0.7x; article 2, 1.2x; article 3, 1.3x; article 4, 1.1x. Antenna 2.51 mm long, consists of 11 articles; longer than antennula; thinner than antennula (Fig. 18C); with 6 setae on terminating article; articles longer than wide, article 1, 0.8x; article 2, 0.6x.; article 3, 1.1x; article 4, 1.5x. Mandible with proximal coxa, longer than wide in proximal-distal axis, 3.0x (Fig. 18D); medially drawn out into gnathal edge, differentiated as molar surface and acute incisor; distal palp with 3 articles, palp articles longer than wide, article 1, 2.2x; article 2, 2.3x; article 3, 2.2x, with 11 setae. Maxillula elongate without subdivision; longer than wide, 1.3x, at the tip, original median edge, with 2 robust setae (Fig. 18E). Maxilla with 2 partly conjoined lobes; longer than wide, 2.6x (Fig. 18F); medial lobe 0.10 mm wide, with 2 setae; lateral lobe 0.21 mm wide, without setae. Maxilliped with 3 articles (Fig. 18G); without endite, without oostegite lobe; articles longer than wide; article 1 (ischium), 1.8x; article 2 (merus), 1.1x, with 2 setae; article 3 (carpus), 1.5x, with 4 robust setae. Posterior thorax appendages 7 pairs, each with 7 articles, coxae not dissected (Figs. 19A–19E). Distal 6 articles forming functional leg. Pereopod 1 (Fig. 18B) basipod longer than wide, 1.3x; ischium longer than wide, 1.6x; merus longer than wide, 0.9x; carpus longer than wide, 0.5x; propodus longer than wide, 1.3x; dactylus longer than wide, 3.3x; entire appendage without setae. Pereopod 4 (Fig. 18C) basipod longer than wide, 1.5x, ischium longer than wide, 1.9x; merus longer than wide, 0.5x; carpus longer than wide, 0.6x; propodus longer than wide, 1.6x; dactylus longer than wide, 2.8x; entire appendage without setae. Pereopod 6 (Fig. 18D) basipod longer than wide, 1.5x; ischium longer than wide, 1.7x; merus longer than wide, 0.5x; carpus longer than wide, 0.6x; propodus longer than wide, 1.5x; dactylus longer than wide, 2.8x; entire appendage without setae. Pereopod 7 (Fig. 18E) basipod longer than wide, 1.4x; ischium longer than wide, 2.2x; merus longer than wide, 0.7x; carpus longer than wide, 0.7x; propodus longer than wide, 1.5x; dactylus longer than wide, 2.5x; entire appendage without setae.

Embryo

Body elongated, encapsulated in membrane, longer than wide, 1.8x (Fig. 10B). Antennula 0.33 mm long, longer than wide, 4.3x. Antenna 0.31 mm long, longer than wide, 4.7x. Maxilliped longer than wide, 3.5x. Pereopod 1 longer than wide, 4.3x. Pereopod 4 longer than wide, 3.9x. Pereopod 6 longer than wide, 5.4x. Eyes visible.

Remarks

In the past, there was some confusion with the original generic name of this genus. Two generic names *Livoneca* Leach, 1818 and *Lironeca* Leach, 1818 are mentioned. *Lironeca* being the generic name for the originally described type material and *Livoneca* as the generic name for the type species of *Elthusa* (see *Type species* and *Type material* sections). These names have caused confusion among authors as to which spelling is correct (Bruce, 1990). Sivertsern & Holthuis (1980) support the original spelling of *Livoneca*, as it was used in the original publication of Leach 1818. No contradictory arguments have been provided since then and *Livoneca* was accepted as the correct spelling (Bruce, 1990). In 1994, Williams & Bowman (1994) proposed *Lironeca* Leach, 1818 as the correct original spelling to the International Commision on Zoological Nomenclature (ICZN) (Case: 2915). They argued that the original spelling of *Livoneca* was as a result of a printing error. The latter authors proposed *Lironeca* be accepted as correct spelling of the genus for the sake of stability, rather than to revert to the older spelling of *Livoneca*.

*Elthusa* is a group of gill cavity inhabiting crustaceans, with the exception of two species that are known from individuals that have been found attached to the mouth cavity of its fish host. A slightly vaulted, ovoid to round body shape is characteristic to parasites attaching to this site, in order to occupy the space within the fish host gill cavity.

With the original description of the species, Stimpson (1857) gave morphological features of a females specimen with a single, dorsal view illustration. Possibly, due to the lack of equipment and technology at the time, details and characters of specialised structures such as mouthparts, and pereopods were not included. Schioedte & Meinert (1884) gave descriptions of a gravid female, male and ‘pullus 1’ (immature stage 1), including some characters of pereopods, but still lacking the mouthpart morphology. These descriptions included a dorsal and lateral view illustrations of each specimen. The first mentioning and illustration of specialised structures were provided by Richardson (1905). This included illustrations of the mandible palp and maxilliped of a female specimen, as well as the illustrations for the maxillula, maxilla, pereopod 7 and a dorsal view of a male specimen.

Brusca (1978a) provided more detailed descriptions of a non-ovigerous female, male and immature stage 3 specimens. Illustrations of the female specimen include mouthparts and pereopods 1, 5 and 7. Mouthpart morphology of the male and immature were not provided. In addition, Brusca (1978a) provided a proposed life cycle and ‘post-marsupial’ development of *E. vulgaris* with an illustration of an embryonic and immature stage 2 specimens (without description). Another female and male descriptions were made later by Brusca (1981), that included illustrations of dorsal view of a female, as well as mouthparts, pereopods and pleon segments.

*Elthusa vulgaris* is distributed mainly in the eastern Pacific ocean, with records from northern California, USA, down to the Malpelo Island, Colombia (Bennett, 1993; Brusca, 1978a, 1981; Gamble, Smith & Chi, 2013). Espinosa-Pérez & Hendrickx (2001) recorded *Elthusa vulgaris* from Puerto Mandero, Chiapas, Mexico, which is the closest location to that of the specimens examined here. Champerico, Guatemala, is thus added as a new location for *E. vulgaris*.

Specialised structure variation

The number of articles on antennulae and antennae varied between female and male specimens. The gravid female has one additional antennula article, but 2 less articles on the antenna. The overall shape of the maxilla remained similar, with the addition of 3 robust setae on the lateral lobe of the female specimen. Mandible palp article 3 setation decreased in number. Female maxillula has 4 terminal robust setae, compared to the 2 of the male specimen. The female maxilliped article 1 has the addition of an oostegite lobe (epipod) and endite attached, which is absent from the male specimen. The lateral plumose setae that are visible on the male maxilliped article 1, is absent from the female counterpart. Pereopod shapes remained rather similar through development.

The mouthpart morphology of the gravid female specimen shows little to no variation to the description and illustration of mouthparts and pereopods of a female by Richardson (1905), the presence of oostegite lobe on the illustrated maxilliped by Richardson (1905), suggests that it is also from an ovigerous female specimen. When compared to the illustrations of Brusca (1978a), the herein examined female has one additional antenna article and less setae on individual mouthparts. The overall structures are morphologically similar to that provided by Brusca (1978a) with the exception of the absence of oostegite lobe on the maxilliped examined by Brusca (1978a), suggesting that it was a non-ovigerous female specimen. The most noticeable variation between the herein examined female and that from Brusca (1978a) is seen among the pereopods, where those from the latter have more slender pereopods, smaller carinae on the basipods and much shorter dactyli. The photographed pereopods (Figs. 16A–16E) are more similar to those illustrated by Brusca (1981).

***Anilocra* Leach, 1818**

Refer to Aneesh et al. (2019a) for synonymy.

Type species

*Anilocra cuvieri* Leach, 1818, by subsequent designation (Kussakin, 1979), syntype material (BMNH 1979: 332), loc: Island of Ivica (Ibiza?), Mediterranean Sea. *Anilocra mediterranea* Leach, 1818, holotype material (BMNH 1979:330), loc: Sicily, Italy (see Ellis, 1981). Both have been synonymised with *Anilocra physodes* (BMNH 1758: 636)

***Anilocra physodes* (Linnaeus, 1758)**

Material examined

Five specimens of different ontogenetic stages were examined. K19211a gravid female (56.0 mm total length, 23.0 mm wide); K19211b non-gravid female (37.0 mm total length, 14.0 mm wide); K19211c male (17.0 mm total length, 6.5 mm wide); K19211d embryo (1.17 mm total length, 0.76 mm wide). Collected from Naples, Italy, deposited at CeNak, Hamburg. No date, host or location data was recorded. K15_4 immature (8.0 mm total length, 2.4 mm wide), collected from a Gobiidae fish species, date: 24.05.2015, deposited at ZSM.

Gravid female

**Figure 20:**
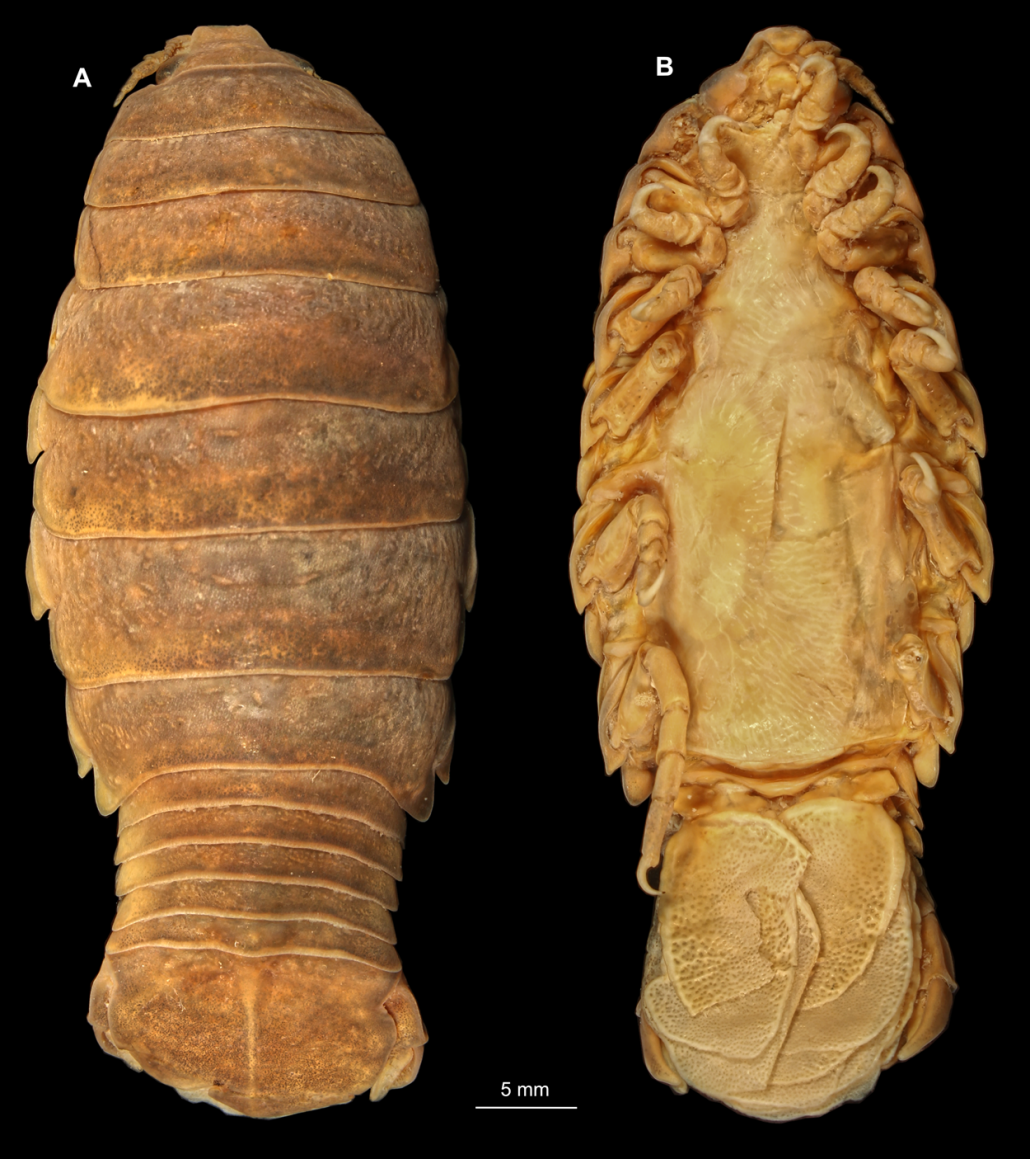
*Anilocra physodes* (Linnaeus, 1758) gravid female (K26553a). (A) Dorsal view. (B) Ventral view.

**Figure 21:**
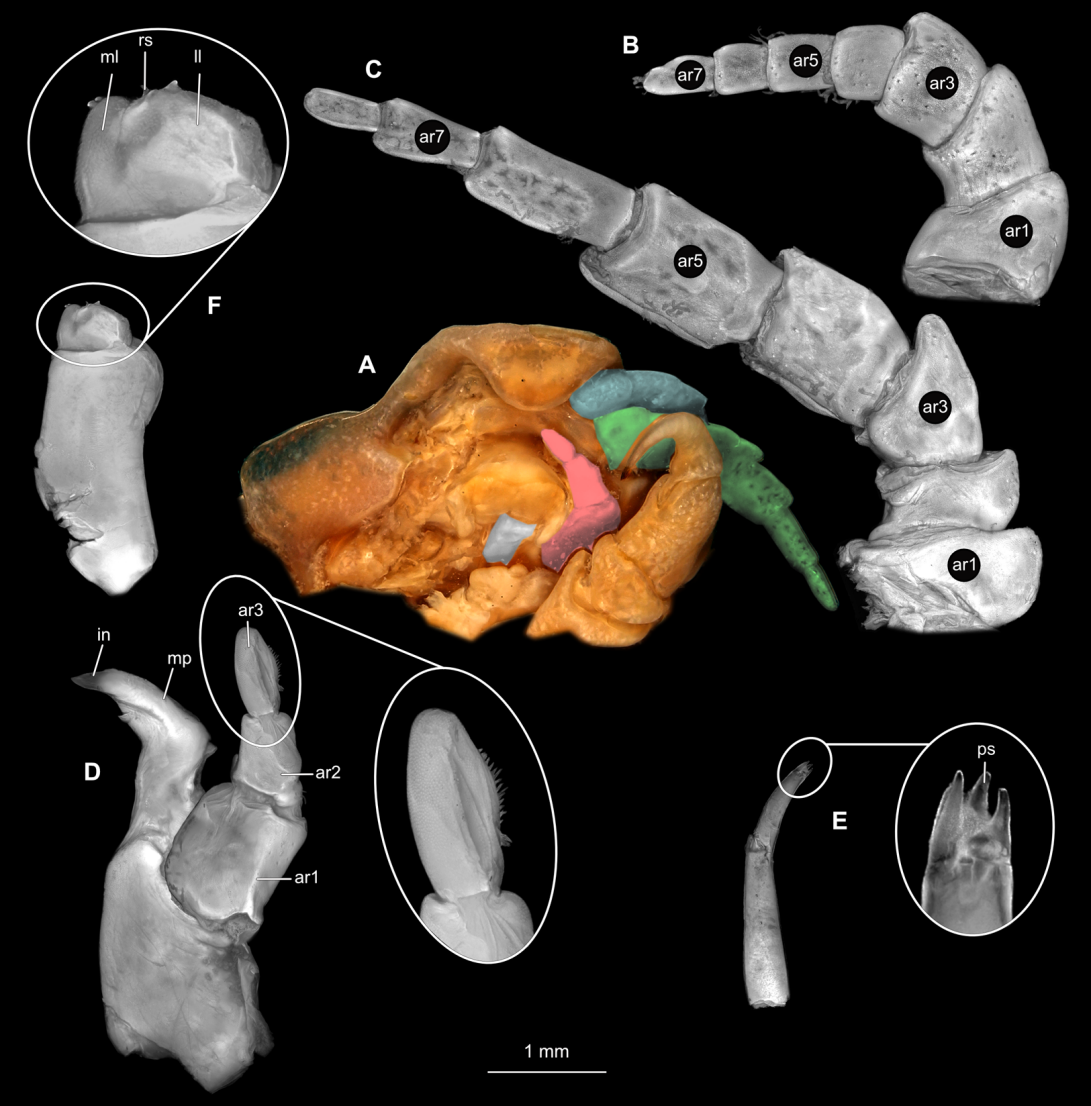
*Anilocra physodes* (Linnaeus, 1758) gravid female (K26553a). (A) Ventral view of functional head with mouthpart positioning. (B) Antennula. (C) Antenna. (D) Mandible. (E) Maxillula. (F) Maxilla. ar1, article 1; ar2, article 2; ar3, article 3; ar5, article 5; ar7, article 7; mp, molar process; in, incisor; rs, robust setae; ml, medial lobe; ll, lateral lobe.

**Figure 22:**
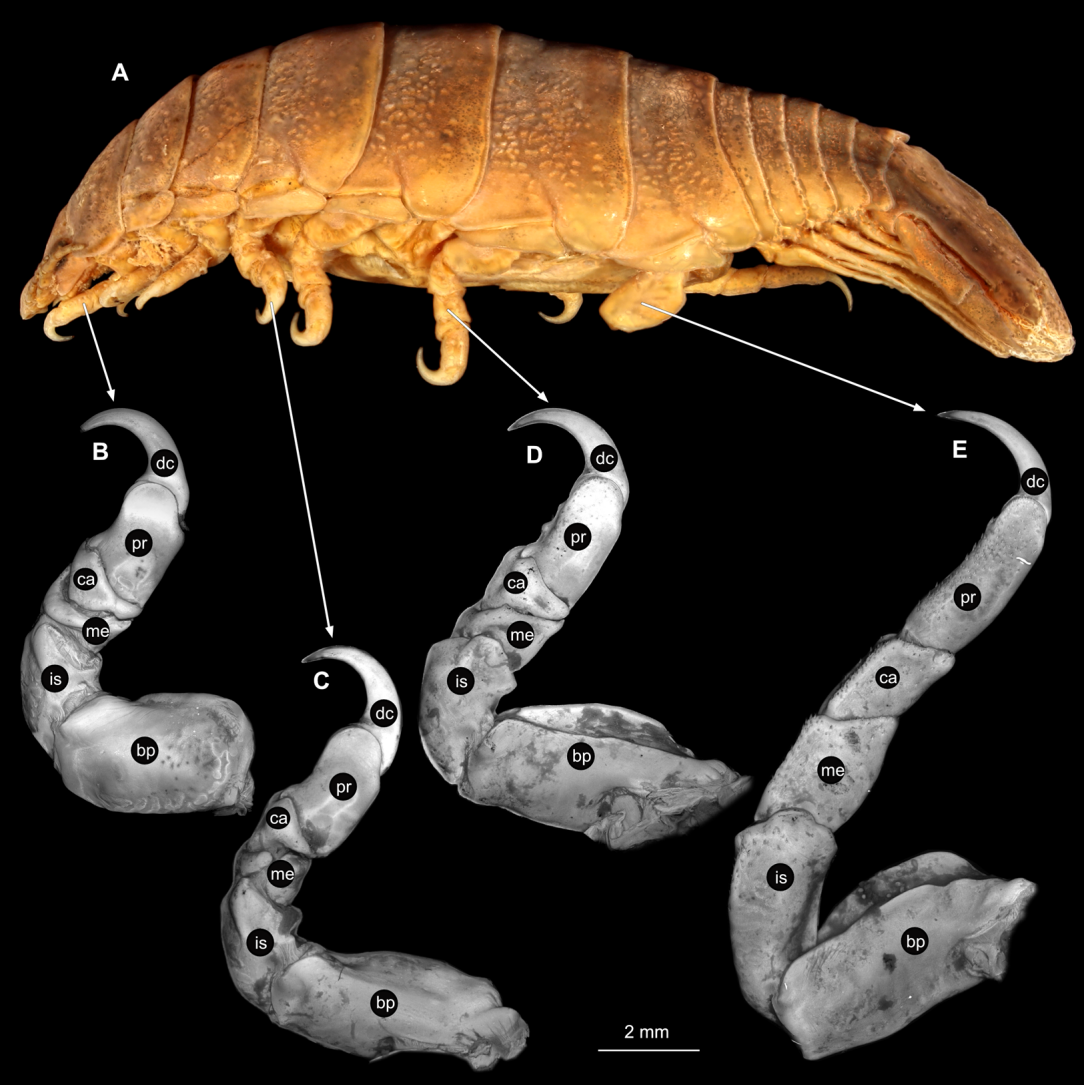
*Anilocra physodes* (Linnaeus, 1758) gravid female (K26553a). (A) Lateral view. (B) Pereopod 1. (C) Pereopod 4. (D) Pereopod 6. (E) Pereopod 7. bp, basipod; is, ischium; me, merus; ca, carpus; pr, propodus; dc, dactylus.

Body longer than wide, 2.4x (Figs. 20A and 20B). Antennula 4.59 mm long, consists of 7 articles; articles 1 & 2 distinct; with tufts of simple setae on articles 3–7 (Fig. 21B); articles longer than wide, article 1, 0.7x; article 2, 0.9x; article 3, 0.8x; article 4, 0.9x. Antenna 7.74 mm long, consists of 8 articles; longer than antennula, same width as antennula (Fig. 21C); with tufts of simple setae on articles 4–6; articles longer than wide, article 1, 0.5x; article 2, 0.5x; article 3, 1.3x.; article 4, 1.2x. Mandible with proximal coxa, longer than wide in proximal-distal axis, 2.7x; medially drawn out into gnathal edge, differentiated as molar surface and acute incisor (Fig. 21D); distal palp with 3 articles, palp articles longer than wide, article 1, 1.3x; article 2, 1.3x, with 2 setae; article 3, 2.2x, with 18 setae. Maxillula elongate without subdivision; longer than wide, 6.0x; at the tip, original median edge, with 4 robust setae (Fig. 21E). Maxilla with 2 partly conjoined lobes; longer than wide, 2.6x (Fig. 21F); medial lobe 0.12 mm wide, with 2 setae; lateral lobe 0.48 mm wide, with 2 setae. Thorax appendages 7 pairs, each with 7 articles, coxae not dissected. Distal 6 articles forming functional leg (Figs. 22A–22E). Pereopod 1 (Fig. 22B) basipod longer than wide, 1.7x; ischium longer than wide, 1.6x; merus longer than wide, 0.5x; carpus longer than wide, 0.8x; propodus longer than wide, 1.4x; dactylus longer than wide, 2.9x; entire appendage without setae. Pereopod 4 (Fig. 22C) basipod longer than wide, 2.3x; ischium longer than wide, 1.7x; merus longer than wide, 0.7x; carpus longer than wide, 0.8x; propodus longer than wide, 1.6x; dactylus longer than wide, 2.8x; entire appendage without setae. Pereopod 6 (Fig. 22D) basipod longer than wide, 2.0x; ischium longer than wide, 1.5x; merus longer than wide, 0.7x; carpus longer than wide, 0.9x; propodus longer than wide, 1.6x; dactylus longer than wide, 2.4x; entire appendage without setae. Pereopod 7 (Fig. 22E) basipod longer than wide, 2.0x; ischium longer than wide, 1.9x; merus longer than wide, 1.3x; carpus longer than wide, 1.4x; propodus longer than wide, 3.1x; dactylus longer than wide, 3.7x; appendage with numerous setae insertion areas; setae on ischium to propodus.

Non-gravid female

**Figure 23:**
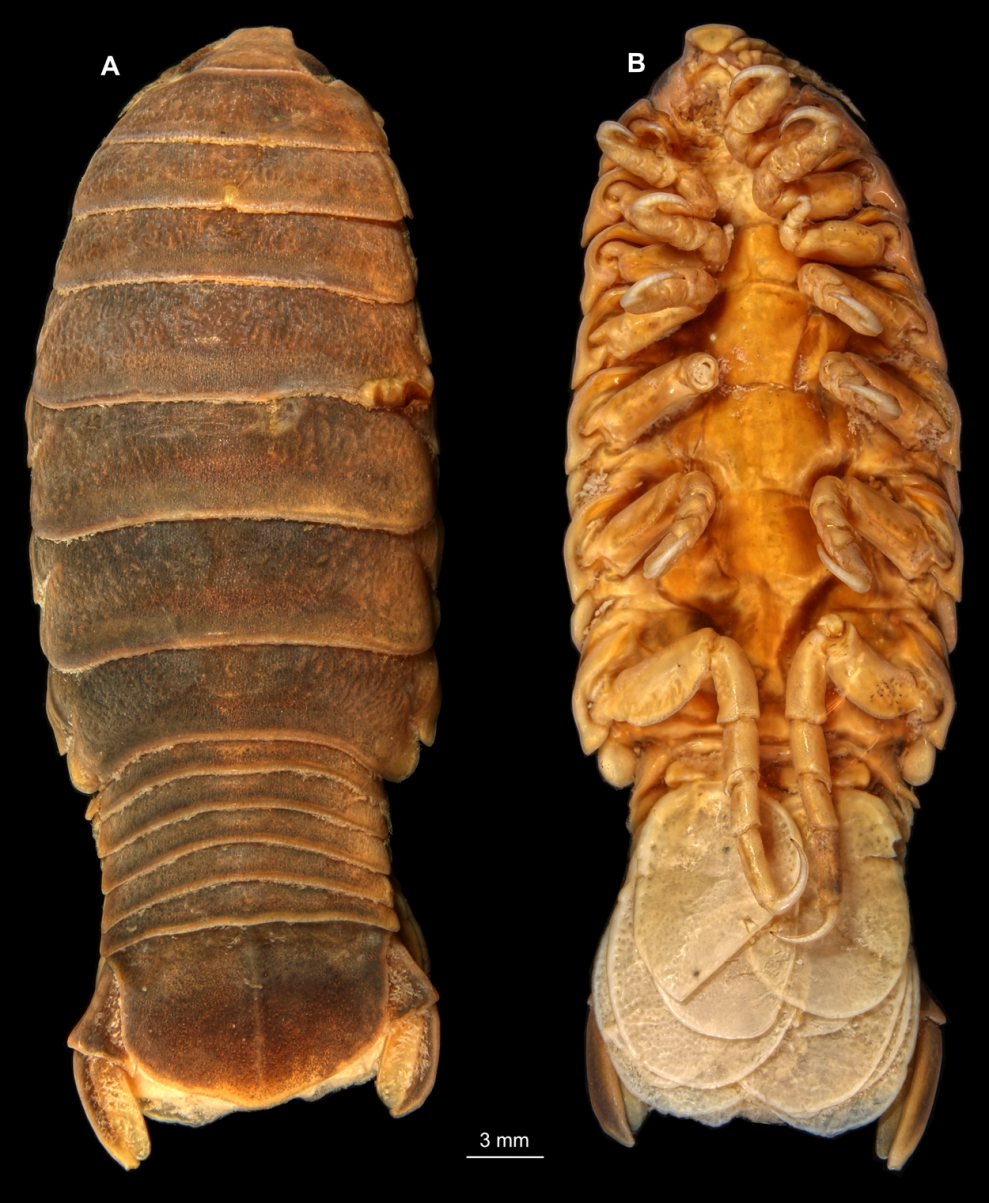
*Anilocra physodes* (Linnaeus, 1758) non-gravid female (K26553b). (A) Dorsal view. (B) Ventral view.

**Figure 24:**
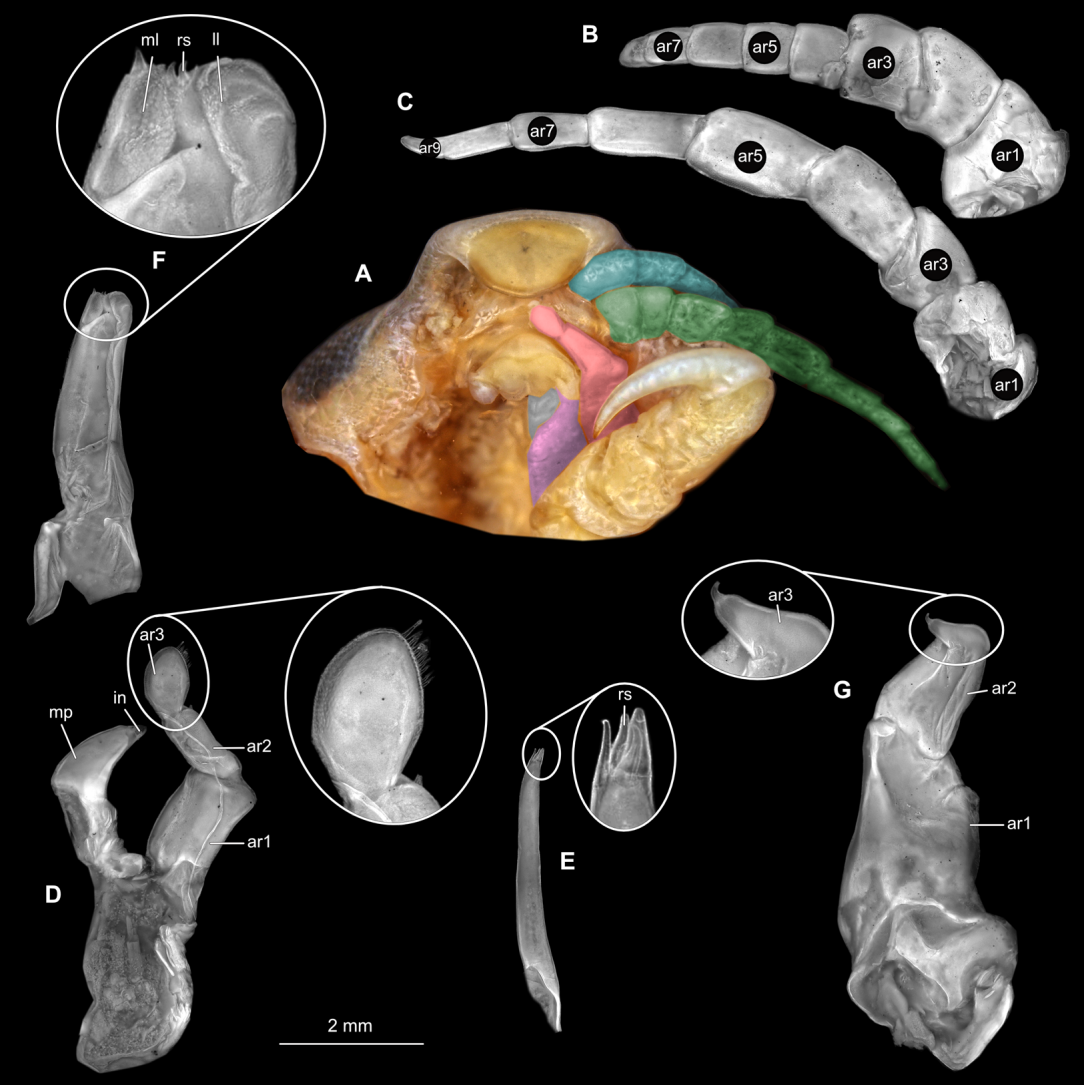
*Anilocra physodes* (Linnaeus, 1758) non-gravid female (K26553b). (A) Ventral view of functional head with mouthpart positioning. (B) Antennula. (C) Antenna. (D) Mandible. (E) Maxillula. (F) Maxilla. (G) Maxilliped. ar1, article 1; ar2, article 2; ar3, article 3; ar5, article 5; ar7, article 7; mp, molar process; in, incisor; rs, robust setae; ml, medial lobe; ll, lateral lobe.

**Figure 25:**
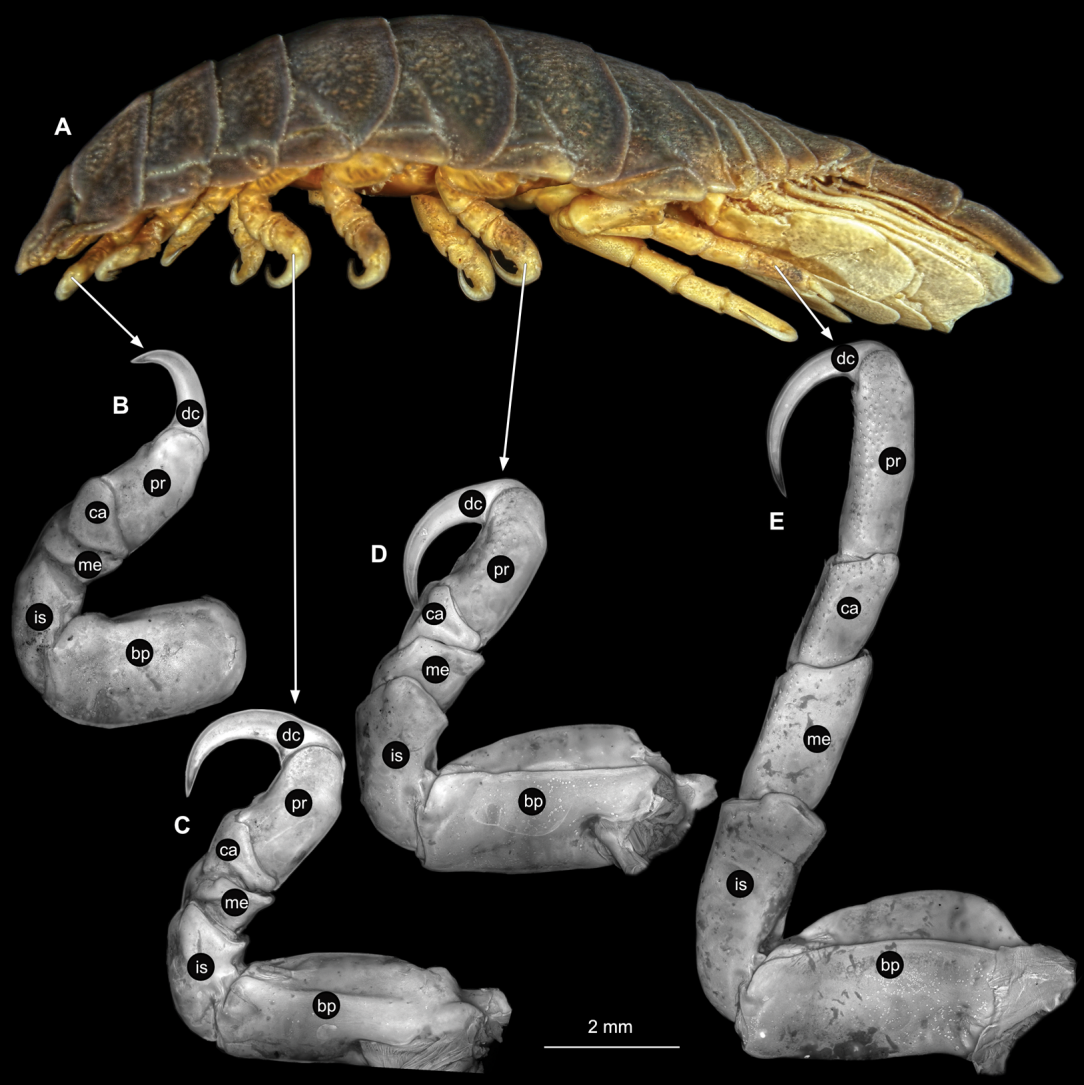
*Anilocra physodes* (Linnaeus, 1758) non-gravid female (K26553b). (A) Lateral view. (B) Pereopod 1. (C) Pereopod 4. (D) Pereopod 6. (E) Pereopod 7. bp, basipod; is, ischium; me, merus; ca, carpus; pr, propodus; dc, dactylus.

Body longer than wide, 2.6x (Figs. 23A and 23B). Antennula 3.43 mm long, consists of 8 articles; articles 1 & 2 distinct (Fig. 24B); articles longer than wide, article 1, 0.9x; article 2, 0.9x; article 3, 1.0x; article 4, 0.9x. Antenna 5.20 mm long, consists of 9 articles; longer than antennula, thinner than antennula (Fig. 24C); articles longer than wide, article 1, 0.5x; article 2, 0.8x; article 3, 0.9x; article 4, 1.6x. Mandible with proximal coxa, longer than wide in proximal-distal axis, 3.2x (Fig. 24D); medially drawn out into gnathal edge, differentiated as molar surface and acute incisor; distal palp with 3 articles, palp articles longer than wide, article 1, 2.0x; article 2, 2.2x, with 2 setae; article 3, 1.5x, with 11 setae. Maxillula elongate without subdivision (Fig. 24E); longer than wide, 9.6x; at the tip, original median edge, with 4 robust setae. Maxilla with 2 distinct lobes; longer than wide, 4.3x (Fig. 24F); medial lobe 0.13 mm wide, with 3 setae; lateral lobe 0.22 mm wide, with 3 setae; with endite, longer than wide, 3.0x. Maxilliped with 3 articles (Fig. 24G); without basal endite, without coxal oostegite lobe (epipod); articles longer than wide, article 1 (ischium), 2.2x; article 2 (merus), 1.3x, without setae; article 3 (carpus), 2.5x, with 1 robust seta. Thorax appendages 7 pairs, each with 7 articles, coxae not dissected. Distal 6 articles forming functional leg (Figs. 25A–25E). Pereopod 1 (Fig. 25B) basipod longer than wide, 1.7x; ischium longer than wide, 1.9x; merus longer than wide, 0.6x; carpus longer than wide, 0.7x; propodus longer than wide, 1.6x; dactylus longer than wide, 2.7x, entire appendage without setae. Pereopod 4 (Fig. 25C) basipod longer than wide, 2.5x; ischium longer than wide, 1.8x; merus longer than wide, 0.6x; carpus longer than wide, 0.8x; propodus longer than wide, 1.9x; dactylus longer than wide, 3.0x; appendage with numberous setae insertion areas; visible setae on carpus, propodus. Pereopod 6 (Fig. 25D) basipod longer than wide, 2.1x; ischium longer than wide, 21.9x; merus longer than wide, 0.7x; carpus longer than wide, 1.1x; propodus longer than wide, 2.0x; dactylus longer than wide, 3.0x. Pereopod 7 (Fig. 25E) basipod longer than wide, 2.0x; ischium longer than wide, 2.1x; merus longer than wide, 1.7x; carpus longer than wide, 1.8x; propodus longer than wide, 3.5x; dactylus longer than wide, 4.2x; appendage with numerous setae on ischium to propodus.

Male

**Figure 26:**
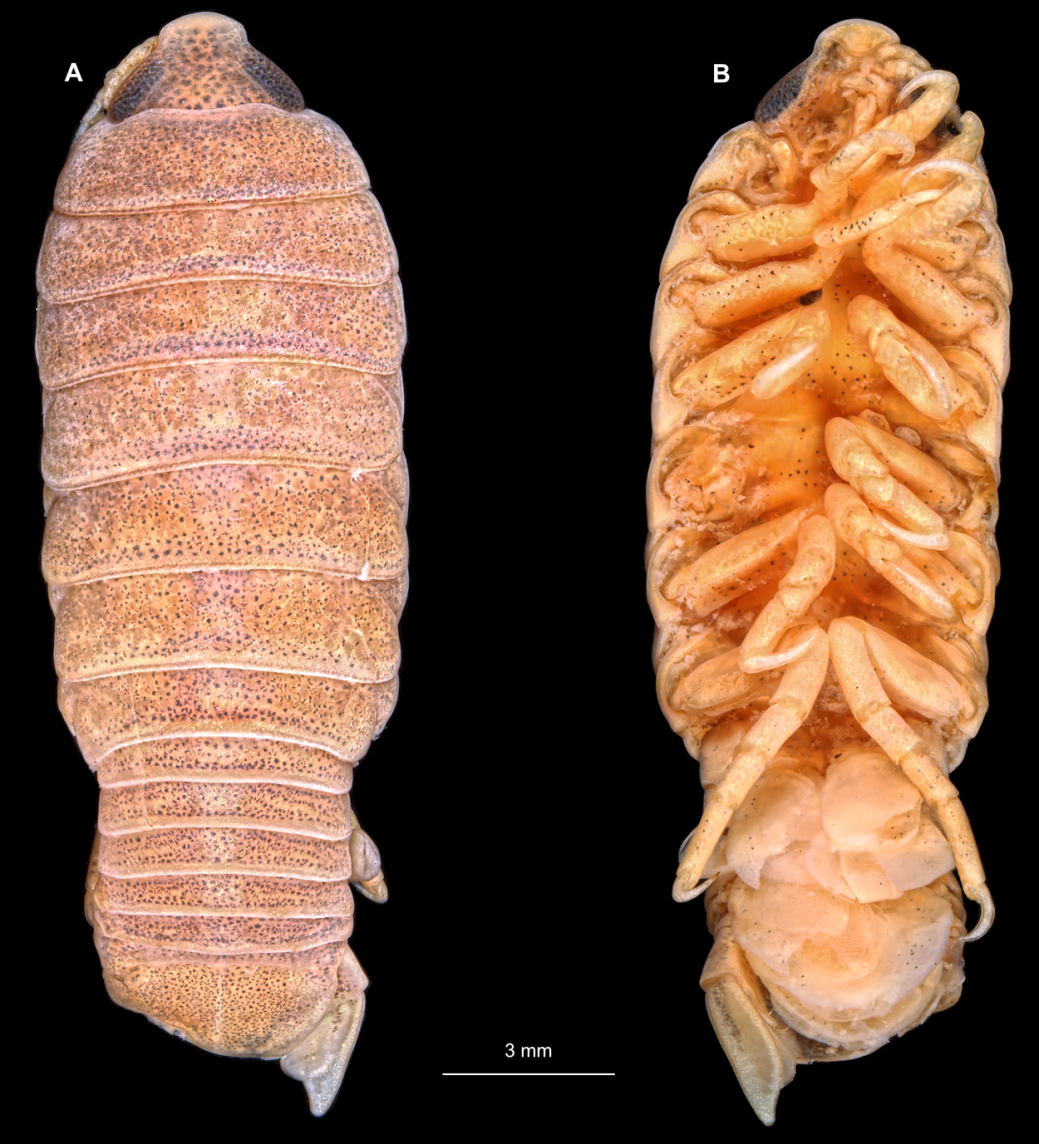
*Anilocra physodes* (Linnaeus, 1758) male (K26553c). (A) Dorsal view. (B) Ventral view.

**Figure 27:**
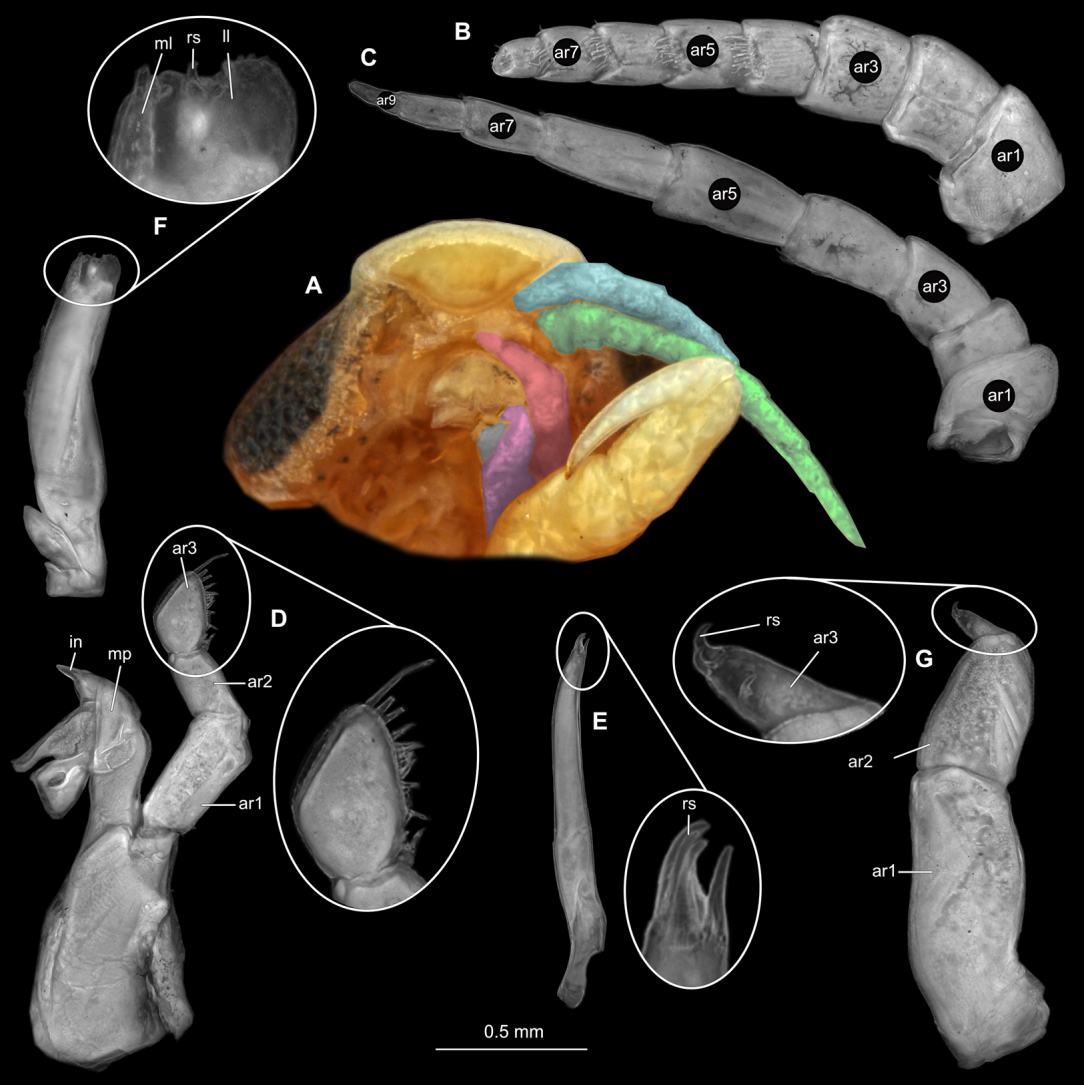
*Anilocra physodes* (Linnaeus, 1758) male (K26553c). (A) Ventral view of functional head with mouthpart positioning. (B) Antennula. (C) Antenna. (D) Mandible. (E) Maxillula. (F) Maxilla. (G) Maxilliped. ar1, article 1; ar2, article 2; ar3, article 3; ar5, article 5; ar7, article 7; mp, molar process; in, incisor; rs, robust setae; ml, medial lobe; ll, lateral lobe.

**Figure 28:**
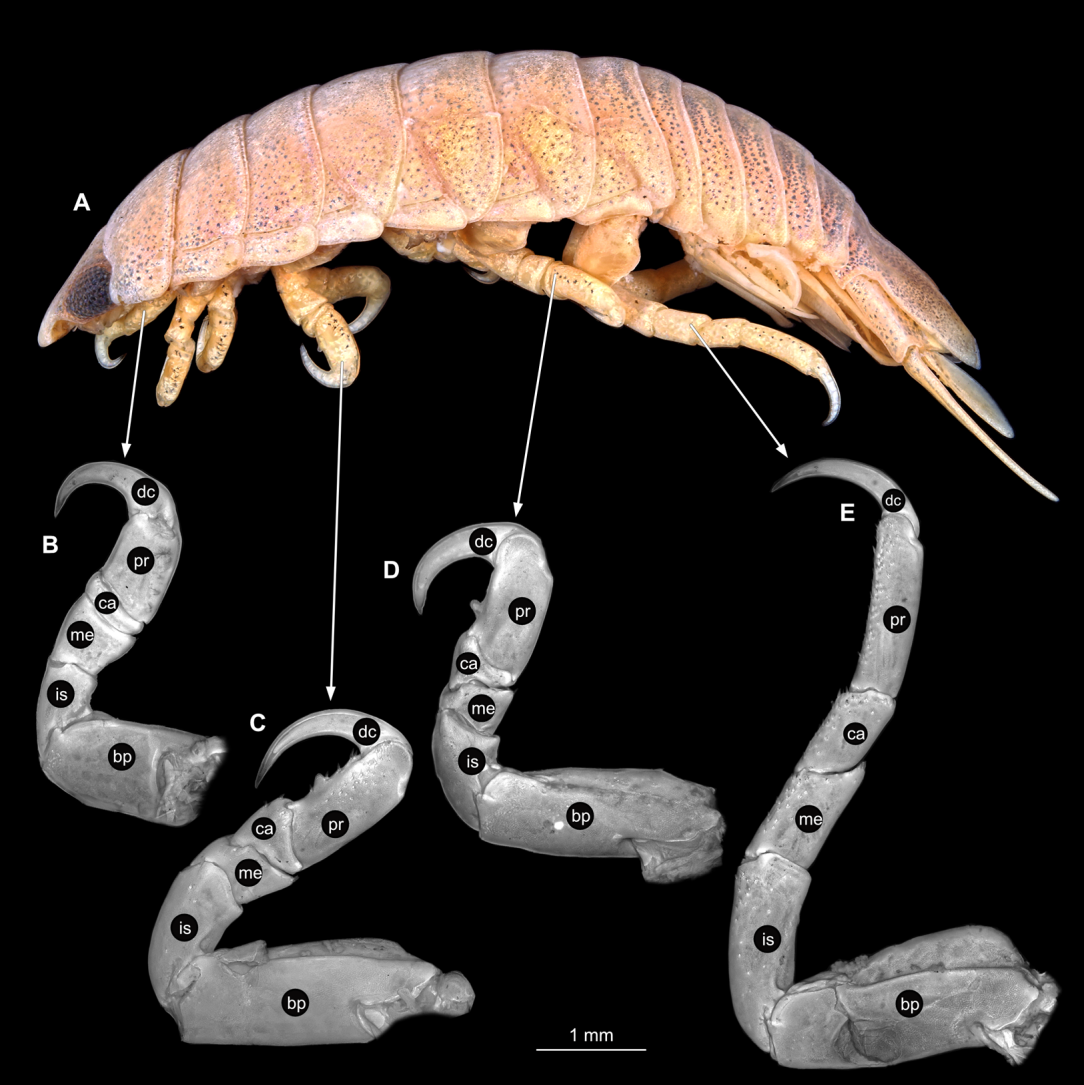
*Anilocra physodes* (Linnaeus, 1758) male (K26553c). (A) Lateral view. (B) Pereopod 1. (C) Pereopod 4. (D) Pereopod 6. (E) Pereopod 7. bp, basipod; is, ischium; me, merus; ca, carpus; pr, propodus; dc, dactylus.

Body longer than wide, 2.6x (Figs. 26A and 26B). Antennula 2.04 mm long, consists of 8 articles; articles 1 & 2 distinct; with tufts of setae on articles 4–8 (Fig. 27B); all articles longer than wide, article 1, 0.8x; article 2, 0.8x; article 3, 0.9x; article 4, 0.9x. Antenna 2.62 mm long, consists of 9 articles; longer than antennula; thinner than antennula; with simple setae on article 4, 7 (Fig. 27C); articles longer than wide, article 1, 0.6x; article 2, 0.6x; article 3, 1.3x; article 4, 1.5x. Mandible with proximal coxa, longer than wide in proximal-distal axis, 2.8x (Fig. 27D); medially drawn out into gnathal edge, differentiated as molar surface and acute incisor; distal palp with 3 articles, palp articles longer than wide; article 1, 2.1x; article 2, 2.3x, with 1 setae; article 3, 1.5x, with 16 setae. Maxillula elongate without subdivision (Fig. 27E); longer than wide, 9.6x; at the tip, original median edge, with 4 robust setae. Maxilla with 2 distinct lobes; longer than wide, 4.7x (Fig. 27F); with endite longer than wide, 2.48x; medial lobe 0.05 mm wide, with 3 setae; lateral lobe 0.12 mm wide, with 2 setae. Maxilliped with 3 articles; longer than wide, 3.8x (Fig. 27G); without basal endite, without coxal oostegite lobe (epipod); articles longer than wide, article 1 (ischium), 2.6x; article 2 (merus), 1.6x; article 3 (carpus), 3.5x, with 3 robust setae. Posterior thorax appendages 7 pairs, each with 7 articles, coxae not dissected. Distal 6 articles forming functional leg (Figs. 28A–28E). Pereopod 1 (Fig. 28B) basipod longer than wide, 1.7x; ischium longer than wide, 1.7x; merus longer than wide, 0.8x; carpus longer than wide, 0.3x; propodus longer than wide, 1.6x; dactylus longer than wide, 3.3x; appendage with 1 seta on merus. Pereopod 4 (Fig. 28C) basipod longer than wide, 2.5x; ischium longer than wide, 2.0x; merus longer than wide, 0.7x; carpus as long as wide; propodus longer than wide, 2.2x; dactylus longer than wide, 3.5x; appendage with setae on ischium to propodus. Pereopod 6 (Fig. 28D) basipod longer than wide, 2.7x; ischium longer than wide, 1.9x; merus longer than wide, 0.7x; carpus longer than wide, 0.9x; propodus longer than wide, 2.2x; dactylus longer than wide, 3.2x; entire appendage without setae. Pereopod 7 (Fig. 28E) basipod longer than wide, 2.4x; ischium longer than wide, 2.8x; merus longer than wide, 1.7x; carpus longer than wide, 1.6x; propodus longer than wide, 3.7x; dactylus longer than wide, 4.2x; appendage with numerous setae and setae insertion areas on ischium to propodus.

Immature stage 3

**Figure 29:**
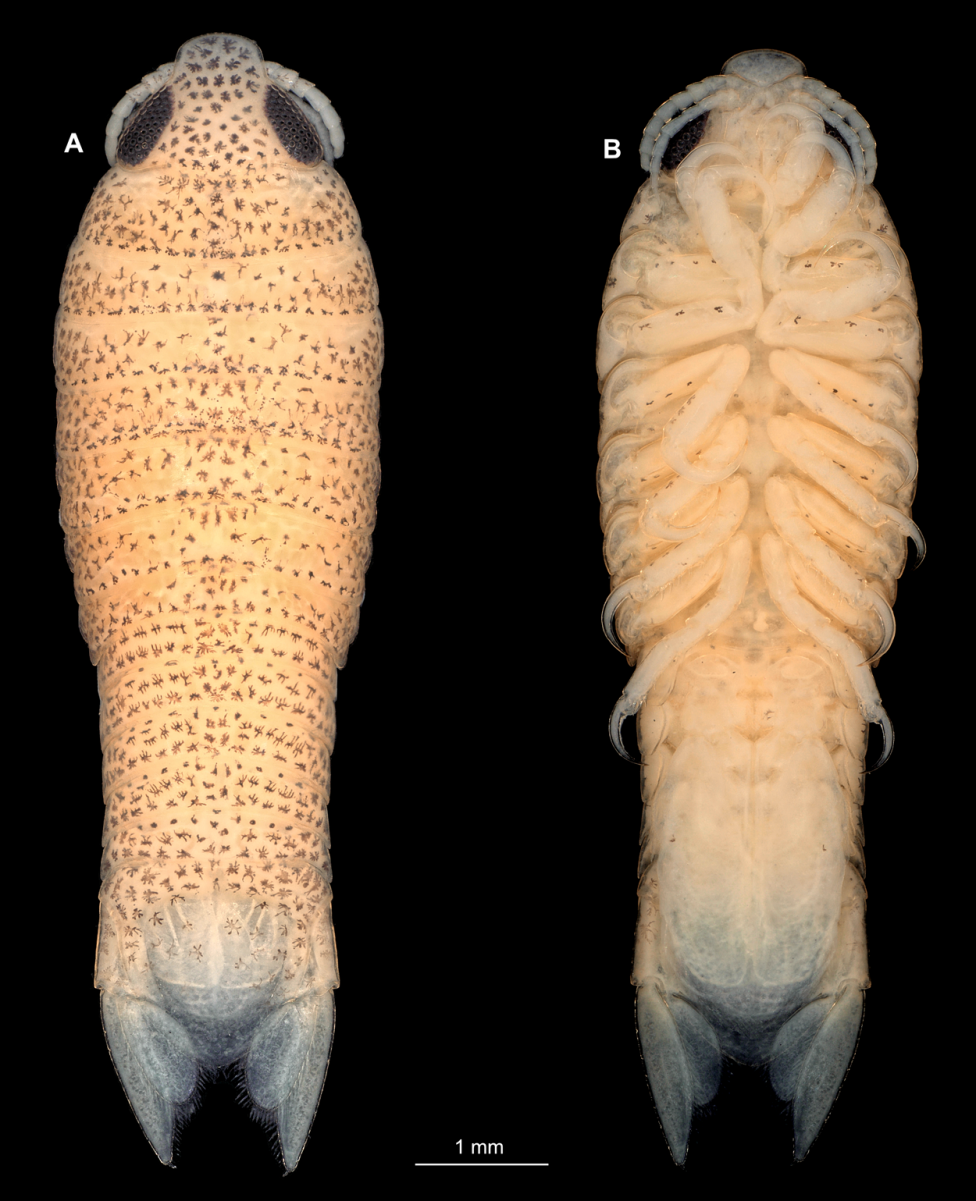
*Anilocra physodes* (Linnaeus, 1758) immature stage 3 (K15_4). (A) Dorsal view. (B) Ventral view.

**Figure 30:**
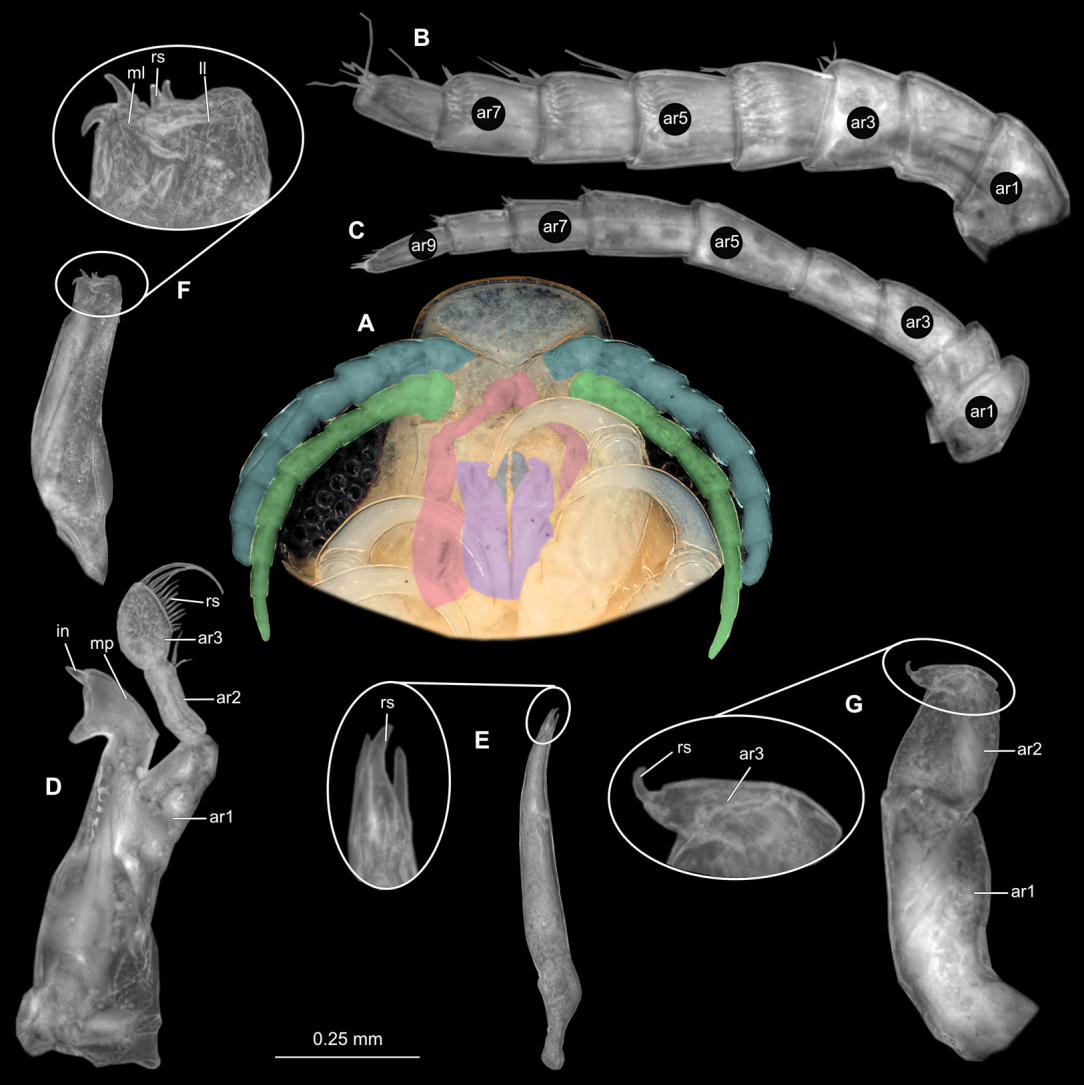
*Anilocra physodes* (Linnaeus, 1758) immature stage 3 (K15_4). (A) Ventral view of functional head with mouthpart positioning. (B) Antennula. (C) Antenna. (D) Mandible. (E) Maxillula. (F) Maxilla. (G) Maxilliped. ar1, article 1; ar2, article 2; ar3, article 3; ar5, article 5; ar7, article 7; mp, molar process; in, incisor; rs, robust setae; ml, medial lobe; ll, lateral lobe.

**Figure 31:**
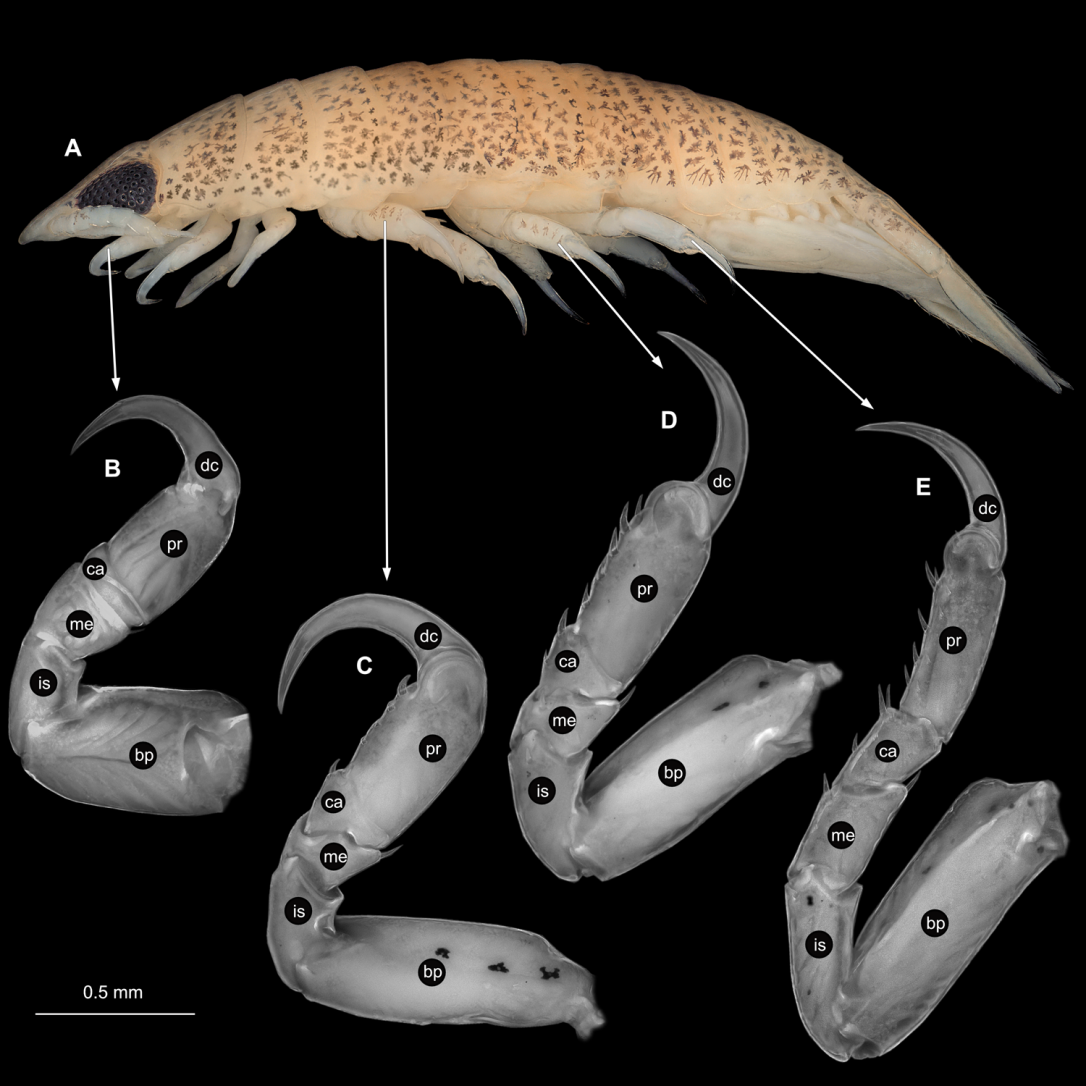
*Anilocra physodes* (Linnaeus, 1758) immature stage 3 (K15_4). (A) Lateral view. (B) Pereopod 1. (C) Pereopod 4. (D) Pereopod 6. (E) Pereopod 7. bp, basipod; is, ischium; me, merus; ca, carpus; pr, propodus; dc, dactylus.

Body longer than wide, 3.3x (Figs. 29A and 29B). Antennula 1.27 mm long, consists of 8 articles; articles 1 & 2 distinct; with setae on articles 3–8 (Fig. 30B); articles longer than wide, article 1, 0.9x; article 2, 1.0x; article 3, 0.8x; article 4, 0.9x. Antenna 1.27 mm long, consists of 9 articles; same length as antennula, thinner than antennula; with single setae on articles 6–9 (Fig. 30C); articles longer than wide, article 1, 0.7x; article 2, 0.6x; article 3, 1.3x; article 4, 1.6x. Mandible with proximal coxa, longer than wide in proximal-distal axis, 2.9x (Fig. 30D); distal palp with 3 articles, palp articles longer than wide, article 1, 2.3x; article 2, 3.0x, with 2 setae; article 3, 1.7x, with 12 setae. Maxillula elongate without subdivision (Fig. 30E), longer than wide, 8.6x; at the tip, original median edge, with 4 robust setae. Maxilla with 2 distinct lobes; longer than wide, 3.8x (Fig. 30F); medial lobe 0.03 mm wide, with 2 setae; lateral lobe 0.06 mm wide, with 2 setae. Maxilliped with 3 articles (Fig. 30G); without coxal oostegite lobe (epipod), without basal endit; all articles longer than wide, article 1 (ischium), 2.6x; article 2 (merus), 1.3x; article 3 (carpus), 2.7x, with 1 robust seta. Thorax appendages 7 pairs, each with 7 articles, coxae not dissected. Distal 6 articles forming functional leg (Figs. 31A–31E). Pereopod 1 (Fig. 31B) basipod longer than wide, 1.8x; ischium longer than wide, 1.7x; merus longer than wide, 0.8x; carpus longer than wide, 0.3x; propodus longer than wide, 2.5x; dactylus longer than wide, 3.4x; appendage with single spine-like seta on merus. Pereopod 4 (Fig. 31C) basipod longer than wide, 2.7x; ischium longer than wide, 1.8x; merus longer than wide, 0.6x; carpus longer than wide, 0.8x; propodus longer than wide, 2.0x; dactylus longer than wide, 3.9x; appendage with single spine-like seta on merus, 2 on carpus, 4 on propodus. Pereopod 6 (Fig. 31D) basipod longer than wide, 2.7x; ischium longer than wide, 2.0x; merus longer than wide, 0.7x; carpus longer than wide, 0.8x; propodus longer than wide, 2.3x; dactylus longer than wide, 4.3x. Pereopod 7 (Fig. 31E) basipod longer than wide, 3.0x, ischium longer than wide, 2.6x; merus longer than wide, 1.7x; carpus longer than wide, 1.4x; propodus longer than wide, 3.0x; dactylus longer than wide, 4.4x; appendage with 2 spine-like setae on merus, 3 on carpus, 11 on propodus.

Embryo

Body elongated, encapsulated in membrane, longer than wide, 1.6x (Fig. 10C). Antennula 0.25 mm long, longer than wide, 5.3x. Antenna 0.23 mm long, longer than wide, 5.9x. Maxilliped longer than wide, 4.2x. Pereopod 1 longer than wide, 5.4x. Pereopod 4 longer than wide, 4.5x. Pereopod 6 longer than wide, 4.0x. Eyes visible.

Remarks

*Anilocra* Leach, 1818, is a group of externally attaching crustaceans, parasitising marine fishes. Species of *Anilocra* can be distinguished by having a symmetrical body, body widest at trunk segment 5 and 6; ventrally folded rostrum; antennula shorter than antenna; mandible palp article 2 longer than article 3; maxilla lateral and medial lobe partly conjoined, each with 2 short robust setae. For full species synonymy and description of an ovigerous female, see Bruce (1987) and Öktener, Alaş & Türker (2018) respectively.

*Anilocra* was originally established with three species: *A. capensis* Leach, 1818, *A. mediterranea* Leach, 1818 and *A. cuvieri* Leach, 1818, of which the latter has been subsequently designated as type species by Kussakin (1979). *A. physodes* (Linnaeus, 1758) later became the senior synonym for *A. cuvieri* Leach, 1818 (see Trilles, 1975) and *A. mediterranea* Leach, 1818 (see Ellis, 1981). Ovigerous females of *A. physodes* are characterised by an oval body shape, two to three times as long as wide; head anterior margin truncate; antenna longer than antennula; and a semi-circular pleotelson with rounded posterior margin; and uropods reaching beyond posterior margin of pleotelson (Trilles, 1965; Kussakin, 1979).

*Anilocra physodes* has been predominantly recorded from the Mediterranean Sea, Black Sea, Northeastern Atlantic and the coast of the Iberian Peninsula (Kussakin, 1979), as well as from the North Sea (Holthuis, 1972) and Adriatic Sea (Trilles, 1994). The previous records of *A. physodes* from Naples, Italy, were by Guérin-Méneville (1832), Carus (1885), Nierstrasz (1915), Nierstrasz (1918), Dudich (1931) and Montalenti (1948). With the original description of *A physodes* as *Oniscus physodes* (Linnaeus, 1758), no illustrations were provided. Schioedte & Meinert (1884) later gave descriptions of an ovigerous female, male, ‘juvenile’ (immature stage 3) and ‘pullus 1’ (immature stage 1) specimens, along with a dorsal view illustration of each. These descriptions did not contain information on the mouthparts. More than 100 years later, Trilles (1965) described the ‘pullus 2’ (immature stage 2), male, and ovigerous female stages of *A. physodes*. The ‘pullus 1’ (immature stage 1) stage was described as the incubated stage in which the animal still has no setae or ornamentation. No description was provided for this stage. ‘Pullus 2’ was described as the stage where the animal has clear segmentation with a well-developed head, but with only 6 pairs of posterior thorax appendages (Trilles, 1965). This stage, as well as that of the male and ovigerous female stage, was described with simple characteristics of the mouthparts and pereopods along with illustrations. Trilles & Raibaut (1971) provided grayscale photographs of the dorsal view of a female and male specimen respectively. More detailed illustrations of the mouthparts and pereopods of an ovigerous female, male and immature stage 2 individuals of *A. physodes* were made by Trilles (1975). The ‘pullus 1’ (immature stage 1) dorsal view was not provided. During the same year, Lombardo (1975) described and illustrated the morphology of the head of *A. physodes* for comparison between, and distinction from other species. A schematic representation of the ventral head is provided with illustrations of all mouthparts and descriptions of antennula and antenna, mandible, maxillula and maxilla. The maxilliped was not included and no illustrations of antennulae and antennae were provided. Kussakin (1979) first described and illustrated the mouthparts and some pereopods of a non-ovigerous female specimen. More grayscale photographs of a female and male specimen were provided by Romestand (1979) and Trilles, Radujković & Romestand (1990). Öktener, Alaş & Türker (2018) gave illustrations of pereopods as well as illustrations and partial photos of mouthparts of an ovigerous female specimen.

The immature stage 3 specimen (K15_4) was deposited along with its fish host from which it was collected, but the position of the specimen on the host, was not recorded on the collection label. The host measures 33 mm in total length and was identified as a species of Gobiidae. The specimen is in a transitional stage between immature stage 2 and male, since it already has the last pair of pereopods fully developed, but poorly developed male characteristics (such as penes and appendix masculina on appendage of pleon segment 2). It is therefore referred to as an immature stage 3 or ‘young male’ (Trilles, 1975), during which the individual is already attached to a host and transforms into a male by moulting (see Brusca, 1978b; Aneesh et al., 2015). This stage was named by Brusca (1978b) as the ‘aegathoid’ stage. Jones et al. (2008) described this stage of development the ‘natatory-stage’, that has 7 pairs of pereopods; large eyes; appendages of pleon segments with natatory setae, specialised for swimming (see also discussion further below).

Specialised structure variation

Due to the softness and fragility of the gravid female mouthparts, the left maxilla was photographed and the image mirrored (Fig. 21F). The maxilliped was accidentally destroyed during dissection. The left counterpart was already missing upon loaning of the material. For comparison, the maxillipeds through ontogeny is compared to the illustrated maxilliped of the ovigerous female *A. physodes* by Trilles (1975) and Öktener, Alaş & Türker (2018).

Through the development from male to gravid female, *A. physodes* shows a decrease in the number of setae on antennulae. The gravid female specimen has one less article on both the antennula and antenna, compared to the immature, male and non-gravid female specimens. Antennulae and antennae total length proportions decreased, that is the immature and male having antennulae and antennae that differ only slightly in length, while the antennulae of the gravid female are almost half the length of the antennae. The shape and structure of the mandibles are similar for all 4 specimens. The different appearing incisor orientation of the non-gravid female specimen is purely due to placement during mounting of the mouthpart. Both the gravid female and male specimens have 17 setae on the lateral side of mandible palp article 3, whereas the non-gravid female counterpart has 11 and the immature has 12. The maxilla shape and morphology are similar within the immature, male and non-gravid female specimens. Both the male and non-gravid female maxillae possess an endite. This endite is absent on the maxilla of the immature and gravid female stages. Maxillulae are similar among specimens, despite slight damage on that of the gravid female. The maxilla of the gravid female specimen has a higher length to width ratio with a bulbous lateral projection. The medial and lateral lobes are partly conjoined, while they are separated within the non-gravid female and male specimens. Maxillipeds of the non-gravid female, male and immature stages are all without oosegite lobes, but the non-gravid female has a broader maxilliped palp article 1. The shape and structure of pereopods are consistent throughout ontogeny, with only an increase in relative size from immature to gravid female. Pereopods of immature specimens are more smooth and without setae insertion areas.

Gravid female structures can be compared to those of ovigerous females examined by Trilles (1965), Trilles (1975) and Öktener, Alaş & Türker (2018). Among the four sets of antennae and antennulae from the examined female specimens herein, the number of articles vary slightly with a maximum of 2 articles. The shape and structure of the mandibles show slight variation in the length-width ratios between palp articles 2 and 3. Article 3 from Trilles (1965), Trilles (1975) and Öktener, Alaş & Türker (2018) are shorter and more stout than the respective article 2, whereas Lombardo (1975) article 3 is equal in length and wider than article 2. The mandible palp article 3 is also equal in length, but narrower than article 2 (as in Fig. 21D). The maxillula (Fig. 21E) is slightly damaged at the midline, but corresponds overall to the shape and number of terminal robust setae of the maxillulae from Trilles (1965), Trilles (1975) and Öktener, Alaş & Türker (2018). All maxillae are similar except for the one from Trilles (1975), that does not seem to have the same lateral bulbous protrusion as the rest. From Trilles (1965) and Trilles (1975), the ovigerous female maxilliped is consistent in shape; with an oostegite lobe and endite (lined with plumose setae); and with between 3 and 4 terminal robust setae on article 3. The pereopods from Trilles (1965), Trilles (1975) and Öktener, Alaş & Türker (2018), are similar to those observed here (Fig. 22).

When comparing the specialised structures of the herein examined non-gravid female with the non-ovigerous specimen reported by Kussakin (1979), the morphology of mouthpart and pereopod structures are similar, with only the maxilla and maxillula of the latter, seem to be more stout. The number of terminal robust setae on maxilliped article 3 varies. Pereopod 1 and 7 can be compared to those described by Kussakin (1979), where the shape and structure of articles are similar, except for the larger carina on the basipod of pereopod 7 (Fig. 25).

The variation in male mouthpart morphology between the male *A. physodes* examined herein (see Fig. 27) and that of Trilles (1965) and Trilles (1975) are: the amount of setae on the antenna; the shape of mandible palp article 3, which varies from being terminally narrowly rounded (Fig. 27D and Trilles, 1975) to being broadly rounded (Trilles, 1965); and the presence of an endite on the maxilla, which has not been illustrated before. The remainder of the mouthpart structures are similar. With regards to the pereopods of males, those of Trilles (1965) have relatively longer carpi and dactyli, compared to those of our specimen (Fig. 27) and Trilles (1975).

The immature specimen was identified based on the morphological characteristics and descriptions provided by Trilles (1965) and Trilles (1975) for the young male and immature stages. From the latter publications, the immature herein, seems to be at a developmental stage closer to the male stage of Trilles (1965) than to the immature stage 3, but with dorsal chromatophores more similar in shape and distribution to the immature stage 2 of Trilles (1965). Mouthpart morphology of the herein described specimen is more similar to the male stage of Trilles (1965), whereas the pereopods are more similar to that of the immature stage 2 from the latter author.

Colour variation between specimens are mostly excluded from descriptive studies, due to the large number of factors that can alter the original colouration after collection, such as the preservation in alcohol. The colouration of specifically the externally attaching forms of Cymothoidae can provide some insight into the attachment position on the host. This observation of ‘countershading’ is explained by Körner (1982), where the darker side was the physiological upper side of the positioning of the parasite to the host. Species of *Anilocra* attach with the head to the anterior end of the fish host, as to be more streamline with the current of the water Comparing the dorsal colouration of the gravid and non-gravid female specimens, it is visible that the gravid female is darker coloured on the right side, while the non-gravid female is darker on the leftside. This leads to the suggestion that the gravid female was attached to the left lateral side of its fish host, while the non-gravid female was attached to the right lateral side. The colouration of the male specimen is uniform and symmetrical, most probably because it has not had time to develop these colour differentiations yet.

Morphometric analyses

**Figure 32:**
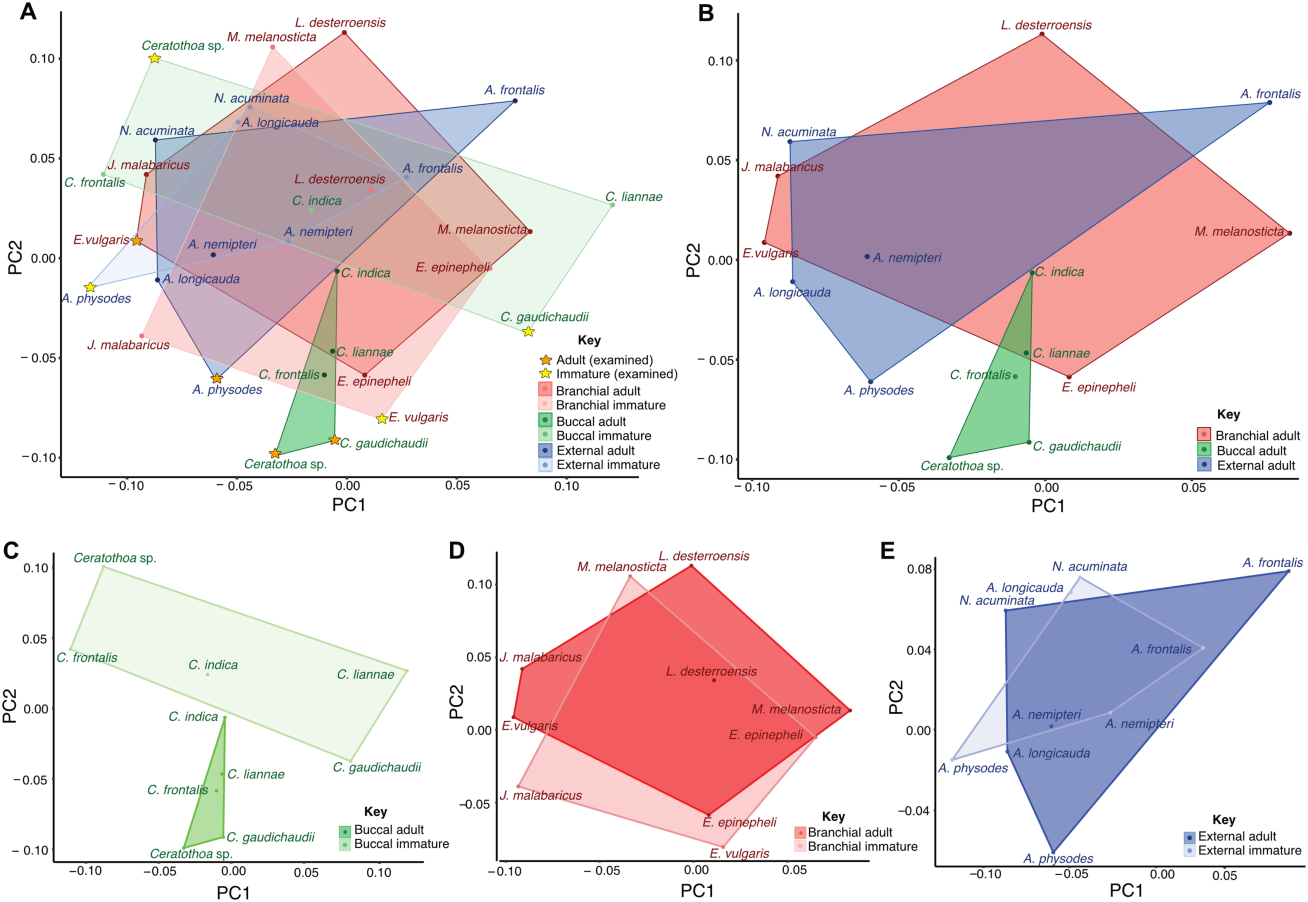
Principle component analysis of the shape of the hook of thorax appendage 2 (pereopod 1) of Cymothoidae species. (A) Adult and immature representatives of three different site attaching groups. (B) Adult representatives of three different site attaching groups. (C) Adult and immature representatives of mouth (buccal) attaching groups. (D) Adult and immature representatives of gill (branchial) attaching groups. (E) Adult and immature representatives of external attaching groups.

**Figure 33:**
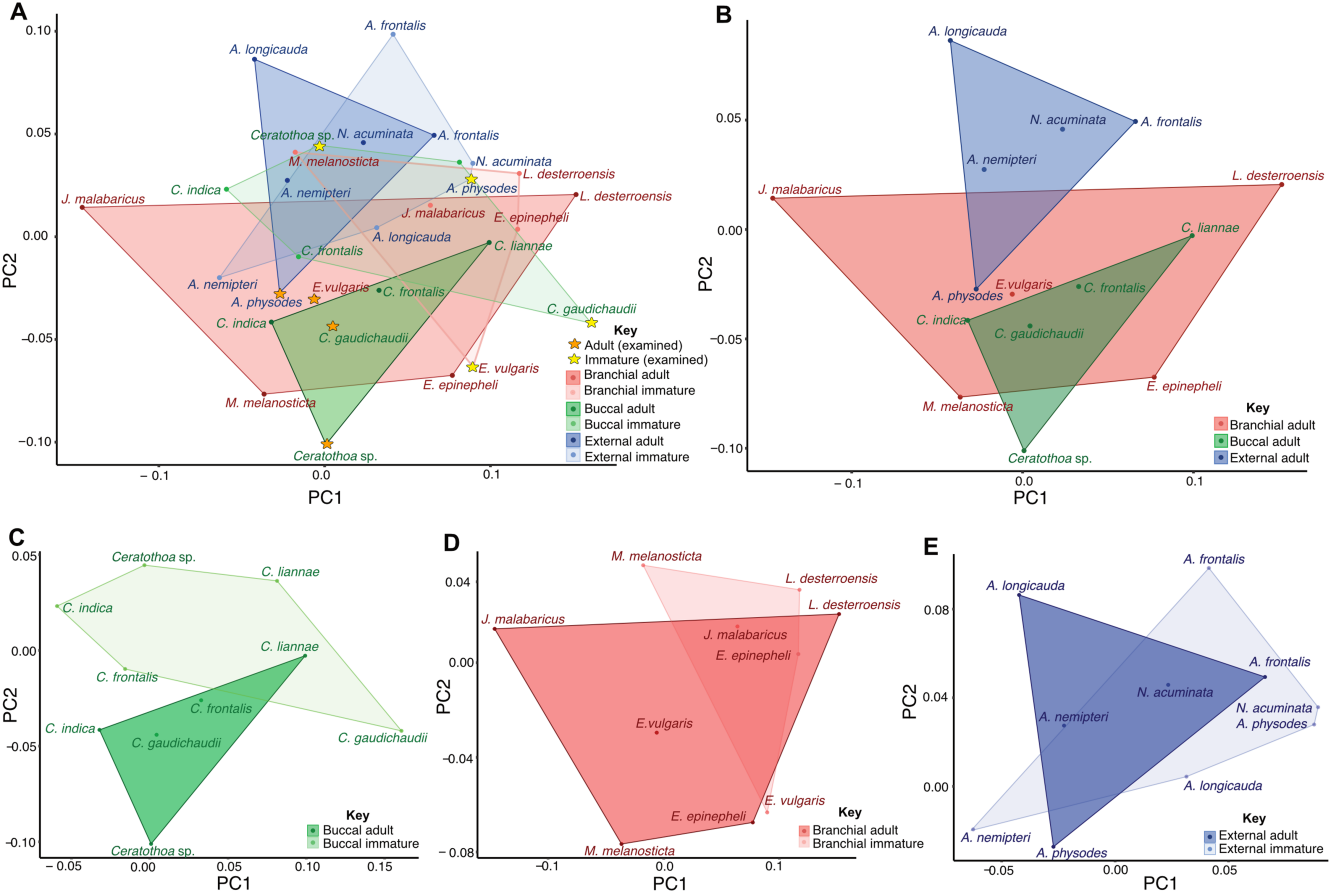
Principle component analysis of the shape of the hook of thorax appendage 7 (pereopod 6) of Cymothoidae species. (A) Adult and immature representatives of three different site attaching groups. (B) Adult representatives of three different site attaching groups. (C) Adult and immature representatives of mouth (buccal) attaching groups. (D) Adult and immature representatives of gill (branchial) attaching groups. (E) Adult and immature representatives of external attaching groups.

The dactyli of 15 species were included in the SHAPE analysis. The hook-like claws of thorax appendage 2 (pereopod 1) and hook-like claws of thorax appendage 7 (pereopod 6) of both adults and immatures (30 specimens), were all analysed together. In total 60 hooks were used for the analyses. The results are according to the most variable character states (PC1 to PC5; Fig. S5). The data imported to R for visualisation of the Morphospace is summarised in Table S2, with the code used to create the plots, in Data S1. The PCA plots of the elliptic Fourier analyses for the hooks of pereopod 1 of adult and immature individuals is presented in Figs. 32A–32E. The PCA plot of the elliptic Fourier analyses for the hook of pereopod 6 of adult and immature individuals is presented in Figs. 33A–33E. The first five PC values account for 95.5% of the total variation, with PC1 and PC2 accounting for 82.1%. PC1 explains 50.7% of the variation, while PC2 explains 31.5% thereof. The first dimension, here represented on the *X*-axis as PC1, is influenced by the degree of curvature of the dactylus where the dactyli is more curved toward the negative values and less curved towards the positive values. The second dimension, here represented on the *Y*-axis as PC2, is influenced by the thickness of the dactylus where the dactyli is thinner toward the negative values and thicker towards the positive values. Species examined in this study are indicated with a yellow star.

## Discussion

### The ontogeny of Cymothoidae

Isopoda is an ingroup of Peracarida. This latter group is characterised by a highly specialised brood care behaviour. Females form a brood pouch by structures arising from (some) of their thorax appendages (including the maxilliped, hence the one of the last appendage of the functional head). These structures on the appendages are called ‘oostegites’ and are thought to be modified epipods. Inside this pouch, yolk-rich eggs develop. The females that have eggs are here referred to as gravid females, since the term ‘ovigerous female’ may imply that the female has developed oostegites but that the brood pouch is still empty. The immatures may remain in the brood pouch for quite some time after hatching from the egg. These immatures in many cases seem to strongly resemble the adult (the ‘hatching as small adult’ paradigm, see Haug, 2020). Still some of these early stages have gained specific names over time. Also for early immatures of Cymothoidae there are quite a variety of names available.

For an individual still within the egg the case is easy and it is referred to as an embryo (Bakenhaster, McBride & Price, 2006). Aneesh et al. (2015) distinguishes between three stages of an embryo.

All stages after hatching, but before maturation will here be referred to neutrally as immatures. These immatures of Cymothoidae have often been collectively referred to as ‘larvae’ (Pillai, 1964; Williams & Bunkley-Williams, 1980; Bakenhaster, 2004; Trilles & Justine, 2006; Čolak et al., 2019). The term larva is highly problematic in many groups (see Haug, 2020) and has also caused quite some discussion in Isopoda (Boyko & Wolff, 2014). Some more specific terminologies have been proposed.

Immature stage 1 has hatched from the egg and is at first still inside the brood pouch. It has been called ‘pre-manca’ (Bakenhaster, McBride & Price, 2006; Aneesh et al., 2019b), ‘pre-hatch II’ (Adlard & Lester, 1995), ‘first mancal stage’(Bakenhaster, 2004), manca-I (Aneesh et al., 2018) or ‘pullus I’ (Schioedte & Meinert, 1884; Trilles, 1965; Thatcher, Jost & Souza-Conceição, 2003). It already has distinctly developed appendages and large eyes, but lacks the last pair of pereopods (Segal, 1987). In Adlard & Lester (1995), Bakenhaster, McBride & Price (2006), Aneesh et al. (2014), Martin, Bruce & Nowak (2015), Aneesh et al. (2015) and in this study, this immature stage lacks setae on pleon appendage, including uropods, and pleotelson. Immature stage 1 moults into the next stage (Brusca, 1978a; Adlard & Lester, 1995; Bakenhaster, 2004, Bakenhaster, McBride & Price 2006; Aneesh et al., 2015), and is then released from the brood pouch. It seems that immature stage 1 individuals do not feed but entirely depend on yolk.

Immature stage 2, has been referred to as ‘manca’ (Adlard & Lester, 1995; Sandifer & Kerby, 1983; Sartor & Pires, 1988; Aneesh et al., 2015), manca-II (Aneesh et al., 2018) or ‘pullus II’ (Schioedte & Meinert, 1884; Trilles, 1965; Thatcher, Jost & Souza-Conceição, 2003). This stage is able to swim as it has now numerous setae on pleon appendages, including the uropod and the pleotelson. It still has prominent eyes and still lacks the last pair of pereopods (Segal, 1987). These immatures largely depend on their remaining yolk for nutrition (Menzies, Bowman & Alverson, 1955; Brusca, 1978b; Sandifer & Kerby, 1983). Some authors suggested that they are already able to feed (Adlard & Lester, 1995; Fogelman, Kuris & Grutter, 2009), while some authors suggested that they do not feed (Menzies, Bowman & Alverson, 1955; Sandifer & Kerby, 1983). Sandifer & Kerby (1983) mention these immatures can survive for 1–2 weeks before risking starvation and predation. Cook & Munguia (2013) stated that the free-swimming ‘mancae’ of *Cymothoa excisa* Perty, 1833, became inactive after 3 days, but survived for over a week without a host. Lester (2005) mentioned that Cymothoidae ‘mancae’ can only survive for one or two days without a host. Those species that seem well able to feed (Adlard & Lester, 1995; Fogelman, Kuris & Grutter, 2009), can even alternate between hosts during this stage, prolonging their free-swimming stage and surviving, before permanently attaching to an ideal host in the next stage (Kroger & Guthrie, 1972; Lindsay & Moran, 1976; Sandifer & Kerby, 1983).

Immature stage 3 has been referred to as ‘natatory-stage’ (Jones et al., 2008), ‘juvenile’ (Aneesh et al., 2015) or ‘aegathoid’ (Brusca, 1978b). This stage still has large eyes, but differs from the earlier ones by possessing a pair of fully developed last pereopods. The body is streamlined and it has pleon appendages with numerous setae for swimming. They seem to lack any traces of yolk (Segal, 1987; Jones et al., 2008). This stage will search for a host and attach permanently to it.

In the next stage the transformation to a mature male begins. The individual will become wider, while most of the setae will be lost. In some groups, eyes become reduced in size, or are even absent. Sometimes the coloration changes as well (Jones et al., 2008). Since Cymothoidae are protandric hermaphrodites (Montgomery, 1895; Richardson, 1905; Mayer, 1879; Trilles, 1994). All juvenile broods are males (Woo, 2006), the male individual later develop and moult further into a female, when it is the first to attach to its host (Kensley, 1978). Other males that later attach to the same fish will remain male (Lester, 2005). When the female become gravid, developing a functional brood pouch it seems to become non-feeding. The oostegites cover a large section of the mouthparts, preventing the female from being able to feed (Menzies, 1954; Bunkley-Williams & Williams, 1998). These females therefore might feed right before the development of the oostegite lobes, and again immediately after the immatures have been released (Romestand, Thuet & Trilles, 1982; Adlard, 1989).

### Immatures of Cymothoidae in the literature

See Supplemental Document S9 and references therein, for a comprehensive list of depictions and description of immatures of Cymothoidae available from literature. In total, for 78 species of the 346 known and accepted species, there is at least one immature stage either described or partially described, illustrated or partly illustrated either by a drawing or photographs. Half of these 78 records were provided by Schioedte & Meinert (1884).

### Why should immature stages be important?

Bakenhaster (2004) stated that the individuals hatching from the brood pouch, might be in the most important life stage for phylogenetic studies, as it is immediately linked to a species specific adult female and has not yet developed any morphological variation, due to moulting or external, environmental factors. Descriptions of immatures should, therefore, enable better comparison between groups; as well as a better understanding of ontogeny and development; and some insight into the life history and evolution of different groups.

Given the role of immatures, as the infective stages, it is unfortunate that most known species of Cymothoidae are only well described and illustrated for the adult stages, but the immature stages remain largely undescribed and rarely figured (Bakenhaster, 2004; Jones et al., 2008). There are a number of reasons for the under representation of immature forms within species descriptions and other morphological studies. The most obvious reason is that, due to their syn-ecological relationship with fish and other marine animals, the adult females and males are usually caught and collected, attached to their host. The small, free-swimming immatures are not that easily encountered or caught (Stephenson, 1987; Jones et al., 2008). Brusca (1981) and Criscione, Poulin & Blouin (2005) stated that immature forms are morphologically very similar among different ingroups of Cymothoidae and do therefore not provide any apomorphic characteristics for descriptions or distinction between species and groups.

This statement has been demonstrated to be invalid for the group Cymothoida, as some studies have found that the immature stages of at least some species of Cymothoidae are indeed well distinguishable (Thatcher, Jost & Souza-Conceição, 2003; Bakenhaster, 2004; Jones et al., 2008) and do contribute specific morphological characteristics that provide insight into different life strategies and development.

### New data on embryos

The three embryonic stages herein examined (Figs. 10A–10C), were all collected from the brood pouch of the respective gravid female, hence their species identity is beyond doubt. These are the first detailed photographic images embryonic stages of Cymothoidae and the first images of embryos of the groups *Ceratothoa* and *Elthusa*. Only a few previous publications provided an illustration of one or more embryonic or marsupial stages of Cymothoidae: a developing embryo of *Nerocila californica* (see Brusca, 1978b); an embryo of *Cymothoa liannae*; a ‘pre-hatch’ of *Anilocra pomacentri* (see Adlard & Lester, 1995); an embryo of an unidentified Amazonian representative of Cymothoiiae (see Sartor, 1987); a *Glossobius hemiramphii* embryo see (Bakenhaster, McBride & Price, 2006); and an embryo of *Cymothoa frontalis* (see Aneesh et al., 2015). Aneesh et al. (2016) and Aneesh et al. (2018) both provided a photo of the embryos of *Mothocya renardi* and *Agarna malayi*, respectively.

The embryo of *Ceratothoa* sp. is notably the largest of the three examined here, even though it is an earlier, less developed stage than the other two embryos. The germ band is already segmented, some structures, especially the appendages of the ventral surface begin to differentiate. According to the scheme proposed by Aneesh et al. (2015), this individual is at a developmental stage between the embryonic stage 1 and 2, where the body is still sub-spherical or ovoid and without pigmented eyes, but structural differentiation has already begun. Wolff (2009) provided the stages of embryonic development of *Porcellio scaber* (Isopoda, Oniscidea), which is at least distantly comparable to that of Cymothoidae examined here, and represents the best available referencing system. According to Wolff (2009) and his scheme, the embryo of *Ceratothoa* sp. is interpreted as being in developmental stage 11. At this stage, limb buds of anterior head- and thoracic segments have elongated and show some differentiation; the visible pereopods have a homogeneous composition; and limb buds of developing pereopod 7 are not yet visible (Wolff, 2009), all of which is comparable to the embryo from Fig. 10A.

The embryos of *Elthusa* and *Anilocra* are both at embryonic stage 3, according to the scheme proposed by Aneesh et al. (2015), where the embryos are elongated, with visible eyes and appendages already differentiated into individual elements. When compared to the scheme of Wolff (2009), these embryos are interpreted to be in the developmental stage 13. During this stage, attachments of the pleon segments 1–3 exhibit a bilobed structure; and the pleotelson formation is visible.

Although the latter two embryos are almost equal in size and at the same developmental stage, some differences can be noted. The shape of the head of the embryo *Elthusa* is more pronounced with a medial indent, compared to the evenly rounded, more immersed head of the embryo of *Anilocra*. *Elthusa* have stouter, or wider antennulae and antennae, than *Anilocra*; the sternites are square-shaped sternites compared to the still rounded sternites of the embryo of *Anilocra*. The embryo of *Elthusa* has a shorter pleotelson than that of *Anilocra*.

Since so few embryos are available in the literature, it remains difficult to further compare these. The specimens reported here can hopefully be used in future studies that provide more embryos.

### Immature stages

This study presents the first lateral and ventral view of immature stages of the examined species. The individuals from this study are of slightly different developmental stages. The individual of *Ceratothoa gaudichaudii* is interpreted as being immature stage 1 developmental stage, obtained from the brood pouch of the female, still containing traces of yolk, visible through a semi-transparent body. The *Ceratothoa* sp. specimen is within the free-swimming immature stage 2, while the *Anilocra* specimen is an immature stage 3, with seven pairs of posterior thorax appendages.

Already within these early developmental stages, there are notable differences between the species. The most prominent differences are: (1) Pigmentation; (2) The thorax to pleon length ratio, with *Anilocra* having a pleon almost as long as the thorax and *Ceratothoa* sp. having a pleon much shorter than the thorax; (3) head shape; (4) size of the eyes; (5) mouthpart arrangement (ventral view); (6) number of setae on antennae and antennulae; (7) number and length of setae on mandible palp articles; (8) shape of thorax appendages (pereopods), with those of the externally attaching forms being more elongate than those of forms attaching to the gills or inside the mouth. This difference is already apparent in the immatures (Figs. 9, 27 and 30). This indicates that the specialisation for this life habit, develops as early as the embryonic stages. (9) Number of setae on pereopods; (10) the shape of the pleotelson posterior margin; (11) uropod shape and length.

All of the examined immatures displayed well developed, functional mouthparts and at least 6 posterior thorax appendages with well-developed distal hooks. Even though these structures differ in shape and size, from those of the adult stages, they are developed in the same arrangement and are already specialised in function for feeding. These variations therefore, do not affect the function of the individual structures.

Bakenhaster (2004) mentions that the morphological features of free-swimming immature stages, are most likely not affected by the eventual attachment site on the host. This statement is supported by examined immatures. All stages also have a long, slender and streamline body shape, specialised for swimming and searching for a host. This, and the characters mentioned by Jones et al. (2008) for natatory stage individuals, are ‘immature/larval specific characters’ that can be used to distinguish between life stages. These differences cause immatures to look very different than adults. From personal observation, isolated immatures (collected without the adults) cannot be identified by comparing it to adult morphological characteristics or ‘species diagnosis’ as these do mostly not apply to immatures. Even so, there were many differences between immatures to identify an immature to a group of species (genus?), if there are sufficient illustrations or descriptions available for comparison. The problem is therefore not that immature stages all exhibit similar morphology, making them insignificant for morphological studies, but rather that the lack of descriptions, illustrations and attention to immatures have led to the idea that they cannot be distinguished from adults and that they are somewhat insignificant.

### Males

Male specimens of the examined groups already provide some insight into the attachment site on the host, as their body form has begun to specialise. The male of *Anilocra* has a straight body with lateral margins that are almost parallel, streamlined for attachment on the exterior surface of a host. Its posterior thorax appendages are longer and more slender, possibly to enable its ventral body the closest possible contact to the host for better attachment. The body of the male of *Elthusa* has already started to widen at thorax segments 4–6. In addition, the pereopods of the latter are more stout, less extended attachment to gill arches. The body of the male of *Ceratothoa* has widened slightly more toward the posterior end, with posterior thorax appendages extended disto-ventrally, appearing to ‘hang’ further down from the body (Fig. 19A). This orientation of pereopods in this way is most likely to enable it to grasp as far as possible around the tongue of its host for better attachment. All males have lost pleon and uropodal setae. Other general changes in the transition between immature and male are the reduction in eye sizes and pigmentation spots (Jones et al., 2008).

### Females

The morphological characters of Cymothoidae and the variation thereof, are as mentioned, largely based on the adult female structures. These characters are used to create a diagnosis for the specific group that should enable the differentiation between species and supra-specific groups. A constant feature of an ovigerous and/or gravid female is the presence of oostegite lobes arising from the maxilliped coxa. These are absent from non-ovigerous and/or non-gravid females as they do not yet have developed oostegites forming the brood pouches. Further effects of the attachment site is most prominent in the females: Comparing the mouthpart positioning between the females of the groups, the externally attaching *Anilocra* and the gill attaching *Elthusa*, has a more flattened mouthpart arrangement that is positioned more to the posterior end of the head, while those of *Ceratothoa*, are positioned closer to the anterior end. The overall arrangement of the mouthparts are similar among the species, forming a mouth cone, to enable the sucking of host tissue, blood and/or mucus. A similar arrangment of the mouthpart of an externally attaching parasite is known for *Nerocila* (Nagler & Haug, 2016).

### General comments on morphospace analyses

It is known that attachment site influences the shape of these appendages. In species that burrow inside the flesh of a host, posterior thorax appendages are usually shorter, than those of species inside the mouth (Thatcher, 2000). This might be a developmental result of space restriction. Baillie et al. (2019) showed that the shape of the hooks is correlated to the site of attachment. They used exclusively adult females and applied a semi-landmark approach. Here we used outlines instead of semi-landmarks in order to reduce choice dependent influences.

Our results partly differ from those of Baillie et al. (2019) due to the design of the approach:

(1) Baillie et al. (2019) did not include any immature individuals, only adult female specimens. The use of immatures (and the lack of descriptions and illustrations) in the analysis herein, have restricted the overall number of individuals that can be used in the dataset, hence our sample size is smaller. (2) Baillie et al. (2019) analysed the hook of pereopod 7. Due to the inclusion of immature individuals, which often still lack this appendage, using the hook-like claws of pereopod 6 was next possible option.

### Hook of pereopod 1 (thorax appendage 2)

In the analysis of the shape of the hook of pereopod 1 (Figs. 32A–32E) not all adult separate well according to their site of attachment. The group of forms attaching externally separate from those attaching inside the mouth. Yet, both have overlapping areas with the group of forms that attach to the gills. In the analysis of Baillie et al. (2019) the externally attaching form separate well from those attaching to gills and inside the mouth, yet the latter two strongly overlap.

Some species show a large ontogenetic change in shape of the hook-like claws through development from immature to adult (Fig. 32A such as *Ceratothoa* sp, *C. frontalis*, *C. liannae*, *E. vulgaris* and *M. melanosticta*), while that of some other species (*C. indica* and *A. epinepheli*) show a rather minimal change. The morphological variation through ontogeny occurs in all directions, either increasing or decreasing in thickness, or becoming more or less curved into adulthood. These changes seem not correlated to attachment site, and the direction of change is not the same. From this analysis, there exists no general trajectory in which the claw of pereopod 1 changes throughout ontogeny.

For the forms attaching externally or at the gill there seems to be no separation between adult and immature morphologies (Figs. 32D and 32E). In contrast, there is a clear separation between adults and immatures of forms attaching inside the mouth (Fig. 32C). Here the hook-like claws have a more pronounced curvature in the immature stages, while all of their adults have significantly less curved claws.

This indicates that the hook-like claws of pereopod 1 in forms attaching inside the mouth, become more restricted concerning their curvature. In addition, all of them show a decrease in thickness from immatures to adults. The adults of forms attaching inside the mouth, occupy the smallest morphospace, indicating a small range of variation in shape, while the adults attaching to gills occupy the largest space, indicating a wide range of variation in dactylus shape. This is in contrast to the results of Baillie et al. (2019), where the adults attaching inside the mouth occupy the largest space and the adults attaching to the gills occupy the smallest space.

### Hook of pereopod 6 (thorax appendage 7)

Comparably, the analysis of the hook-like claw shape of pereopod 6 (Figs. 33A–33E) adults of externally attaching forms and those attaching inside the mouth, provide well separated clusters (Fig. 33B). Both overlap the area occupied by forms attaching to the gills. Especially here, the pattern gives at least a hint towards a phylogenetic pattern. Although recent phylogenetic analyses (Baillie et al., 2019) provided a more complex pattern, it is still reasonable to assume that external attachment is the most ancestral strategy. The attachment to the gills would then most likely be the next derivation and from such a strategy the attachment inside the mouth could be even more derived one. Hence, the change along principle component 2 could potentially be related to this evolutionary transition. A larger data set with a phylogenetic correction approach would be desirable for further exploring this possibility.

Similar to the analysis of the hook-shaped claw of pereopod 1, we also see a lot of ontogenetic changes on the hook of pereopod 6 in some species (such as *C. gaudichaudii* and *J. malabaricus*) while that of some other species (*A. frontalis*, *L. desterroensis* and *C. frontalis*) show a rather minimal change.

None of the adult-immature combinations separate from each other (Figs. 33C–33E). Yet, as for the hook of the pereopod 1 and 6, the adults and immatures of the forms attaching inside the mouth almost separate (Fig. 33C), with the adults having a thinner dactylus than those of the immatures.

When considering only the species examined herein (*Ceratothoa* sp, *C. gaudigaudii*, *E. vulgaris* and *A. physodes*), there is a clear separation between adult and immature in the shape of the hook of pereopod 6. The adults of the examined species have relatively thinner, more curved hooks than the immatures. Here, again, adults attaching to the gills occupy the largest area of the morphospace, while that of the forms attaching externally or inside the mouth occupy smaller areas, almost equal in size. This is different to the results of Baillie et al. (2019; see above).

The resolved shapes also differ. The externally attaching adults have thicker hook-like claws of pereopod 6 than forms attaching to gills or inside the mouth. This is the opposite in Baillie et al. (2019), where pereopod 6 of externally attaching adults are all thinner than the that of the other forms. These differences may be a result of the sampled species.

Our study most likely has a lower signal to noise ration compared to that of Baillie et al. (2019), a possible reason why we do not get a better separation of some groups. The higher noise come is as we had to use images from different authors documented with very different methods. Yet, we can demonstrate with this relatively small data set, that the morphology of the hook-like claws of pereopods are not driven by adaptations to the attachment site alone. Firstly, there might be a phylogenetic signal correlated to it. Even more importantly, the morphology appears to be heavily driven by the ontogeny.

This might mean that the strategies of the immatures still differ from that of the adults. This could also mean that some of the evolutionary changes of hook-like claw shape might be caused by evolutionary shifts in developmental patterns (heterochrony).

The variation in shape between the anterior pereopod 1 hook and the posterior pereopod 6 hook, was considered as well. Baillie et al. (2019) mentioned that variation between the anterior and posterior dactyli may suggest that these dactyli function differently. Our results indicate that the hook of pereopod 1 is usually more curved than those of 7, supporting principle observations of Baillie et al. (2019).

Baillie et al. (2019) further mentioned that these variations are smaller for the forms attaching to the gills and inside the mouth. From our results, the hook of pereopod 6 of mouth attaching adults, have a much wider range of variation in curvature than the hook-like claw of pereopod 1, while usually being less curved than the latter. The immature representatives do not show a general change in shape variation between the hook-like claw of pereopod 1 and that of 6. The hook-like claw of pereopod 6 of gill attaching adults, typically have a wider range of variation in curvature, but a narrower range of variation in thickness than the hook of pereopod 1. Baillie et al. (2019) found the posterior pereopod of gill attaching groups to be similar in morphology. Immatures of gill attaching genera show a less curved hook of pereopod 6 and a more curved hook of pereopod 1, with the latter having a wider range of thickness than that of pereopod 6.

## Conclusions and Outlook

A major challenge in improving the understanding of ecology and evolution of the group Cymothoidae, is the very imperfect knowledge about the ontogeny. Approximately 22% of all species of Cymothoidae have at least some information on immature stages. Even so, for most of these records, there is only single illustrations and a short description.

Here we amended this still incomplete data set, including even embryonic stages. We can support that immatures can also be well separated from each other, if proper comparative data are available. Quantitative analysis supports earlier attempts, but indicates that ontogeny indeed plays a major role for the shape of the attaching structures, the hooks of the pereopods.

We would need a larger sample of immatures for improving the situation. Ideally, we should have shapes through several successive stages for being able to reconstruct trajectories.

It needs to be especially emphasised that parasitic forms of Isopoda do not hatch as small adults and that the immatures are the infective stages. Hence these play indeed a major role in understanding in the ecology and evolution of the group.

## Supplemental Information

10.7717/peerj.9181/supp-1 Supplemental Information 1 Immature hook of thorax appendage 2 outlines used for the elliptic Fourier analysis. Sources of the original illustrations are given in Supplemental Table S1. (A) *A. frontalis*. (B) *A. longicauda*. (C) *A. nemipteri*. (D) *A. physodes*, (E) *N. acuminata*. (F) *C. frontalis*. (G) *C. gaudichaudii*.(H) *C. indica*. (I) *C. liannae*. (J) *C. sp*. (K) *E. epinepheli*. (L) *E. vulgaris*. (M) *J. malabaricus*. (N) *L. desterroensis*. (O) *M. melanosticta*.

10.7717/peerj.9181/supp-2 Supplemental Information 2 Immature hook of thorax appendage 7 outlines used for the elliptic Fourier analysis. Sources of the original illustrations are available in Table S1. (A) *A. frontalis*. (B) *A. longicauda*. (C) *A. nemipteri*. (D) *A. physodes*. (E) *N. acuminata*. (F) *C. frontalis*. (G) *C. gaudichaudii*. (H) *C. indica*. (I) *C. liannae*. (J) *C. sp*. (K) *E. epinepheli*. (L) *E. vulgaris*. (M) *J. malabaricus*. (N) *L. desterroensis*. (O) *M. melanosticta*.

10.7717/peerj.9181/supp-3 Supplemental Information 3 Adult hook of thorax appendage 2 outlines used for the elliptic Fourier analysis. Sources of the original illustrations are available in Table S1. (A) *A. frontalis*. (B) *A. longicauda*. (C) *A. nemipteri*. (D) *A. physodes*. (E) *N. acuminata*. (F) *C. frontalis*. (G) *C. gaudichaudii*. (H) *C. indica*. (I) *C. liannae*. (J) *C. sp*. (K) *E. epinepheli*. (L) *E. vulgaris*. (M) *J. malabaricus*. (N) *L. desterroensis*. (O) *M. melanosticta*.

10.7717/peerj.9181/supp-4 Supplemental Information 4 Adult hook of thorax appendage 7 outlines used for the elliptic Fourier analysis. Sources of the original illustrations are available in Supplemental Table S1. (A) *A. frontalis*. (B) *A. longicauda*. (C) *A. nemipteri*. (D) *A. physodes*. (E) *N. acuminata*. (F) *C. frontalis*. (G) *C. gaudichaudii*. (H) *C. indica*. (I) *C. liannae*. (J) *C. sp*. (K) *E. epinepheli*. (L) *E. vulgaris*. (M) *J. malabaricus*. (N) *L. desterroensis*. (O) *M. melanosticta*.

10.7717/peerj.9181/supp-5 Supplemental Information 5 Visualisation of the first 5 Principal Components of hooks of thorax appendages 2 and 7 shapes from the PCA. The first two PC values were used to present in the Morphospace as those that had the most influence on the variation. PC1 represents the variation in curvature, while PC2 represents the variation in thickness of the hook.

10.7717/peerj.9181/supp-6 Supplemental Information 6 Sources of the original hook of thorax appendage 2 and 7 illustrations used in the Morphospace dataset.

10.7717/peerj.9181/supp-7 Supplemental Information 7 Data imported to R for visualisation of Morphospace plots.

10.7717/peerj.9181/supp-8 Supplemental Information 8 R code used for visualisation of PCA values of dactyli shape.

10.7717/peerj.9181/supp-9 Supplemental Information 9 Raw morphospace values generated for all examined and redrawn species, transformed from chain codes of all dactyli outlines. Original nef. file from which the PC values originated.

10.7717/peerj.9181/supp-10 Supplemental Information 10 A comprehensive list of depictions and description of immatures of Cymothoidae available from literature.

## Additional Information and Declarations

### Data Availability

The following information was supplied regarding data availability: The data and R code are available in the Supplemental Files. The material/specimens examined in this study are available in the Centrum für Naturkunde, Zoological Museum (CeNak), Hamburg (K23191; K34618; K26553; K19211); as well as in the Bavarian State Collection of Zoology (ZSM), Munich (K15_4).
