# Supplementary figures and images for: Shape of attachment structures in parasitic isopodan crustaceans: the influence of attachment site and ontogeny
Source: PeerJ. 2020 Jun 18;8:e9181. doi: 10.7717/peerj.9181 (PMC7306222; doi:10.7717/peerj.9181)

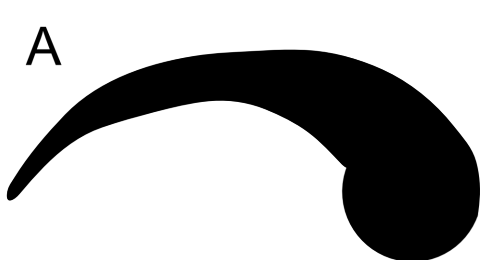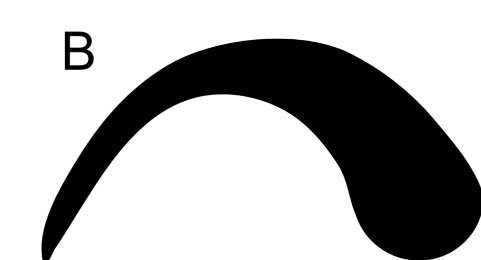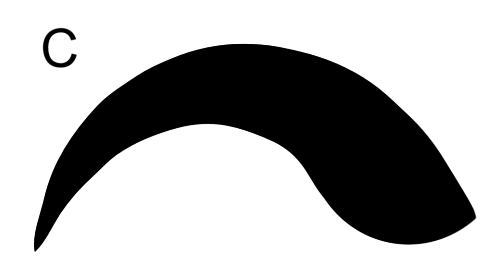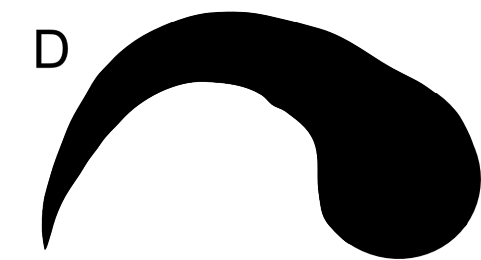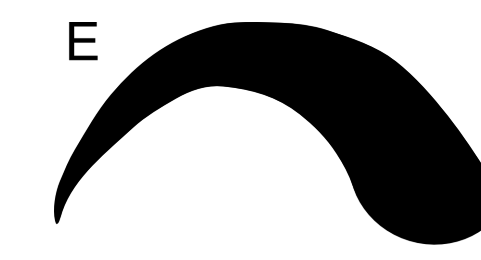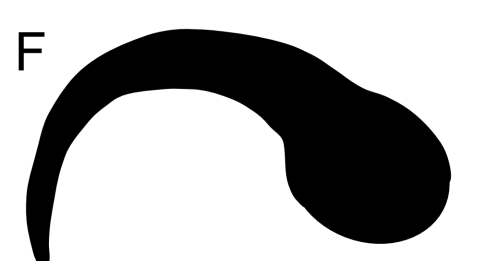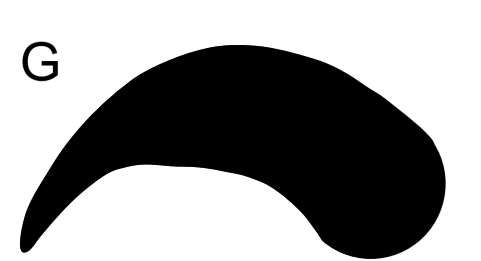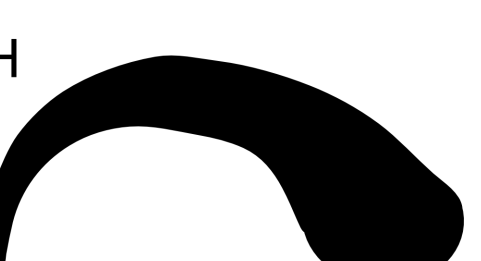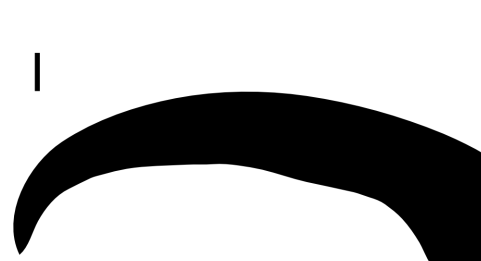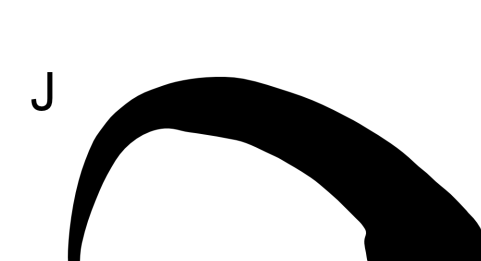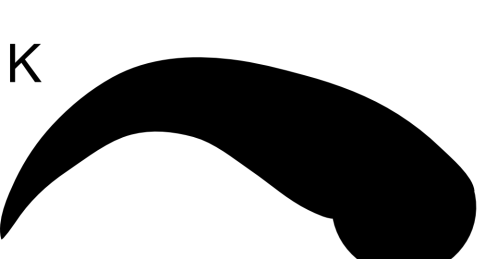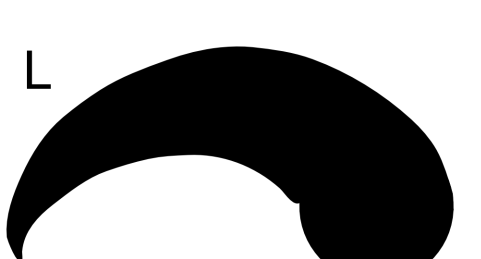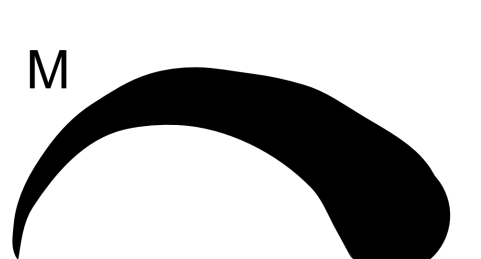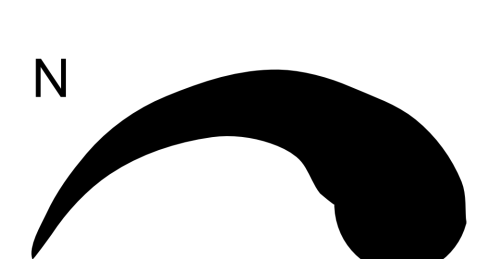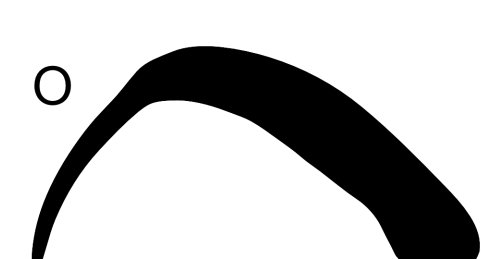

Supplement: Supplemental Information 1 — (A) A. frontalis. (B) A. longicauda. (C) A. nemipteri. (D) A. physodes, (E) N. acuminata. (F) C. frontalis. (G) C. gaudichaudii.(H) C. indica. (I) C. liannae. (J) C. sp. (K) E. epinepheli. (L) E. vulgaris. (M) J. malabaricus. (N) L. desterroensis. (O) M. melanosticta. [file peerj-08-9181-s001.pdf]

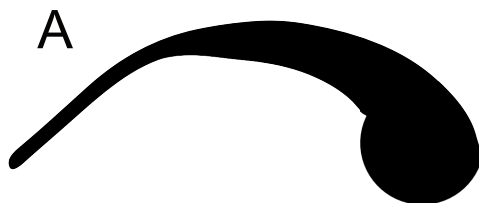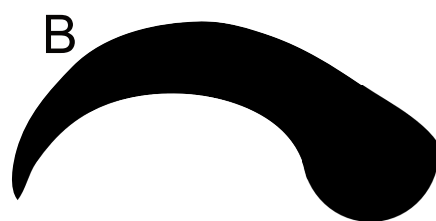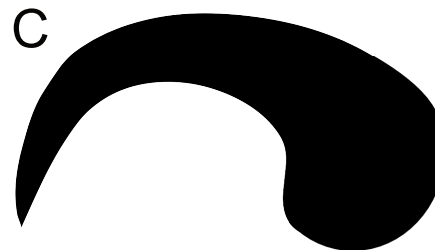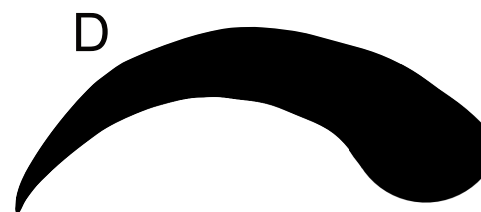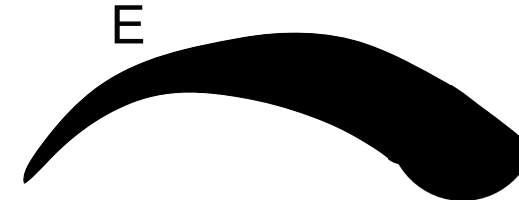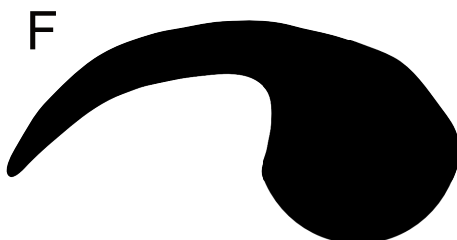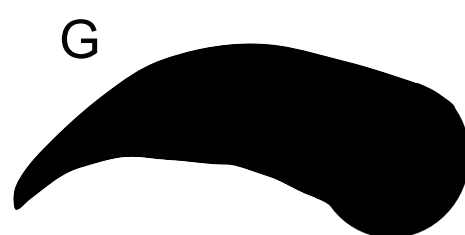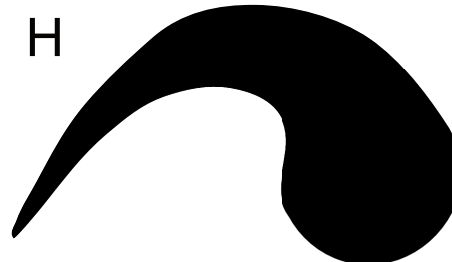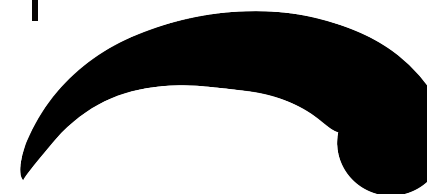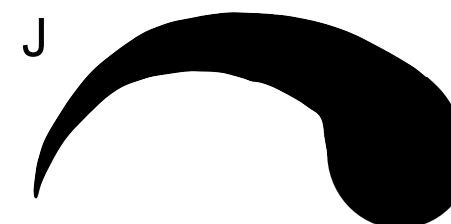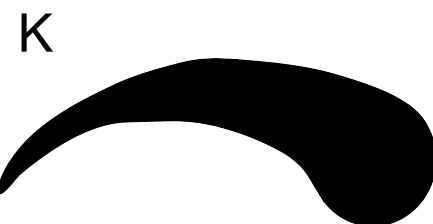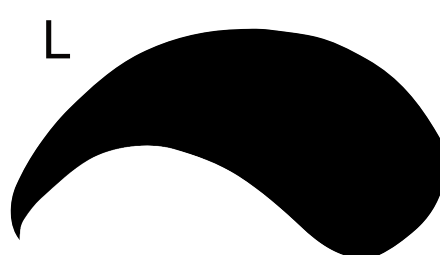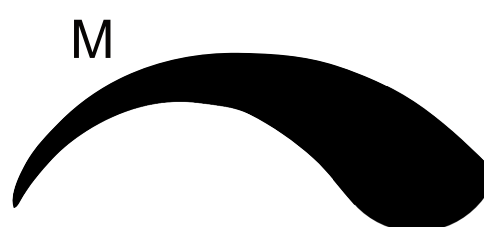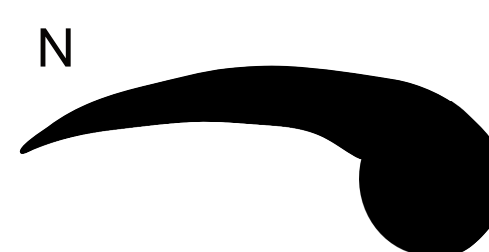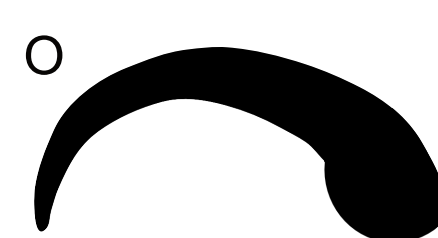

Supplement: Supplemental Information 2 — (A) A. frontalis. (B) A. longicauda. (C) A. nemipteri. (D) A. physodes. (E) N. acuminata. (F) C. frontalis. (G) C. gaudichaudii. (H) C. indica. (I) C. liannae. (J) C. sp. (K) E. epinepheli. (L) E. vulgaris. (M) J. malabaricus. (N) L. desterroensis. (O) M. melanosticta. [file peerj-08-9181-s002.pdf]

A

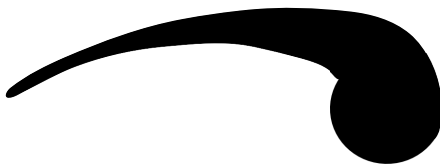

B

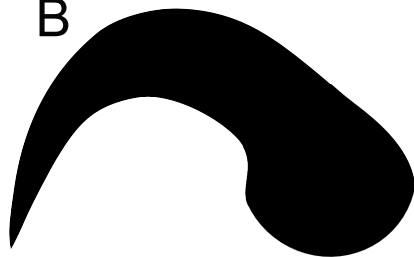

C

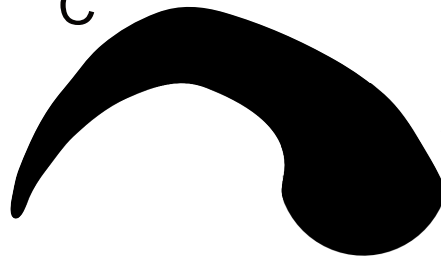

D

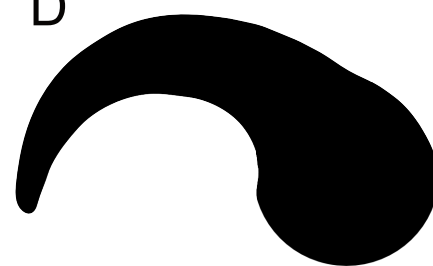

E

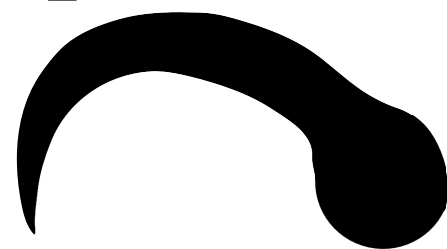

F

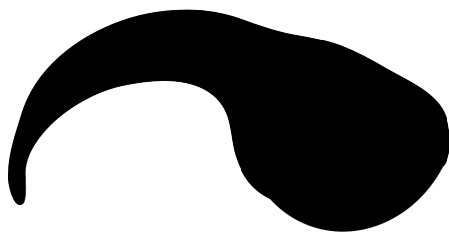

G

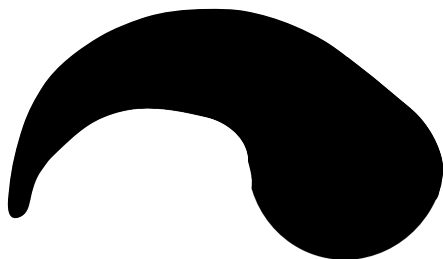

H

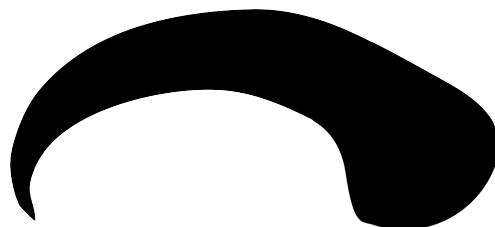

I

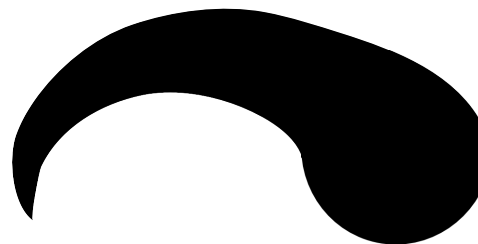

J

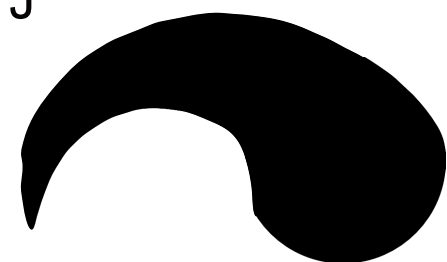

K

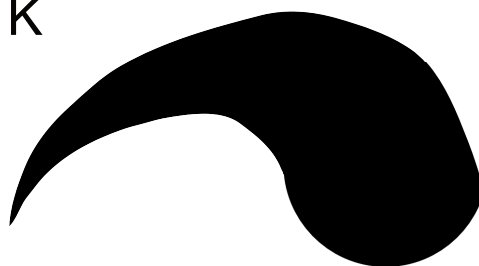

L

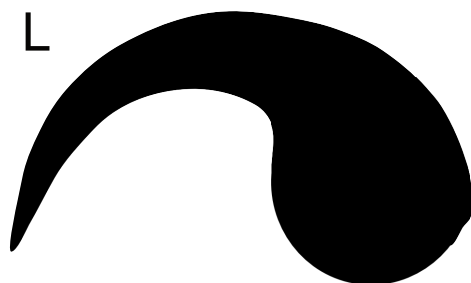

M

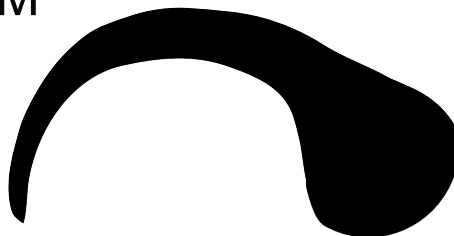

N

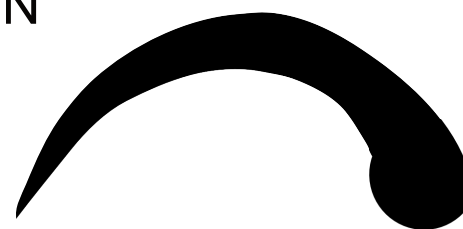

O

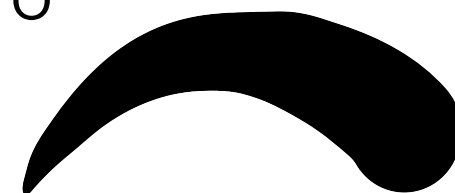

Supplement: Supplemental Information 3 — (A) A. frontalis. (B) A. longicauda. (C) A. nemipteri. (D) A. physodes. (E) N. acuminata. (F) C. frontalis. (G) C. gaudichaudii. (H) C. indica. (I) C. liannae. (J) C. sp. (K) E. epinepheli. (L) E. vulgaris. (M) J. malabaricus. (N) L. desterroensis. (O) M. melanosticta. [file peerj-08-9181-s003.pdf]

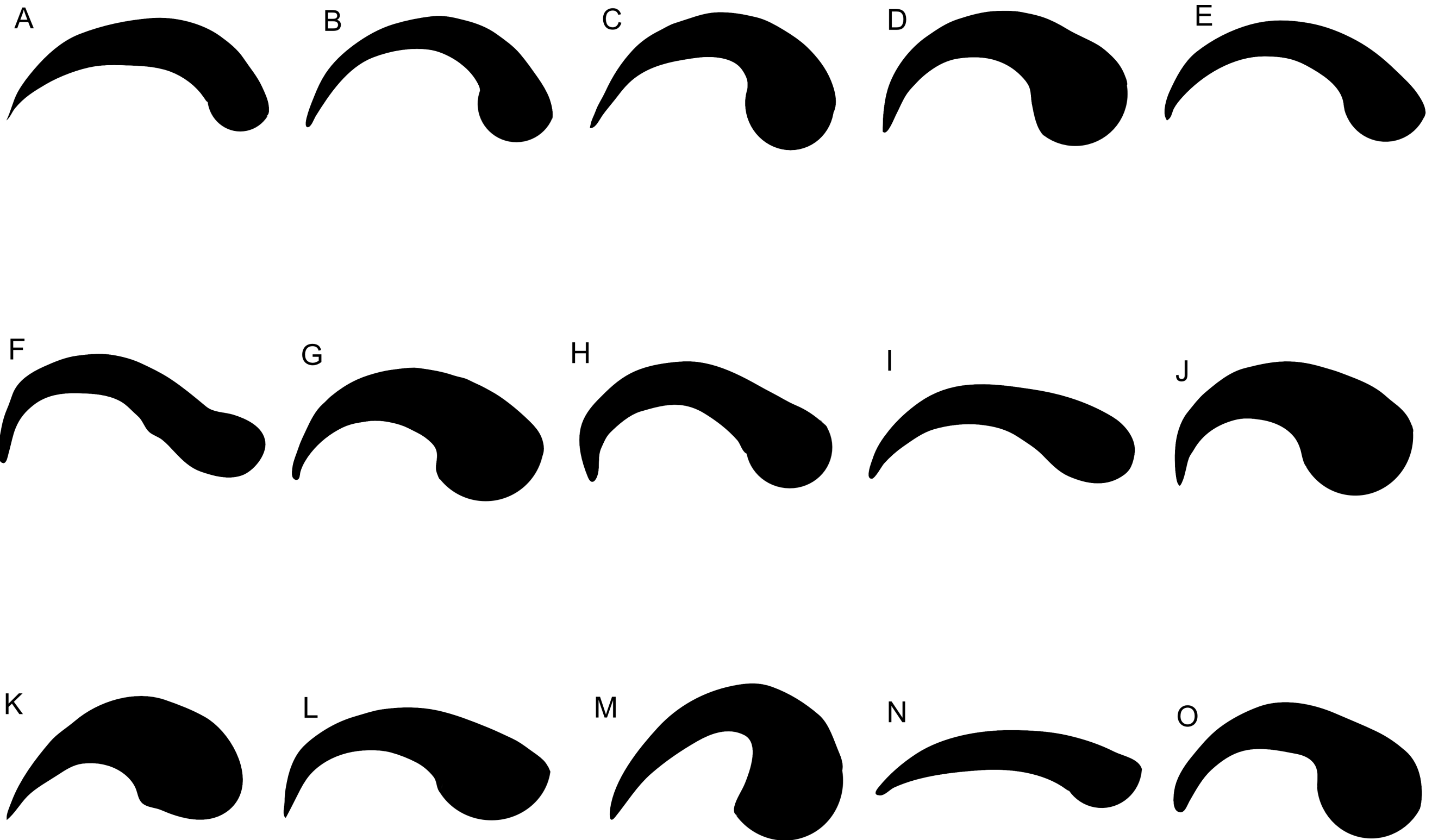

Supplement: Supplemental Information 4 — (A) A. frontalis. (B) A. longicauda. (C) A. nemipteri. (D) A. physodes. (E) N. acuminata. (F) C. frontalis. (G) C. gaudichaudii. (H) C. indica. (I) C. liannae. (J) C. sp. (K) E. epinepheli. (L) E. vulgaris. (M) J. malabaricus. (N) L. desterroensis. (O) M. melanosticta. [file peerj-08-9181-s004.pdf]

-2S.D.

Mean

+2S.D.

PC1

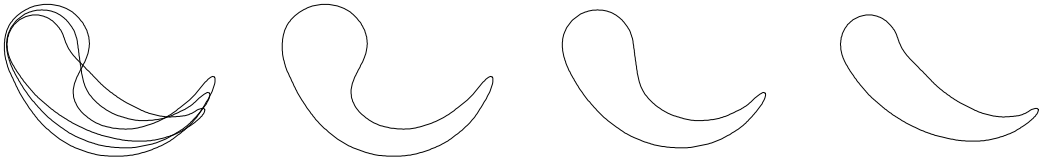

PC2

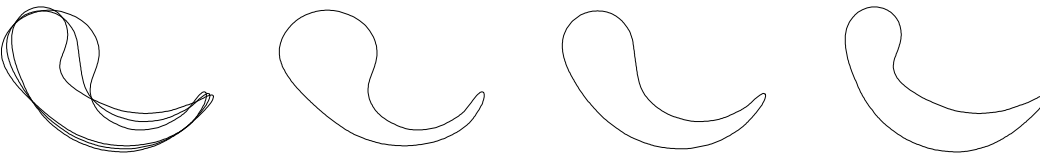

PC3

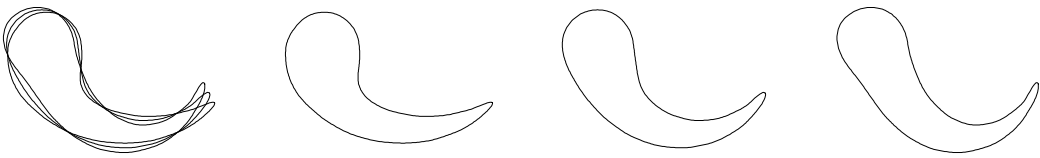

PC4

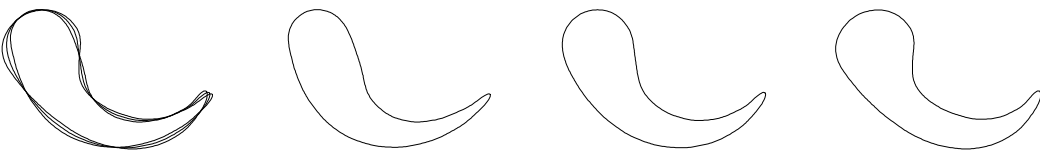

PC5

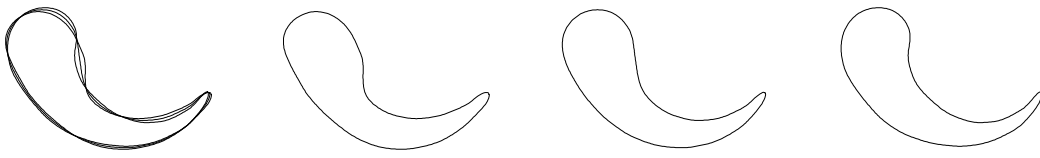

Supplement: Supplemental Information 5 — The first two PC values were used to present in the Morphospace as those that had the most influence on the variation. PC1 represents the variation in curvature, while PC2 represents the variation in thickness of the hook. [file peerj-08-9181-s005.pdf]
