# Supplementary material for: Shape of attachment structures in parasitic isopodan crustaceans: the influence of attachment site and ontogeny
Source: PeerJ. 2020 Jun 18;8:e9181. doi: 10.7717/peerj.9181 (PMC7306222; doi:10.7717/peerj.9181)
Supplement: Supplemental Information 6 [file peerj-08-9181-s006.pdf]

Species	Adult	Immature
A. frontalis Milne Edwards, 1840	Trilles (1965)	Trilles (1965)
A. longicauda Schioedte & Meinert, 1881	Bruce (1987)	Jones et al. (2008)
A. nemipteri Bruce, 1987	Bruce (1987)	Jones et al. (2008)
A. physodes (Linnaeus, 1758)	This study	This study
N. acuminata Schioedte & Meinert, 1881	Bruce (1987)	Bakenhaster (2004)
C. frontalis Milne Edwards, 1840	Aneesh et al. (2015)	Aneesh et al. (2015)
C. gaudichaudii (Milne Edwards, 1840)	Schioedte & Meinert (1884)	This study
C. indica Schioedte & Meinert, 1884	Trilles & Bariche (2006)	Jones et al. (2008)
C. liannae Sartor & Pires, 1988	Sartor & Pires (1988)	Sartor & Pires (1988)
Ceratothoa sp.	This study	This study
E. epinepheli Trilles & Justine, 2010	Trilles & Justine (2010)	Trilles & Justine (2010)
E. vulgaris (Stimpson, 1857)	This study	Brusca (1978)
J. malabaricus Aneesh, Helna & Trilles, 2019	Aneesh et al. (2019)	Aneesh et al. (2019)
L. desterroensis Thatcher, Suza-Conceicao & Jost, 2003	Thatcher et al. (2003)	Thatcher et al. (2003)
M. melanosticta (Schioedte & Meinert, 1884)	Bruce (1986)	Stephenson (1969)
