# Supplementary material for: Shape of attachment structures in parasitic isopodan crustaceans: the influence of attachment site and ontogeny
Source: PeerJ. 2020 Jun 18;8:e9181. doi: 10.7717/peerj.9181 (PMC7306222; doi:10.7717/peerj.9181)
Supplement: Supplemental Information 7 [file peerj-08-9181-s007.pdf]

Species	AttachmentSite	Adult_Immature	LegElement	PC1	PC2	PC3	PC4	PC5
A. frontalis	External	Adult	P1 dactylus	7.66E-02	7.89E-02	-6.36E-02	4.30E-03	1.51E-02
A. laticauda	External	Adult	P1 dactylus	-8.61E-02	-1.09E-02	2.04E-02	-1.33E-02	-1.20E-02
A. nemipteri	External	Adult	P1 dactylus	-6.07E-02	1.71E-03	7.01E-03	-2.13E-03	-1.42E-03
A. physodes	External	Adult	P1 dactylus	-5.96E-02	-6.10E-02	8.83E-03	8.54E-03	-4.02E-03
N. acuminata	External	Adult	P1 dactylus	-8.71E-02	5.93E-02	5.05E-02	7.68E-03	1.55E-02
C. frontalis	Buccal	Adult	P1 dactylus	-1.02E-02	-5.85E-02	1.76E-02	2.36E-02	-3.04E-02
C. gaudichaudii	Buccal	Adult	P1 dactylus	-5.55E-03	-9.13E-02	5.53E-03	-1.58E-02	-1.20E-03
C. indica	Buccal	Adult	P1 dactylus	-4.42E-03	-6.47E-03	1.29E-02	1.72E-02	2.65E-02
C. liannae	Buccal	Adult	P1 dactylus	-6.48E-03	-4.66E-02	1.06E-02	1.90E-02	1.02E-02
C. sp	Buccal	Adult	P1 dactylus	-3.28E-02	-9.91E-02	-1.01E-03	-7.11E-03	3.16E-03
E. epinepheli	Branchial	Adult	P1 dactylus	8.15E-03	-5.86E-02	-3.86E-02	-2.59E-02	1.40E-02
E.vulgaris	Branchial	Adult	P1 dactylus	-9.58E-02	8.74E-03	-3.26E-02	7.17E-03	-1.55E-02
J. malabaricus	Branchial	Adult	P1 dactylus	-9.12E-02	4.20E-02	1.65E-02	2.39E-02	1.31E-02
L. desterroensis	Branchial	Adult	P1 dactylus	-1.11E-03	1.13E-01	-4.54E-03	-2.47E-02	-7.69E-04
M. melanosticta	Branchial	Adult	P1 dactylus	8.34E-02	1.34E-02	6.41E-03	-5.03E-03	-5.32E-03
A. frontalis	External	Adult	P6 dactylus	6.60E-02	4.94E-02	-1.07E-02	-1.31E-02	1.61E-02
A. laticauda	External	Adult	P6 dactylus	-4.23E-02	8.64E-02	-1.31E-02	-6.10E-03	9.59E-03
A. nemipteri	External	Adult	P6 dactylus	-2.25E-02	2.74E-02	-2.90E-02	2.24E-03	2.34E-03
A. physodes	External	Adult	P6 dactylus	-2.72E-02	-2.73E-02	-1.15E-02	6.73E-03	1.56E-03
N. acuminata	External	Adult	P6 dactylus	2.34E-02	4.58E-02	1.87E-02	5.73E-03	-6.33E-03
C. frontalis	Buccal	Adult	P6 dactylus	3.28E-02	-2.61E-02	6.96E-02	-8.19E-03	-1.51E-02
C. gaudichaudii	Buccal	Adult	P6 dactylus	4.35E-03	-4.41E-02	-1.86E-03	5.39E-03	-5.76E-03
C. indica	Buccal	Adult	P6 dactylus	-3.21E-02	-4.15E-02	3.61E-02	2.25E-03	2.19E-02
C. liannae	Buccal	Adult	P6 dactylus	9.92E-02	-2.81E-03	1.51E-02	1.28E-02	-1.96E-02
C. sp	Buccal	Adult	P6 dactylus	8.27E-04	-1.01E-01	8.90E-03	-1.12E-02	3.81E-03
E. epinepheli	Branchial	Adult	P6 dactylus	7.70E-02	-6.75E-02	-1.40E-02	-4.15E-02	3.85E-03
E.vulgaris	Branchial	Adult	P6 dactylus	-6.04E-03	-2.96E-02	6.71E-03	1.76E-02	-2.34E-03
J. malabaricus	Branchial	Adult	P6 dactylus	-1.46E-01	1.43E-02	-8.60E-02	-1.02E-02	-3.16E-02
L. desterroensis	Branchial	Adult	P6 dactylus	1.52E-01	2.05E-02	-1.61E-02	2.23E-02	-1.58E-02
M. melanosticta	Branchial	Adult	P6 dactylus	-3.65E-02	-7.66E-02	-1.21E-02	1.65E-04	1.63E-03
A. frontalis	External	Immature	P1 dactylus	2.72E-02	4.06E-02	-2.57E-02	8.53E-03	1.69E-02
A. laticauda	External	Immature	P1 dactylus	-4.95E-02	6.82E-02	-5.26E-03	-2.28E-02	8.66E-03
A. nemipteri	External	Immature	P1 dactylus	-2.65E-02	8.52E-03	2.09E-03	-1.08E-02	1.13E-02
A. physodes	External	Immature	P1 dactylus	-1.18E-01	-1.50E-02	-8.67E-03	9.84E-03	-1.77E-03
N. acuminata	External	Immature	P1 dactylus	-4.40E-02	7.57E-02	2.05E-02	-2.19E-03	-1.07E-02
C. frontalis	Buccal	Immature	P1 dactylus	-1.11E-01	4.20E-02	2.32E-02	7.33E-03	2.89E-03
C. gaudichaudii	Buccal	Immature	P1 dactylus	8.21E-02	-3.75E-02	4.09E-03	-2.10E-02	1.16E-02
C. indica	Buccal	Immature	P1 dactylus	-1.63E-02	2.40E-02	3.66E-02	6.94E-03	1.55E-03
C. liannae	Buccal	Immature	P1 dactylus	1.21E-01	2.67E-02	1.85E-02	1.43E-02	-5.67E-03
C. sp	Buccal	Immature	P1 dactylus	-8.80E-02	1.01E-01	6.61E-02	-1.29E-02	-3.27E-03
E. epinepheli	Branchial	Immature	P1 dactylus	6.51E-02	-5.08E-03	2.04E-02	-1.40E-03	-3.97E-03
E. vulgaris	Branchial	Immature	P1 dactylus	1.58E-02	-8.07E-02	-4.69E-03	-2.70E-03	-1.52E-03
J. malabaricus	Branchial	Immature	P1 dactylus	-9.33E-02	-3.89E-02	-3.09E-02	1.57E-02	2.12E-02
L. desterroensis	Branchial	Immature	P1 dactylus	1.10E-02	3.41E-02	-2.00E-02	-2.05E-03	1.14E-02
M. melanosticta	Branchial	Immature	P1 dactylus	-3.37E-02	1.06E-01	6.32E-02	-4.75E-02	-3.53E-02
A. frontalis	External	Immature	P6 dactylus	4.13E-02	9.85E-02	-1.93E-02	-1.15E-03	-9.78E-03
A. laticauda	External	Immature	P6 dactylus	3.14E-02	4.40E-03	2.79E-02	4.93E-04	4.94E-03
A. nemipteri	External	Immature	P6 dactylus	-6.34E-02	-1.99E-02	-2.25E-02	2.32E-02	1.17E-02
A. physodes	External	Immature	P6 dactylus	8.77E-02	2.79E-02	1.38E-02	-2.19E-03	-1.38E-02
N. acuminata	External	Immature	P6 dactylus	8.94E-02	3.57E-02	-4.15E-04	2.61E-03	-2.37E-03
C. frontalis	Buccal	Immature	P6 dactylus	-1.58E-02	-9.74E-03	-4.91E-02	2.25E-02	-2.24E-02
C. gaudichaudii	Buccal	Immature	P6 dactylus	1.61E-01	-4.21E-02	-6.57E-03	-2.49E-03	-2.61E-03
C. indica	Buccal	Immature	P6 dactylus	-5.92E-02	2.31E-02	-3.02E-02	-8.71E-03	-1.20E-02
C. liannae	Buccal	Immature	P6 dactylus	8.13E-02	3.63E-02	1.79E-03	1.60E-03	3.94E-03
C. sp	Buccal	Immature	P6 dactylus	-3.42E-03	4.45E-02	-6.42E-03	4.79E-03	1.03E-02
E. epinepheli	Branchial	Immature	P6 dactylus	1.16E-01	3.64E-03	-9.73E-03	1.27E-02	-6.67E-04
E. vulgaris	Branchial	Immature	P6 dactylus	8.95E-02	-6.33E-02	1.89E-03	-1.93E-02	1.65E-02
J. malabaricus	Branchial	Immature	P6 dactylus	6.38E-02	1.53E-02	8.36E-03	2.42E-03	-2.57E-03
L. desterroensis	Branchial	Immature	P6 dactylus	1.17E-01	3.08E-02	-4.28E-02	1.95E-02	3.04E-03
M. melanosticta	Branchial	Immature	P6 dactylus	-1.77E-02	4.11E-02	2.06E-02	-1.03E-03	1.59E-02
