# Supplementary material for: Shape of attachment structures in parasitic isopodan crustaceans: the influence of attachment site and ontogeny
Source: PeerJ. 2020 Jun 18;8:e9181. doi: 10.7717/peerj.9181 (PMC7306222; doi:10.7717/peerj.9181)
Supplement: Supplemental Information 10 [file peerj-08-9181-s010.docx]

***Immatures of Cymothoidae in the literature***

We provide here a brief overview of depictions and description of immatures of Cymothoidae available:

1) *Schioedte & Meinert (1884)* provided partial descriptions and drawings (dorsal view) of many immature stages of different species. Over the years, some of these species have been synonymised. The authors provided information on immatures of 39 (sic!) currently valid species. These do not include details of the mouthparts, but give some information regarding antennula, antenna and posterior thorax appendages. To date, there is no publication that has exceeded this number of descriptions of immatures. They provided data on immatures for the following species: *Agarna* (2): *A. carinata* Schioedte & Meinert 1884 (IS1), *A. cumulus* (Haller, 1880) (IS1 described as *Cterissa pterygota*); *Anilocra* (4): *A. longicauda* H. Milne Edwards, 1840 (*nomen dubium*, IS3), *A. gigantea* (Herklots, 1870) (IS2), A. leptosoma Bleeker, 1857 (IS1), *A. physodes* (Linnaeus, 1758) (IS1 and IS3); *Ceratothoa* (7): *C. gaudichaudii* (Milne Edwards, 1840) (*taxon inquirendum*, IS1), *C. italica* Schioedte & Meinert, 1883 (IS2), *C. oestroides* (Risso, 1816) (IS1 and IS2), *C. oxyrrhynchaena* Koelbel, 1878 (IS1), *C. parallela* (Otto, 1828) (IS1 and IS2), *C. verrucosa* (Schioedte and Meinert, 1883) (IS2 described as *Rhexana verrucosa*), *C. trigonocephala* (Leach, 1818) (IS1 and IS2); *Cinusa tetrodontis* Schioedte & Meinert, 1884 (IS2); *Creniola laticauda* (Schioedte and Meinert, 1881) (IS1 described as *Nerocila blainvillei* Schioedte and Meinert, 1881); *Cymothoa* (5): *C. excisa* Perty, 1833 (IS1and IS2), *C. eremita* (Brunnich, 1783) (IS2 and IS3), *C. eximia* Schioedte and Meinert, 1884 (IS1), *C. truncata* Schioedte and Meinert, 1884 (IS2), *C. oestrum* (Linnaeus, 1758) (IS1 and IS2); *Elthusa* (3): *E. vulgaris* (Stimpson, 1857) (IS1 described as *Livoneca vulgaris*), *E. raynaudii* (Milne Edwards, 1840) (IS2 described as *Livoneca raynaudii*), *E. sinuata* (Koelbel, 1879) (IS1 described as *Livoneca sinuata*); *Emetha audouini* (Milne Edwards, 1840) (IS1); *Glossobius* (2): *G. auritus* Bovallius, 1885 (IS1 and IS2 described as *Ceratothoa laticauda* Milne Edwards, 1840) and *G. linearis* (Dana, 1853) (IS1, IS2 and IS3); *Ichthyoxenos jellinghausii* Herklots, 1870 (IS2); *Livoneca redmanii* Leach, 1818 (IS3); *Mothocya* (2): *M. nana* (Schioedte & Meinert, 1884) (IS2 described as *Irona nana*) and *Mothocya renardi* (Bleeker, 1857) (IS2 described as *Irona renardi*); *Nerocila* (3): *N. bivittata* (Risso, 1816) (IS1 and IS2), *N. cephalotes* Schiodte & Meinert, 1881 (IS2), *N. maculata* H. Milne Edwards, 1840 (IS1 and IS2) and *N. neapolitana* (IS1 and IS2) (latter three accepted as *N. orbignyi* (Guérin-Méneville, 1832), *N. orbignyi* (Guérin-Méneville, 1832) (IS2) and *N. serra* Schioedte & Meinert, 1881 (IS1); *Norileca indica* (H. Milne Edwards, 1840) (IS1 described as *Livoneca indica*); *Ourozeuktes bopyroides* (Lesueur, 1814) (IS1 described as *Ourozeuktes monacanthi* Schioedte and Meinert, 1884); *Telotha lunaris* Schioedte and Meinert, 1884 (IS1).

2) *Menzies, Bowmann & Alverson* *(1955)* re-described *Lironeca convexa* Richardson, 1905 (accepted as *Smenispa convexa* (Richardson, 1905)), with a description and drawings of some mouthparts of “young specimens”, with the descriptions stating: “seventh pereopod lacking or present”. No full body illustrations were provided which leads to the interpretation that the specimens could have been from immature stage 1 or 2 and stage 3.

3) *Bowman (1960)* described *Lironeca puhi* (accepted as *Ichthyoxenos puhi* (Bowman, 1962)). It included a basic description of a “juvenile/ young from the marsupium” (which might be either an immature stage 1 or 2) along with some drawings. Another “post-marsupial juvenile” (immature stage 2) is treated, unfortunately without an illustration of the habitus.

4) *Pillai (1964)* provided short descriptions and some drawings of a “larva” of *Agarna tartoor* Pillai, 1954 (accepted as *Joryma tartoor* (Pillai, 1954)), *Indusa malayi* (accepted as *Agarna* *malayi* Tiwari, 1952), *Indusa pustulosa* (accepted as *Agarna pustulosa* (Pillai, 1954)) and *Livoneca circularis* (accepted as *Ryukyua circularis* (Pillai, 1954)). All of these were taken from the marsupium, they are hence either immature stage 1 or 2.

5) *Trilles (1965)* described the “pullus II” (immature stage 2) along with the adult stages of *Anilocra physodes* and *Anilocra frontalis*. Drawings of mouth parts and posterior thorax appendages were included.

6) *Szidat (1966)* provided the life cycle of *Meinertia gaudichaudii* (accepted as *Ceratothoa gaudichaudii*) including drawings and photographs (dorsal view) of a “pre-manca” and “manca” (immature stages 1 and 2 respectively). No descriptions or additional illustrations were provided.

7) *Stephenson (1969)* provided the descriptions and drawings or two “larval” specimens (immature stage 1 and 2) of *Irona melanosticta* (accepted as *Mothocya melanosticta* (Schioedte and Meinert, 1884)). For one of these specimens detailed drawings of mouthparts and some of the posterior thorax appendages were provided.

8) *Brusca (1978a)* provided the life cycle of *Elthusa vulgaris* (Stimpson, 1857) including descriptions of immature stages. Drawings (dorsal view) of a “manca” (immature stage 2) and “juvenile/ aegathoid stage” (immature stage 3) were provided as well as drawings of some posterior thorax appendages and the uropods.

9) *Brusca (1978b)* provided the life cycle of *Nerocila californica* Schioedte & Meinert, 1881, including descriptions of two immature stages: “first, second and third juvenile instars” (immature stage 2) with an illustration, as well as a “juvenile/ aegathoid stage” (immature stage 3), with an illustration. In addition, an illustration of an embryo was provided.

10) *Sandifer & Kerby (1983)* described and illustrated a “newly liberated larva” (immature stage 2) of *Livoneca ovalis* (accepted as *Livoneca redmani* Leach, 1818).

11) *Sartor (1987)* formally described *Cymothoa liannae* and its marsupial development. She also provided descriptions and drawings the embryo, segmented embryo, “pre-manca” and “manca” (immature stage 1 and 2, respectively).

12) *Sartor & Pires (1988)* provided additional data on the post-marsupial development of *Cymothoa liannae* Sartor, 1987, including descriptions and illustrations of the dorsal view of a “juvenile 1” (immature stage 2) and “juvenile 2” (immature stage 3), as well as of some specialised structures.

13) *Stephenson (1987)* provided a short description of “instar I and II” (most likely immatures stages 1 and 2) of *Livoneca neocyttus* (accepted as *Elthusa neocytta* (Avdeev, 1975)), without illustrations.

14) *Segal (1987)* provided a grey-scale photograph of a free-swimming “juvenile” (immature stage 3) of *Nerocila acuminata* Schioedte and Meinert, 1881 in a study on the ecology and behaviour of the immatures of this species.

15) *Adlard & Lester (1995)* provided notes on the life cycle of *Anilocra pomacentri* Bruce, 1987, including illustrations of the dorsal view of a “pre-hatch I” (embryo) and “pre-hatch II” (immature stage 1) and the ventral view of a “manca” (immature stage 2).

17) *Trilles, Colorni & Golani (1999)* described *Livoneca papernea* (nomen dubium) with a dorsal view digital illustration of a “pullus 1” (immature stage 1) specimen along with the adult stages.

18) *Papapanagiotou & Trilles (2001)* provided a greyscale photo of the “manca” (immature stage 2) of *Ceratothoa parallela* in a study that focussed on the effects of this parasite on its host.

19) In 2002, Araújo gave descriptions and digital illustrations of two embryonic stages; an embryo that shows segmented elements; “pre-manca” (immature stage 1) and “manca” (immature stage 2) of unidentified Amazonian representative of Cymothoidae. Digital illustrations of the dorsal view head of eight immature stage 1 specimens were added (*Aniphira* sp., *Asotana magnifica* Thatcher, 1988, *Braga nasuta* Schioedte and Meinert, 1881, *Braga cichlae* Schioedte and Meinert, 1881, *Braga patagonica* Schioedte and Meinert, 1881, and two unnamed species), along with illustrations of mouthparts of an unknown species.

20) *Mladineo (2003)* provided photographs of a “newly hatched stage II pulli” (immature stage 2) individual in dorsal and ventral view of the newly erected species *Ceratothoa oestroides*.

21) With the original description of *Cymothoa catarinensis*, *Thatcher, Jost & Souza-*Conceição *(2003)* included some descriptive notes on a “manca” (immature stage 2) individual of this species and of *C. oestrum*, along with grayscale photgraphs of the dorsal view, pleotelson and uropods of the immature stage 2 of each species.

22) *Thatcher,* Souza-Conceição & Jost *(2003)* described *Lironeca desterroensis* (*taxon inquirendum*) with additional information of a “manca” (immature stage 2), in the form of partial digital illustrations.

23) *Bakenhaster (2004)* provided a noteworthy contribution on the morphology of immature forms of Cymothoidae. This publication contains details on the morphology of a ‘typical’ “manca” (immature stage 2) of Cymothoidae, as well as the description and full set of illustrations of the immature stage 2 of *Livoneca redmanii* Leach, 1818, *Mothocya argenosa* Bruce, 1986, *Nerocila acuminata* Schioedte and Meinert, 1881 and *Olencira praegustator* (Latrobe, 1802). In addition, an identification key was provided with a table of characters that can be used to distinguish between the described immatures.

24) *Bakenhaster, McBride & Price (2006)* provided the development and life cycle of *Glossobius hemiramphi* Williams and Williams, 1985, with an illustration of the embryo, first segmentation stage embryo, “pre-manca” (immature stage 1) and “manca” (immature stage 2). Morphological descriptions of these immatures were not included.

25) *Bariche & Trilles (2006)* described the species *Anilocra pilchardi* with descriptive notes and digital illustrations of an “intermarsupial larva” (immature stage 2).

26) *Thatcher et al. (2007)* described *Cymothoa spinipalpa* with some descriptive information regarding the “manca” (immature stage 2). Three grayscale photographs of this immature were provided: a dorsal view, pleotelson with uropods and mandibular palp.

27) *Trilles (2007)* reviewed and re-described *Olencira praegustator*, adding the descriptions for a “juvenile” (immature stage 3) and “manca” (immature stage 2). A dorsal view illustration was provided for the immature stage 2 individual and a full set of illustrations for the immature stage 3.

28) *Jones et al. (2008)* introduced the term ‘natatory stage’ to characterize the “juvenile” (immature stage 3) of Cymothoidae. Along with this, the authors gave illustrations of selected structures and descriptions of seven immature stage 3 ‘types’, including *Anilocra nemipteri* Bruce, 1987, *Anilocra apogonae* Bruce, 1987, *Cymothoa indica* Schioedte and Meinert, 1884 and *Anilocra longicauda* Schioedte and Meinert, 1881.

29) *Fogelman & Grutter (2008)* presented a comprehensive study on the ecology, life habits and effects of most likely immature stage 2 and 3 individuals of *Anilocra apogonae*, unfortunately without any description or illustration of the studied material.

30) *Trilles & Justine (2010)* described *Elthusa epinepheli* by including a description and illustrations of a “manca” (immature stage 2) individual.

31) *Saito et al. (2014)* provided dorsal view grayscale photos and descriptions of seven “free-swimming forms”(immature stage 2), including that of *Nerocila* sp, *Anilocra* sp, *Mothocya* sp, and 4 unidentified types, all with selected illustrations.

32) *Aneesh et al. (2015)* provided life cycle studies of *Cymothoa frontalis* Milne Edward, 1840, where they included dorsal view photographs of a “pre-manca”, “manca” and “juvenile” (immature stages 1–3 respectively), along with full descriptions and illustrations for these immature individuals. Digital illustrations of embryonic stages of this species were also included.

33) *Aneesh et al. (2016)* recorded and redescribed *Mothocya renardi* (Bleeker, 1857) from the Malabar coast of India. This included a life-cycle representation of the species with the inclusion of the descriptions and illustrations of a “pre-manca”, “manca” and “juvenile” (immature stages 1–3 respectively).

34) *Aneesh et al. (2018)* redescribed *Agarna malayi* Tiwari 1952, supplemented with descriptions and illustrations of “pre-manca”, “manca” and “juvenile” (immature stages 1–3 respectively).

35) *Aneesh et al. (2019)* described, illustrated and provided a photo of the “manca” (immature stage 2) of *Joryma malabaricus*.

*Aegathoa* was erected as a “form” genus and later determined to consist solely of immature stages of other species of Cymothoidae. All, except two of the species within this genus, were transferred to new genera. The remaining two immature species left in *Aegathoa*, are *Aegathoa* *elongata* Monod (1976) and *Aegathoa oculata* (Say, 1818). These have not yet been transferred to a valid genus and is therefore herein only mentioned as unidentified immatures. There are an additional 11 ‘types’ of immatures that have not been identified (see *Jones et al., 2008; Saito et al., 2014*) and 4 immatures that have only been identified to genus level (*Colorni, Trilles & Golani, 1997; Saito et al., 2014*). Many of the immatures from the mentioned publications with detail on immature forms, include only a single view illustration (mostly dorsal) with descriptions that mention only a few plesiomorphic characteristics that do not allow for distinction between species. Recent publications have attempted to provide more detailed descriptions and illustrations of species, with some apomorphic characteristics that can be used to distinguish between immatures of different groups (*Bakenhaster, 2004; Jones et al., 2008; Trilles & Justine, 2010; Aneesh et al., 2015; Aneesh et al., 2016; Aneesh et al., 2018*).

***References***

**Adlard RD, Lester RJG. 1995.** The life-cycle and biology of *Anilocra pomacentri* (Isopoda,

Cymothoidae), an ectoparasitic isopod of the coral-reef fish, *Chromis nitida*

(Perciformes, Pomacentridae). *Australian Journal of Zoology* 43(3):271–281

DOI 10.1071/ZO9950271.

**Aneesh PT, Helna AK, Trilles JP, Chandra K. 2019.** A taxonomic review of the genus *Joryma* Bowman and Tareen, 1983 (Crustacea: Isopoda: Cymothoidae) parasitizing the marine fishes from Indian waters, with a description of a new species. *Marine Biodiversity* 49(3):1449–1478 DOI 10.1007/s12526-018-0920-7.

**Aneesh PT, Sudha K, Helna AK, Anilkumar G. 2016.** *Mothocya renardi* (Bleeker, 1857)

(Crustacea: Isopoda: Cymothoidae) parasitising *Strongylura leiura* (Bleeker) (Belonidae) off the Malabar coast of India: redescription, occurrence and life-cycle. *Systematic Parasitology* 93(6):583–599 DOI 10.1007/s11230-016-9646-8.

**Aneesh PT, Sudha K, Helna AK, Anilkumar G. 2018.** *Agarna malayi* Tiwari 1952 (Crustacea: Isopoda: Cymothoidae) parasitising the marine fish, *Tenualosa toli* (Clupeidae) from India: re-description/description of parasite life cycle and patterns of occurrence. *Zoological Studies* 57:1–22.

**Aneesh PT, Sudha K, Helna AK, Anilkumar G, Trilles J-P. 2015.** *Cymothoa frontalis*, a

cymothoid isopod parasitizing the belonid fish *Strongylura strongylura* from the Malabar Coast (Kerala, India): redescription, description, prevalence and life cycle. *Zoological Studies* 54(1):42 DOI 10.1186/s40555-015-0118-7.

**Araújo CSOD. 2002.** Taxonomia, morfologia e aspectos da biologia reprodutiva dos Cymothoidae (Crustacea: Malacostraca: Isopoda) parasitas de peixes da Amazônia brasileira. D. Phil. thesis, Federal University of Amazonas.

**Bakenhaster MD. 2004.** External morphological features of mancas of four parasitic isopod species (Cymothoidae) in the northern Gulf of Mexico. Ms. thesis, University of Southern Mississippi. 69.

**Bakenhaster MD, McBride RS, Price WW. 2006.** Life history of *Glossobius hemiramphi*

(Isopods: Cymothoidae): development, reproduction, and symbiosis with its host *Hemiramphus brasiliensis* (Pises: Hemiramphidae). *Journal of Crustacean Biology* 26(3):283–294 DOI 10.1651/C-2573.1.

**Bariche M, Trilles JP. 2006.** *Anilocra pilchardi* n. sp., a new parasitic cymothoid isopod from off Lebanon (Eastern Mediterranean). *Systematic Parasitology* 64(3):203–214

DOI 10.1007/s11230-006-9032-z.

**Brusca RC. 1978a.** Studies on the cymothoid fish symbionts of the eastern Pacific (Crustacea:

Isopoda: Cymothoidae). II. Systematics and biology of *Lironeca vulgaris* Stimpson 1857.

Los Angeles: Allan Hancock Foundation.

**Brusca RC. 1978b.** Studies on the Cymothoid Fish Symbionts of the Eastern Pacific

(Isopoda, Cymothoidae) I. Biology of *Nerocila californica*. *Crustaceana* 34:141–154.

**Bowman TE. 1960.** Description and notes on biology of *Lironeca puhi*, n.sp. (Isopoda,

Cymothoidae), parasite of the Hawaiian Moray Eel, *Gymnothorax eurostus* (Abbott).

*Crustaceana* 1(2):84–91 DOI 10.1163/156854060X00131.

**Fogelman RM, Grutter AS. 2008.** Mancae of the parasitic cymothoid isopod, *Anilocra apogonae*: early life history, host-specificity, and effect on growth and survival of preferred young cardinal fishes. *Coral Reefs* 27(3):685–693

DOI 10.1007/s00338-008-0379-2.

**Jones CM, Miller TL, Grutter AS, Cribb TH. 2008.** Natatory-stage cymothoid isopods:

description, molecular identification and evolution of attachment. *International Journal for Parasitology* 38(3–4):477–491 DOI 10.1016/j.ijpara.2007.07.013.

**Mladineo I. 2003.** Life cycle of *Ceratothoa oestroides*, a cymothoid isopod parasite from sea bass *Dicentrarchus labrax* and sea bream *Sparus aurata*. *Diseases of Aquatic Organisms* 57:97–101 DOI 10.3354/dao057097.

**Menzies RJ, Bowman TE, Alverson FG. 1955.** Studies of the biology of the fish parasite

*Livoneca convexa* Richardson (Crustacea, Isopoda, Cymothoidae). *Wasmann Journal of Biology* 13:277–295.

**Papapanagiotou E, Trilles J. 2001.** Cymothoid parasite *Ceratothoa parallela* inflicts great losses on cultured gilthead sea bream *Sparus aurata* in Greece. *Diseases of Aquatic Organisms* 45:237–239 DOI 10.3354/dao045237.

**Pillai NK. 1964.** Parasitic isopods of the family Cymothoidae from South Indian fishes.

*Parasitology* 54(2):211–223 DOI 10.1017/S003118200006786X.

**Saito N, Yamauchi T, Ariyama H, Hoshino O. 2014.** Descriptions and ecological notes of

free-swimming forms of cymothoid isopods (Crustacea: Peracarida) collected in two coastal waters of Japan. *Crustacean Research* 43:1–16 DOI 10.18353/crustacea.43.0_1.

**Sandifer PA, Kerby JH. 1983.** Early life history and biology of the common fish parasite,

*Lironeca ovalis* (Say) (Isopoda, Cymothoidae). *Estuaries* 6(4):420–425

DOI 10.2307/1351401.

**Sartor SM. 1987.** Desenvolvimento marsupial e ciclo de vida de *Cymothoa liannae* Sartor & Pires (Isopoda, Cymothoidae), parasita de peixes. *Boletim do Instituto Oceanográfico* 35(1):43–51 DOI 10.1590/S0373-55241987000100006.

**Sartor SM, Pires AMS. 1988.** The occurrence of *Cymothoa liannae*, a new species of cymothoid isopod from Brazil, with a comparative study of its post-marsupial development. *Crustaceana* 55(2):147–156 DOI 10.1163/156854088X00483.

**Schioedte JC, Meinert FR. 1884.** Symbolae ad monographium cymothoarum crustaceorum

isopodum familiae. IV. Cymothoidae Trib. Il. Cymothoinae. Trib. Ill. Livonecinae.

Naturhistorisk Tidsskrift, Series III 14:221–454.

**Segal E. 1987.** Behavior of juvenile *Nerocila acuminata* (Isopoda, Cymothoidae) during attack,

attachment and feeding on fish prey. *Bulletin of Marine Science* 41:351–360.

**Stephenson A. 1969.** *Irona Melanosticta* (Isopoda: Cymothoidae): a new record for New Zealand waters, with descriptions of male, female and larval states. *Records of the Auckland Institute and Museum* 6:427–434.

**Stephenson A. 1987.** Additional notes on *Livoneca neocyttus* (Isopoda: Cymothoidae). *Records of the Auckland Institute and Museum* 24:135–142.

**Szidat L. 1966.** Untersuchungen über den Entwicklungszyklus von *Meinertia gaudichaudii* (Milne Edwards, 1840) Stebbing, 1886 (Isopoda, Cymothoidae) und die Entstehung eines sekundären Sexualdimorphismus bei parasitischen Asseln der Familie Cymothoidae Schioedte u. Meinert, 1881. *Parasitology Research* 27:1–24.

**Thatcher VE, Araujo GS, de Lima JTAX, Chellappa S. 2007.** *Cymothoa spinipalpa* sp. nov.

(Isopoda, Cymothoidae) a buccal cavity parasite of the marine fish, *Oligoplites saurus* (Bloch & Schneider) (Osteichthyes, Carangidae) of Rio Grande do North State, Brazil. *Revista Brasileira de Zoologia* 24(1):238–245 DOI 10.1590/s0101-81752007000100032.

**Thatcher VE, Souza-Conceição JM, Jost GF. 2003.** *Lironeca desterroensis* sp. nov. (Isopoda,

Cymothoidae) from the gills of a marine fish, *Cetengraulis edentulus* Cuvier, of Santa Catarina Island, Brazil. *Revista Brasileira de Zoologia* 20(2):251–255

DOI 10.1590/S0101-81752003000200013.

**Thatcher VE, Jost GF, Souza-Conceição JM. 2003.** Comparative morphology of *Cymothoa* spp. (Isopoda, Cymothoidae) from Brazilian fishes, with the description of *Cymothoa catarinensis* sp. nov. and redescriptions of *C. excisa* Perty and *C. oestrum* (Linnaeus). *Revista Brasileira de* *Zoologia* 20(3):541–552 DOI 10.1590/S0101-81752003000300028.

**Trilles J-P. 1965.** Sur deux espèces d’Anilocres (Isopodes, Cymothoidae) mal connues:

*Anilocra physodes* (L.) et *Anilocra frontalis* (Milne Edwards). *Annales de Parasitologie Humaine et Comparée* 40(5):575–594 DOI 10.1051/parasite/1965405575.

**Trilles J-P. 2007.** *Olencira praegustator* (Crustacea, Isopoda, Cymothoidae) parasitic on Brevoortia species (Pisces, Clupeidae) from the southeastern coasts of North America: review and redescription. *Marine Biology Research* 3(5):296–311

DOI 10.1080/17451000701543439.

**Trilles J-P, Colorni A, Golani D. 1999.** Two species and new record of Cymothoid Isopods from the Red Sea. *Cahiers de Biologie Marine* 40:1–14.

**Trilles J-P, Justine J-L. 2010.** *Elthusa epinepheli* sp. nov. (Crustacea, Isopoda, Cymothoidae) a branchial parasite of the grouper *Epinephelus howlandi* (Serranidae, Epinephelinae) from off New Caledonia. *Acta Parasitologica* 55(2):177–187

DOI 10.2478/s11686-010-0020-8.
